# COPI vesicle formation and *N*-myristoylation are targetable vulnerabilities of senescent cells

**DOI:** 10.1038/s41556-023-01287-6

**Published:** 2023-11-27

**Authors:** Domhnall McHugh, Bin Sun, Carmen Gutierrez-Muñoz, Fernanda Hernández-González, Massimiliano Mellone, Romain Guiho, Imanol Duran, Joaquim Pombo, Federico Pietrocola, Jodie Birch, Wouter W. Kallemeijn, Sanjay Khadayate, Gopuraja Dharmalingam, Santiago Vernia, Edward W. Tate, Juan Pedro Martínez-Barbera, Dominic J. Withers, Gareth J. Thomas, Manuel Serrano, Jesús Gil

**Affiliations:** 1 MRC Laboratory of Medical Sciences (LMS), London, UK; 2 Institute of Clinical Sciences (ICS), Faculty of Medicine, Imperial College London, London, UK; 3 Institute for Research in Biomedicine (IRB Barcelona), The Barcelona Institute of Science and Technology (BIST), Barcelona, Spain; 4 Department of Pulmonology, ICR, Hospital Clinic, August Pi i Sunyer Biomedical Research Institute (IDIBAPS), Universitat de Barcelona, Barcelona, Spain; 5 Instituto de Investigaciones Biomédicas August Pi i Sunyer (IDIBAPS), Barcelona, Spain; 6 School of Cancer Sciences, Faculty of Medicine, University of Southampton, Southampton, UK; 7 Developmental Biology and Cancer Programme, Birth Defects Research Centre, Great Ormond Street Institute of Child Health, University College London, London, UK; 8 Karolinska Institute, Department of Biosciences and Nutrition, Huddinge, Sweden; 9 Department of Chemistry, Molecular Sciences Research Hub, London, UK; 10 The Francis Crick Institute, London, UK; 11 Altos Labs, Cambridge Institute of Science, Granta Park, UK; 12 Present Address: AstraZeneca, Immuno-Oncology Discovery, Oncology R&D, Cambridge, UK

**Keywords:** Cell biology, Cell growth, Senescence

## Abstract

Drugs that selectively kill senescent cells (senolytics) improve the outcomes of cancer, fibrosis and age-related diseases. Despite their potential, our knowledge of the molecular pathways that affect the survival of senescent cells is limited. To discover senolytic targets, we performed RNAi screens and identified coatomer complex I (COPI) vesicle formation as a liability of senescent cells. Genetic or pharmacological inhibition of COPI results in Golgi dispersal, dysfunctional autophagy, and unfolded protein response-dependent apoptosis of senescent cells, and knockdown of COPI subunits improves the outcomes of cancer and fibrosis in mouse models. Drugs targeting COPI have poor pharmacological properties, but we find that *N*-myristoyltransferase inhibitors (NMTi) phenocopy COPI inhibition and are potent senolytics. NMTi selectively eliminated senescent cells and improved outcomes in models of cancer and non-alcoholic steatohepatitis. Our results suggest that senescent cells rely on a hyperactive secretory apparatus and that inhibiting trafficking kills senescent cells with the potential to treat various senescence-associated diseases.

## Abstract (web summary)

McHugh et al. identify COPI trafficking as a vulnerability of senescent cells. *N*-myristoyltransferase inhibitors phenocopy COPI inhibition and are potent senolytics.

## Main

Senescence is a cellular response induced by stresses such as replicative exhaustion, oncogenic activation or genotoxic agents. Following the induction of senescence, cells enter a stable cell-cycle arrest, a process mediated by the upregulation of cyclin-dependent kinase inhibitors, such as p16^INK4a^ and p21^CIP1^^1^. Senescent cells also undergo multiple phenotypic changes, including altered morphology, chromatin remodelling, organelle reorganization, altered metabolism and the production of a bio-active secretome known as the senescence-associated secretory phenotype (SASP)^2^.

Acute induction of senescence is a protective response that, by restricting the replication of damaged cells, limits cancer and fibrosis. However, senescent cells accumulating during aging contribute to many pathologies^3^. The selective killing of p16^INK4a^-positive senescent cells (senolysis) in aged normal mice improves healthspan, increases lifespan^4^ and also alleviates pathologies such as atherosclerosis^5^, osteoarthritis^6^ and neurodegenerative diseases^7^.

These observations have made the prospect of senolytic therapies attractive^8^. Several senolytics have been identified, including dasatinib and quercetin (referred to as D + Q)^9^, Bcl2 family inhibitors such as ABT-263 and ABT-737^10–12^, a modified FOXO4-p53 interfering peptide^13^, HSP90 inhibitors^14^, cardiac glycosides^15,16^ and β-galactosidase-activated nanoparticles and pro-drugs^17–19^. First-in-human studies have validated the potential of senolytics to decrease senescence burden in human patients^20–22^. However, the failure of the Phase 2 clinical trial of a senolytic MDM2 inhibitor against osteoarthritis^23^ highlights the need to identify more effective and specific senolytics. To this end, we need to comprehensively uncover the molecular pathways promoting the survival of senescent cells.

In this Article we report the performance of unbiased genetic screens to discover senolytic targets. Our findings reveal coatomer complex I (COPI) signalling and *N*-myristoylation as previously unknown and targetable vulnerabilities of senescent cells that can be exploited to treat senescence-associated pathologies.

## Results

### Small-interfering RNA screens identify senolytic targets

**Fig. 1:**
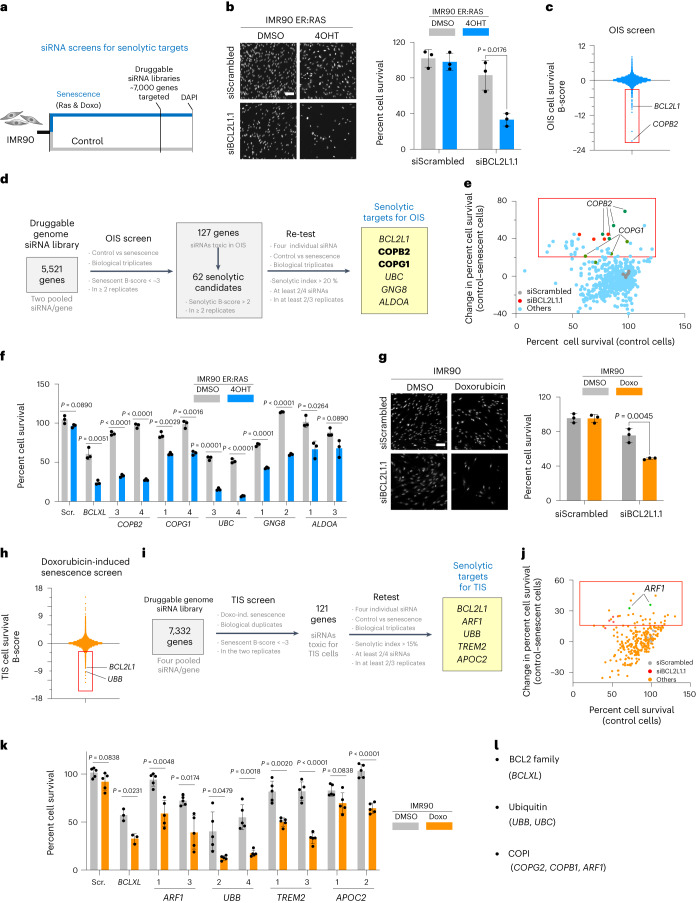
RNAi screens identify senolytic targets. **a**, Experimental design for the RNAi screens to identify senolytic targets. **b**, Right: quantification of cell survival of senescent (4OHT) and control (DMSO) IMR90 ER:RAS cells three days post-transfection with *BCL2L1* siRNA (*n* = 3). Left: representative DAPI-stained immunofluorescence (IF) images. Scale bar, 100 µm. **c**, Results of the primary siRNA screen for senolytic targets in OIS. Normalized cell counts are shown as mean B-score, reflecting counts normalized to account for plate positional effects using the B-scoring method. A candidate was considered a hit if the B-score in ≥2 replicates was <−3. **d**, Summary of the siRNA screen for senolytic targets in OIS, genes in the same pathway are indicated in bold. **e**, Re-test of OIS screen candidates. A candidate was considered a hit if the change in % cell survival was >20 with siRNAs in ≥2 replicates. **f**, Percentage cell survival in the context of OIS (4OHT) and control (DMSO) cells (*n* = 3). The data represent the deconvolution of values shown in **e**. **g**, Right: quantification of cell survival of doxorubicin-induced senescent (Doxo) and control (DMSO) IMR90 cells three days post-transfection with *BCL2L1* siRNA (*n* = 3). Left: representative DAPI IF images. Scale bar, 100 µm. **h**, Results of the primary siRNA screen for senolytic targets in doxorubicin-induced senescence. Normalized cell counts are shown as mean B-score. A candidate is considered a hit if the B-score was <−3 in ≥2 replicates. **i**, Summary of the siRNA screen for senolytic targets in doxorubicin-induced senescence. **j**, Re-test of TIS screen candidates. A candidate was considered a hit if the change in % cell survival was >15 with siRNAs in ≥2 replicates. **k**, Percentage cell survival of doxorubicin-induced senescence (Doxo) and control (DMSO) cells (*n* = 6 for DMSO- and 4OHT-treated cells, *n* = 3 for *BCLXL* siRNA transfected cells). Data represent the deconvolution of values shown in **j** with additional replicates. **l**, Common pathways identified in the siRNA screen for senolytic targets. Data in **b**, **f**, **g** and **k** are presented as mean ± s.d. (unpaired, two-tailed Student’s *t*-test). *n* represents independent experiments in **b**, **f**, **g**, **k**. Data in **c** and **h** is representative of three replicates. Data are presented as percentage cell survival in control cells versus the difference in cell survival between control and senescent cells in **e** and **j**. Source numerical data are available as source data. Source data

**Extended Data Fig. 1:**
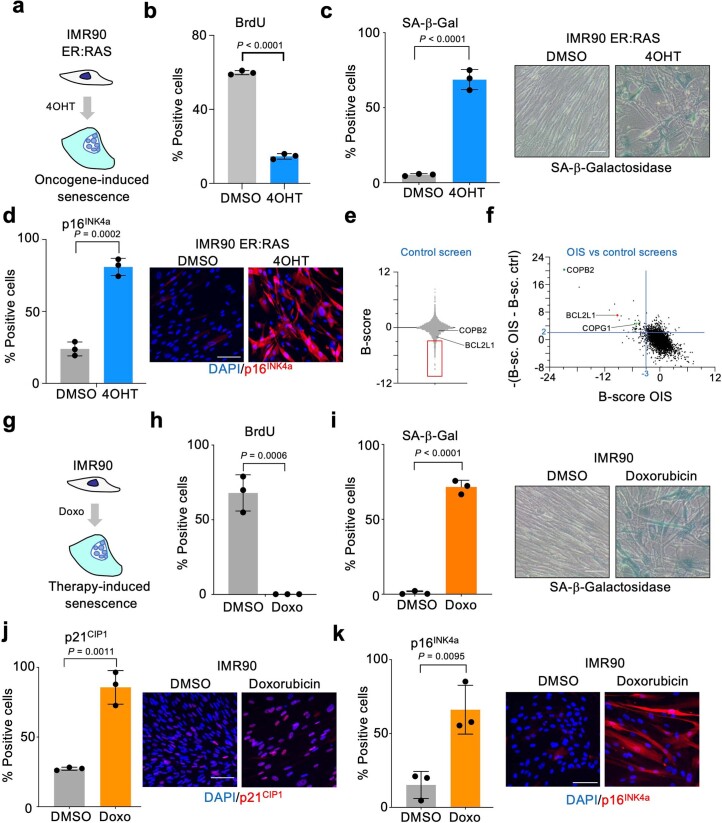
Models for oncogene- and therapy-induced senescence. **a**, IMR90 ER:RAS model for oncogene-induced senescence (OIS). Addition of 4-OHT activates ER:RAS, triggering OIS. **b**, Quantification of IF staining for BrdU incorporation in IMR90 ER:RAS cells 5 days after treatment with 4-OHT or DMSO (n = 3). **c**, Percentage of cells staining positive for SA-β-galactosidase activity in IMR90 ER:RAS cells 5 days after treatment with 4-OHT or DMSO (n = 3). Representative brightfield image (right). Scale bar, 100 µm. **d**, Percentage of cells staining positive for p16^INK4a^ by IF in IMR90 ER:RAS cells 5 days after treatment with 4-OHT or DMSO (n = 3). Representative IF image (right). Scale bar, 100 µm. **e**, B-score normalization of cell numbers shown for the siRNA screen performed on control, non-senescent (DMSO treated) IMR90 ER: RAS cells. Points show normalized values for 3 technical replicates. Red box indicates samples with a B-score < 3. **f**, Screen results for control (DMSO-treated IMR90 ER:RAS) and OIS (4OHT-treated IMR90 ER:RAS). Graph displays B-score in OIS versus the negative of the difference in B score between OIS and control screens. Points show mean normalized values for 3 technical replicates with cut-offs shown for OIS B-score < -3 and a difference in B score of > 2 between control and OIS cells. **g**, Model of therapy-induced senescence (TIS). Senescence was induced by 7 days of doxorubicin treatment in IMR90 cells. **h**, Quantification of IF staining for BrdU incorporation in IMR90 cells 2 days after treatment with doxorubicin or DMSO (n = 3). **i**, Percentage of IMR90 cells staining positive for SA-β-galactosidase activity 7 days treating with doxorubicin or DMSO (n = 3). Representative brightfield images (right). Scale bar, 100 µm. **j-k**, Percentage of IMR90 cells staining positive for p21^CIP1^ (j) or p16^INK4a^ (k), 6 (j) or 3 (k) days by IF after treating with doxorubicin or DMSO (n = 3). Representative IF image (right, j & k). Scale bar, 100 µm. Data represented as mean ± SD unless otherwise stated. Statistical tests performed using unpaired two-tailed t-test unless. N represents independent experiments. Source numerical data are available in source data. Source data

To discover vulnerabilities associated with senescence, we performed large-scale small-interfering RNA (siRNA) screens in normal and senescent human cells (Fig. 1a). We first used IMR90 fibroblasts expressing an ER:RAS fusion protein. In the presence of 4-hydroxytamoxifen (4OHT), ER:RAS becomes activated, inducing oncogene-induced senescence (OIS; Extended Data Fig. 1a–d). Transfection with siRNAs and their associated knockdown were equally efficient in senescent and non-senescent cells. Moreover, depletion of *BCL2L1* (also known as *Bcl-XL*) preferentially killed cells undergoing OIS, acting as a screen control (Fig. 1b and Supplementary Fig. 1).

We screened a ‘druggable genome’ siRNA library targeting around 5,500 genes in IMR90 ER:RAS cells and compared the effects of siRNAs on the viability of normal and senescent cells. We identified 127 genes for which knockdown killed 4OHT-treated cells undergoing OIS (Fig. 1c,d); 62 of these genes were not essential for viability in control (dimethyl sulfoxide (DMSO)-treated) cells (Extended Data Fig. 1e,f and Fig. 1d). We then performed a secondary screen with a library comprising four independent siRNAs targeting each of the candidates (Fig. 1e,f) and confirmed six genes (*BCL2L1*, *COPB2*, *COPG1*, *UBC*, *GNG8* and *ALDOA*) for which knockdown selectively killed cells undergoing OIS, but not non-senescent cells (Fig. 1f).

To identify senolytic targets relevant to different types of senescence, we treated IMR90 cells with doxorubicin to model chemotherapy-induced senescence (TIS; Extended Data Fig. 1g–k). Knockdown of *BCL2L1* preferentially killed cells undergoing TIS (Fig. 1g). We screened a library targeting over 7,300 genes and identified that siRNAs against 121 genes killed cells undergoing doxorubicin-induced senescence (Fig. 1h,i). A secondary screen confirmed that siRNAs targeting five of those genes (*BCL2L1*, *ARF1*, *UBB*, *TREM2* and *APOC2*) preferentially killed cells undergoing TIS when compared to normal cells (Fig. 1i–k). Overall, we identified ten known or putative senolytic targets from both screens. *BCL2L1* together with ubiquitins (*UBB* and *UBC*) and components of the coatomer complex I (COPI) pathway (*COPB2*, *COPG1* and *ARF1*) were identified in both screens (Fig. 1l), suggesting that they constitute general vulnerabilities associated with senescence.

### COPI is a liability of senescent cells

**Fig. 2:**
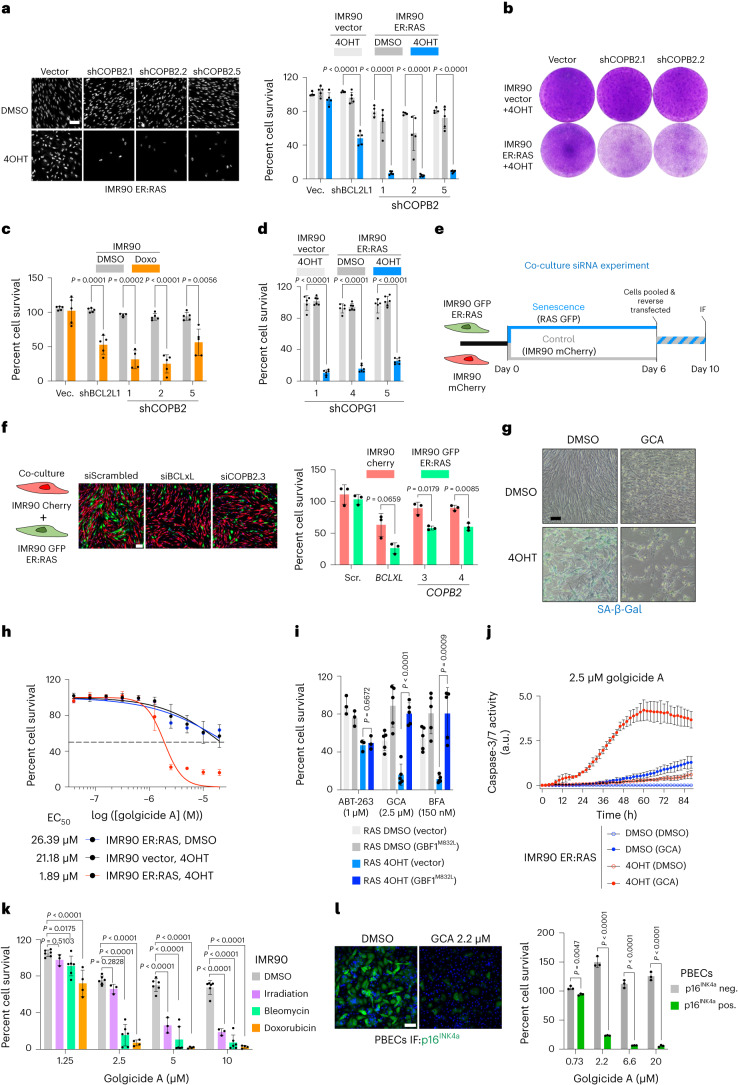
COPI is a vulnerability of senescent cells. **a**, Right: percentage cell survival of the indicated senescent (4OHT) and control (DMSO) IMR90 ER:RAS cells (*n* = 4, IMR90 Vector+4OHT; *n* = 5, other groups). Left: representative DAPI-stained IF images. Scale bar, 100 µm. **b**, Crystal violet staining. An image representative of three independent experiments is shown. **c**, Senolytic activity of *COPB2* in doxorubicin-induced senescence in IMR90 cells (*n* = 4 shCOPB2.1, *n* = 5 other shRNAs). **d**, Senolytic activity of *COPG1* depletion during OIS in IMR90 ER:RAS cells (*n* = 5, IMR90 Vector+4OHT; *n* = 6, other groups). **e**, Schematic outlining the strategy of co-culture senolytic testing of *COPB2* siRNAs. **f**, Right: percentage survival in a co-culture experiment of IMR90 green fluorescent protein (GFP) ER:RAS with IMR90 Cherry cells transfected with the indicated siRNAs. Cell numbers were determined from counts of mCherry or GFP-positive cells detected by IF (*n* = 3). Left: representative IF images. Scale bar, 100 µm. **g**, Representative images of IMR90 ER:RAS cells seven days post addition of 4OHT and stained for Senescence-Associated (SA)-β-Gal activity 72 h after treatment with 2.5 µM GCA (*n* = 3). Quantification is shown in Extended Data Fig. 2a. Scale bar, 100 µm. **h**, Dose–response curves for the senolytic effect of the GBF1 inhibitor GCA in the context of OIS (*n* = 6). Red, IMR90 ER:RAS +4OHT; Black, IMR90 vector +4OHT; Blue, IMR90 ER:RAS +DMSO. **i**, Percentage survival of control cells (RAS DMSO) and oncogene-induced senescent cells (RAS 4OHT) transduced with vectors and treated with ABT-263, GCA or BFA (*n* = 5 for GCA/BFA; *n* = 3, ABT-263). **j**, Caspase-3/7 activity in control (DMSO) or senescent (4OHT) cells after treatment with DMSO or 2.5 µM GCA seven days after senescence induction (*n* = 3). **k**, Senolytic activity of GCA in senescence induced by irradiation (*n* = 3), bleomycin and DMSO (*n* = 6), and doxorubicin (*n* = 4). Data are presented as mean ± s.d. Comparisons to the corresponding DMSO-treated cells (grey bars) with two-way analysis of variance (ANOVA). **l**, Right: percentage cell survival of p16^INK4a^ positive and negative cells in PBECs after treatment with GCA or vehicle (DMSO). Left: representative p16^INK4a^ (green)-stained IF images. Scale bar, 50 μm. *n* = 3. Data in all figures are presented as mean ± s.d. *n* represents independent experiments throughout the figure. Unpaired, two-tailed, Student’s *t*-test was used unless otherwise stated. Source numerical data are available as source data. Source data

COPI is involved in the retrograde transport of vesicles from the Golgi to the endoplasmic reticulum (ER) and it regulates other membrane-trafficking events^24^. To confirm the COPI complex as a vulnerability of senescent cells, we used three independent short-hairpin RNAs (shRNA) to deplete *COPB2* (Supplementary Fig. 2a). Knockdown of *COPB2* preferentially killed cells undergoing OIS (Fig. 2a,b). *COPB2* depletion also killed IMR90 cells undergoing doxorubicin-induced senescence (Fig. 2c). COPI-coated vesicles consist of multiple subunits^25^, and depletion of the COPI subunit *COPG1* (Supplementary Fig. 2b) also killed IMR90 cells undergoing OIS and doxorubicin-induced senescence (Fig. 2d and Supplementary Fig. 2c). Moreover, depletion of *COPB2* is also senolytic in co-cultures of normal and senescent cells (Fig. 2e,f).

**Extended Data Fig. 2:**
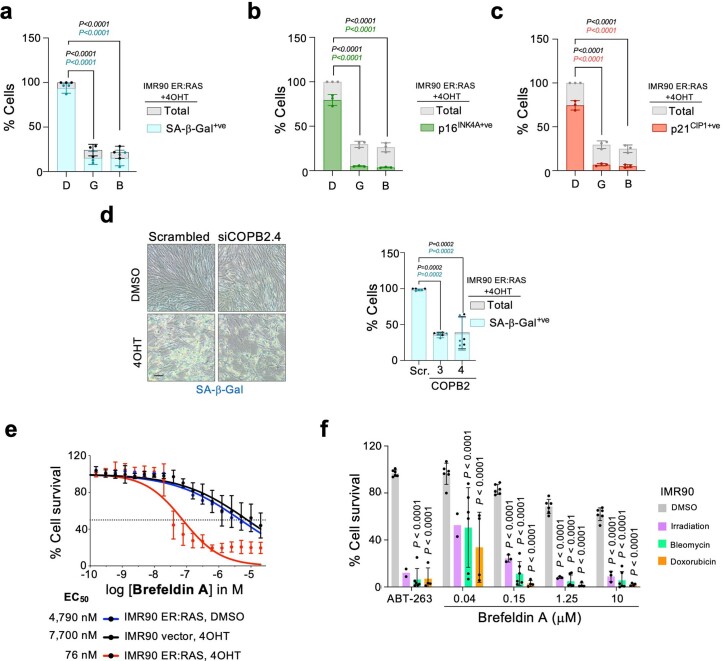
COPI inhibitors are senolytic. **a-c**, IMR90 ER:RAS cells were treated 7 days after senescence induction with either DMSO (D), 2.5 µM golgicide A (G), or 150 nM brefeldin A (B) for 72 h, and cells fixed and stained as indicated. Percentage of either total cells or cells positive for SA-β-galactosidase (a), p16^INK4a^ (b), and p21^CIP1^ (c) relative to the total number of DMSO-treated cells. (n = 3 replicates). Two-way ANOVA. The significance of total cell comparisons is shown in black, while the significance for comparisons of positive for SA-β-galactosidase (a), p16^INK4a^ (b), and p21^CIP1^ (c) are shown in blue, green, and red respectively. Percentage cells represent a fraction of cells compared to the total number of cells in the DMSO-treated sample for each replicate. **d**, IMR90 ER:RAS cells were transfected with the indicated siRNAs 7 days after treatment with 4OHT (senescent) or DMSO (controls). Cells were fixed and stained for SA-β-galactosidase activity 72 h after transfection. Representative images (left). Scale bar, 100 µm. Quantification (right) of a relative number of senescent cells. Shown are the percentage of total or SA-β-gal-positive cells relative to the total number of DMSO-treated cells. (n = 3). Scr. scrambled. Two-way ANOVA. Percentage cells represent a fraction of cells compared to the total number of cells in the DMSO-treated sample for each replicate. **e**, Dose-response curves for senolytic effect of GBF1 inhibitor brefeldin A in the context of OIS (Dose 20 µM – 40 nM, n = 6, 20 nM – 40pM, n = 3 IMR90 vector + 4OHT, n = 4 IMR90 ER:RAS + DMSO and +4OHT). EC_50_, half maximal effective concentration. **f**, Senolytic activity of brefeldin A in the context of senescence induced by irradiation (n = 3, n = 2 for ABT-263 and 40 nM BFA treatment), bleomycin (n = 6) or doxorubicin (n = 4). Data represented as mean ± SD. Comparisons are to the corresponding DMSO-treated cells (gray bars) using two-way ANOVA. Data represented as mean ± SD throughout figure. N represents independent experiments throughout figure. Source numerical data are available in source data. Source data

The formation of COPI vesicles is regulated by the ARF family of GTPases^26^. Drugs such as brefeldin A (BFA^27^) and golgicide A (GCA^28^) interfere with COPI vesicle formation by inhibiting GBF1, a guanine nucleotide exchange factor required to activate ARF GTPases. BFA and GCA treatments selectively killed cells undergoing OIS, as assessed by quantifying their effect in senescent cells (as assessed by SA-β-galactosidase (SA-β-Gal) or immunofluorescence (IF) against p16^INK4a^ or p21^CIP1^; Fig. 2g and Extended Data Fig. 2a–c). Similarly, SA-β-Gal staining also confirmed that *COPB2* depletion targeted senescent (SA-β-Gal-positive) cells (Extended Data Fig. 2d).

Importantly, the half-maximal effective concentration (EC_50_) values for BFA and GCA were around 60-fold and 11-fold lower, respectively, for senescent cells compared to normal cells (Fig. 2h and Extended Data Fig. 2e). BFA inhibits multiple guanine nucleotide exchange factors, whereas GCA is a specific GBF1 inhibitor^28^. To determine whether the senolytic effects of GCA and BFA are due to on-target GBF1 inhibition, we expressed a GBF1 mutant with reduced binding to these drugs (GBF1^M832L^)^28^. GBF1^M832L^ abrogated the senolytic effects of GCA and BFA, but not of the BCL2 family inhibitor ABT-263 (Fig. 2i). Treatment of senescent cells with GBF1 inhibitors induced caspase-3/7 activity (Fig. 2j), and the death of senescent cells could be prevented by the pan-caspase inhibitor QVD, but not inhibitors of pyroptosis (YVAD or VX-765), necroptosis (Nec-1) or ferroptosis (Liprox; Supplementary Fig. 3a,b). We obtained similar results with the knockdown of *COPB2* (Supplementary Fig. 3c,d), suggesting that COPI inhibition selectively induces apoptosis in senescent cells.

We took advantage of IMR90 cells undergoing senescence due to treatment with bleomycin, doxorubicin or irradiation (Supplementary Fig. 4a) to further confirm the senolytic potential of GBF1 inhibitors (Fig. 2k and Extended Data Fig. 2f). GCA and BFA also killed other cell types undergoing bleomycin-induced senescence, such as normal human lung fibroblasts (NHLFs; Supplementary Fig. 4b,c) or primary bronchial epithelial cells (PBECs; Supplementary Fig. 4d,e). GCA (Fig. 2l) or BFA (Supplementary Fig. 4f) also eliminated senescent cells in mid-passage cultures of PBECs containing senescent (p16^INK4a^-positive) and normal (p16^INK4a^-negative) cells. These results demonstrate that COPI is a vulnerability of senescent cells and that GBF1 inhibitors behave as broad-spectrum senolytics.

### *COPB2* knockdown disrupts Golgi triggering an unfolded protein response in senescence

**Fig. 3:**
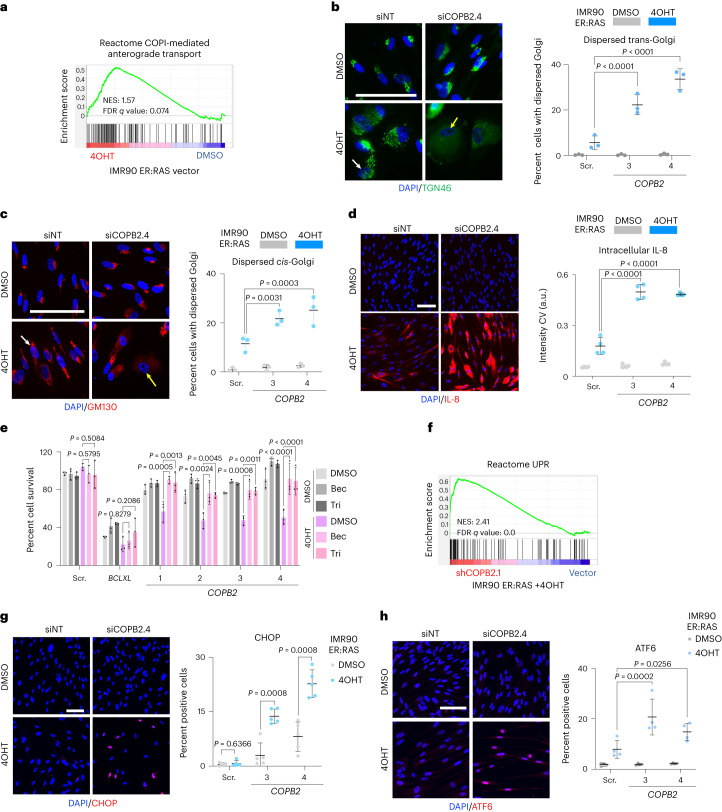
*COPB2* depletion causes Golgi disruption and triggers the UPR in senescent cells. **a**, GSEA plot for COPI transport in cells undergoing OIS. NES, normalized enrichment score; FDR, false discovery rate. **b**, Right: percentage of dispersed *trans*-Golgi by IF in senescent (4OHT) and control (DMSO) IMR90 ER:RAS cells transfected with the indicated siRNAs (*n* = 3). Quantification was performed using organelle count (Methods). Left: representative IF images. The white arrow points to a cell with a normal *trans*-Golgi, and the yellow arrow indicates a cell with dispersed *trans*-Golgi. Scale bar, 100 µm. **c**, Right: percentage of dispersed *cis*-Golgi by IF in senescent (4OHT) and control (DMSO) IMR90 ER:RAS cells transfected with the indicated siRNAs (*n* = 3). Quantification was performed using the integrated intensity threshold (intensity × area) in the ‘region growing’ collar. Left: representative IF images. Normal *cis*-Golgi (white arrow) and dispersed *cis*-Golgi (yellow arrow) are indicated. Scale bar, 100 µm. **d**, Right: quantification of intracellular levels of IL-8 in senescent (4OHT) and control (DMSO) IMR90 ER:RAS cells after transfection with the indicated siRNAs by measurement of the pixel intensity coefficient of variance (CV) within cytoplasmic collar (*n* = 4). Left: representative IF images. Scale bar, 100 µm. Statistical tests were performed using two-way ANOVA relative to DMSO-treated cells. **e**, SASP inhibition caused by treatment with 10 µM glucocorticoids (Bec, beclomethasone; Tri, triamcinolone) prevents senolysis induced by *COPB2* depletion. Quantification of cell survival is shown for senescent (4OHT) and control (DMSO) IMR90 ER:RAS cells treated as indicated (*n* = 3). **f**, GSEA plot showing that an UPR gene signature is enriched in IMR90 ER:RAS upon *COPB2* depletion. **g**,**h**, Right: percentage of cells positive for nuclear CHOP (**g**, *n* = 5) and nuclear ATF6 (**h**, *n* = 4) by IF six days after treating with 4OHT (to induce OIS) or DMSO (as control) for cells transfected with the indicated siRNAs. Staining was performed 72 h later. Left: representative IF images. Scale bar, 100 µm. Unpaired two-tailed Student’s *t*-test was used for statistical comparison in **g**. All data are presented as mean ± s.d. *n* represents independent experiments throughout. Statistical tests were performed using two-way ANOVA against scrambled siRNA unless otherwise stated. Source numerical data are available as source data. Source data

**Extended Data Fig. 3:**
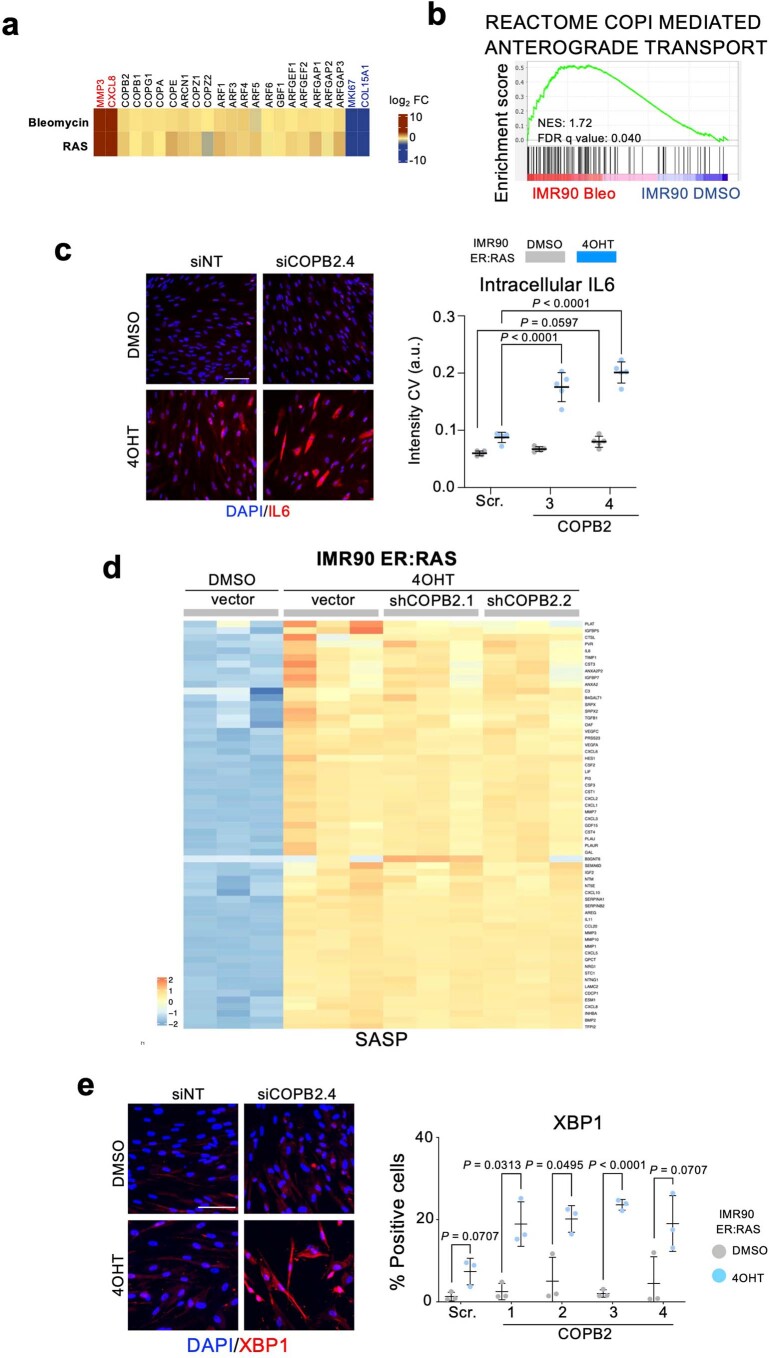
Effects of COPB2 depletion on senescent cells. **a**, Heatmap derived from RNA-Seq data showing the relative mRNA levels of COPI components (in black) in senescent cells. As a reference, relative levels of MMP3 and CXCL8 (upregulated during senescence in red) and MKI67 and COL15A1 (downregulated during senescence, in blue) are included. Represented are the log_2_Fold change (FC) in oncogene-induced senescent (RAS) or therapy-induced senescent (BLEO) IMR90 ER:RAS or IMR90 cells transduced with shRNA vector, induced to senesce for 10 days and measured relative to respective cell lines treated with DMSO (n = 3). **b**, GSEA plot of COPI-mediated transport signature in bleomycin treated IMR90 10 days post senescence induction. NES, normalized enrichment score; FDR, false discovery rate. **c**, Quantification of intracellular levels of IL6 in senescent (4OHT) and control (DMSO) IMR90 ER:RAS cells after transfection with the indicated siRNAs (n = 4). Representative IF image (left) Scale Bar, 100 µm. Statistical test performed using two-way ANOVA. **d**, Heatmap derived of RNA-Seq data showing the relative mRNA levels of SASP components in the indicated cells. Data represented as row z-score normalized. (n = 3). **e**, Quantification of percentage nuclear XBP1 positive by IF in senescent (4OHT) and control (DMSO) IMR90 ER:RAS cells after transfection with the indicated siRNAs (n = 3). Representative IF image (left). Scale Bar, 100 µm. Unpaired two-tailed, student’s t-test. Data is represented throughout the figure as mean ± SD unless otherwise stated. N represents number of independent experiments throughout figure. Source numerical data are available in source data. Source data

COPI inhibition disrupts the *cis*- and *trans*-Golgi compartments, as well as the early endosome, impairing protein secretion and autophagy^29^. To understand the selective sensitivity of senescent cells to COPI inhibition, we first conducted RNA-sequencing (RNA-seq) analysis of IMR90 cells undergoing OIS or bleomycin-induced senescence. Although we did not observe any significant and substantial upregulation in the expression of COPI structural or regulatory subunits on senescent cells (Extended Data Fig. 3a), gene set enrichment analysis (GSEA) found a COPI gene signature enriched on senescent cells, suggesting a higher reliance on the pathway (Fig. 3a and Extended Data Fig. 3b).

Next, we knocked down *COPB2* and examined the integrity and morphology of the Golgi using antibodies against proteins in the *trans*-Golgi (TGN46; Fig. 3b) and the *cis*-Golgi (GM130; Fig. 3c). In agreement with previous observations^30^, cells undergoing OIS displayed a reorganized, more scattered Golgi (Fig. 3b). Importantly, *COPB2* knockdown resulted in Golgi dispersal in senescent but not normal cells. We identified an increase in the percentage of cells with a dispersed Golgi as assessed by TGN46 or GM130 staining (Fig. 3b,c). These results suggest that the Golgi apparatus is disrupted upon *COPB2* knockdown in cells undergoing OIS.

The reorganized Golgi of senescent cells most probably reflects their enhanced need to produce, traffic and recycle proteins required for the senescent program, including the SASP^30^. We reasoned that disrupting the Golgi in senescent cells could trigger an accumulation of aberrant proteins, including intracellular accumulation of otherwise secreted factors. Cells undergoing OIS showed an increase in the intracellular levels of cytokines such as interleukin-8 (IL-8) (Fig. 3d) or IL-6 (Extended Data Fig. 3c), reflecting SASP production. Strikingly, intracellular levels of IL-8 and IL-6 were much higher in senescent cells upon *COPB2* depletion (Fig. 3d and Extended Data Fig. 3c), and these changes were not due to a transcriptional increase of SASP components, as messenger RNA (mRNA) levels of SASP components were unaffected by *COPB2* knockdown (Extended Data Fig. 3d).

Glucocorticoids (such as beclomethasone or triamcinolone) inhibit the SASP^31^ without preventing senescence (Supplementary Fig. 5a–d). Interestingly, glucocorticoids attenuated cell death induced by *COPB2* knockdown, whereas cell death induced by *BCLXL* depletion was unaffected (Fig. 3e). Knockdown of the alternative splicing regulator *PTBP1* also results in SASP inhibition, without affecting other senescence phenotypes^32^ (Supplementary Fig. 5e–g). Depletion of *PTBP1* in senescent cells also prevented cell death induced by *COPB2* knockdown (Supplementary Fig. 5h).

We hypothesized that aberrant accumulation of the SASP (and other misfolded proteins) on senescent cells could trigger an unfolded protein response (UPR), which may contribute to the senolytic effects associated with *COPB2* knockdown. The UPR senses the aberrant accumulation of proteins in the ER, and activates the transcription factors CHOP, ATF6 and XBP1^33^. GSEA showed that signatures associated with activation of the UPR response or some of their key mediators (such as PERK and IRE1α) were upregulated in cells undergoing OIS or bleomycin-induced senescence following *COPB2* knockdown (Fig. 3f and Supplementary Fig. 6). To further investigate whether COPI inhibition selectively activates the UPR in senescent cells, we assessed the frequency of cells with nuclear accumulation of these transcription factors. *COPB2* knockdown selectively activated the UPR in cells undergoing OIS, but not in non-senescent cells (Fig. 3g,h and Extended Data Fig. 3e). Overall, these data suggest that the senolytic effects associated with *COPB2* knockdown may relate to the selective activation of the UPR response in senescent cells.

### COPI inhibition triggers UPR and dysfunctional autophagy

Next, we explored the mechanism behind the senolytic effect associated with COPI inhibition using GBF1 inhibitors, which allow for an acute and sustained inhibition of the pathway by impeding COPI complex formation.

**Fig. 4:**
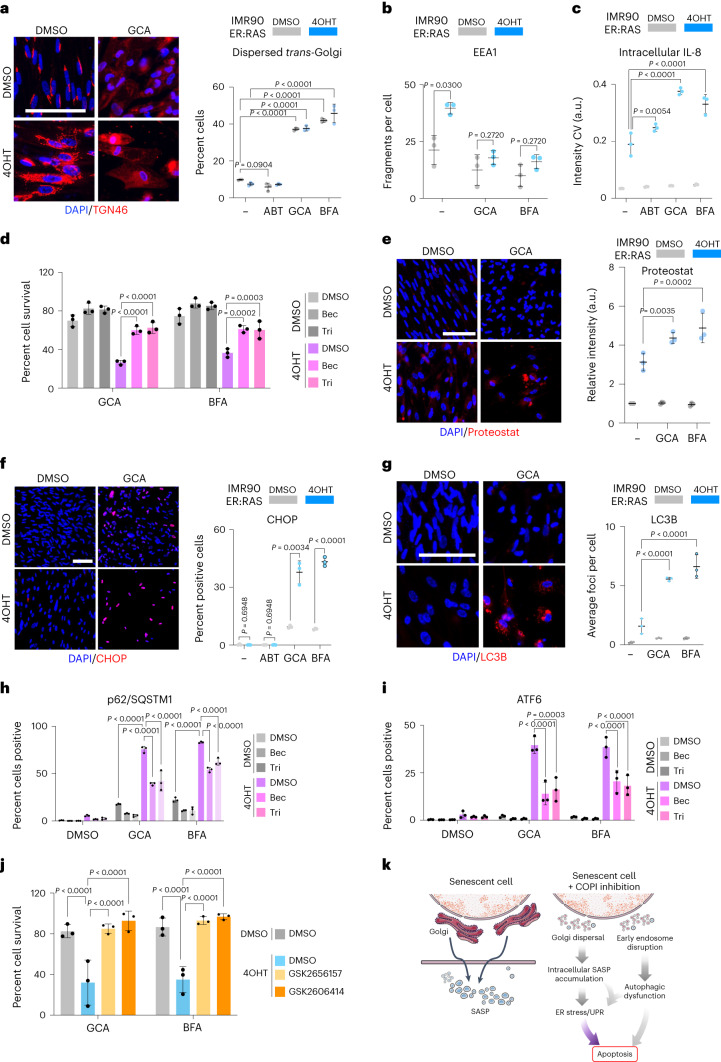
COPI inhibitors cause Golgi disruption, trigger UPR and result in autophagy defects. **a**–**c**, Percentage fragmented *trans*-Golgi (**a**, right), early endosome numbers per cell (**b**) and intracellular levels of IL-8 (**c**) by IF in senescent (4OHT) and control (DMSO) IMR90 ER:RAS cells after treatment for 48 h with 1.25 µM GCA, 150 nM BFA or 1 µM ABT-263 (*n* = 3). Representative IF images are shown in **a** (left) and Extended Data Fig. 4a (for **b**) and Supplementary Fig. 7a for **c**. Scale bar, 100 μm. **d**, Percentage cell survival of senescent (4OHT) and control (DMSO) IMR90 ER:RAS cells treated with either 1.25 µM GCA or 150 nM BFA (*n* = 3) following glucocorticoid (10 µM Bec, beclomethasone; 10 µM Tri, triamcinolone) pretreatment four days after senescence induction. **e**, Right: relative Proteostat signal intensity in senescent (4OHT) or control (DMSO) IMR90 ER:RAS cells after 48 h treatment with 1.25 µM GCA or 150 nM BFA (*n* = 3). Left: representative IF images. Scale bar, 100 µm. **f**,**g**, Right: percentage positive cells for nuclear CHOP (**f**) or LC3B foci number (**g**) by IF, 48 h after either control (DMSO) or senescent (4OHT) cells were treated with either 1 µM ABT-263, 1.25 µM GCA or 150 nM BFA (*n* = 3). Left: representative IF images for CHOP (**f**) and L3CB (**g**). Scale bars, 100 µm. **h**,**i**, Percentage p62/SQSTM1 (**h**) and ATF6 (**i**) positive cells by IF in senescent (4OHT) and control (DMSO) IMR90 ER:RAS cells treated with either 1 µM ABT-263, 1.25 µM GCA or 150 nM BFA following glucocorticoid pretreatment as in **d**. IF staining was carried out 48 h post senolytic drug addition (*n* = 3). **j**, Survival of control (DMSO) or OIS (4OHT) cells pre-treated with 1 µM GSK2656157 or 1 µM GSK2606414 before a 48 h treatment with GCA or BFA at day 7 post senescence induction (*n* = 3). **k**, Scheme summarizing how COPI inhibition induces the death of senescent cells. All data throughout the figure are presented as mean ± s.d. *n* represents independent experiments throughout the figure. Two-way ANOVA was performed for statistical analysis in **a**, **c**–**e**, **g**, **h**, **i** and **j**. Unpaired, two-tailed Student’s *t*-test was used for statistical analysis of the data in **b** and **f**. Source numerical data are available as source data. Source data

**Extended Data Fig. 4:**
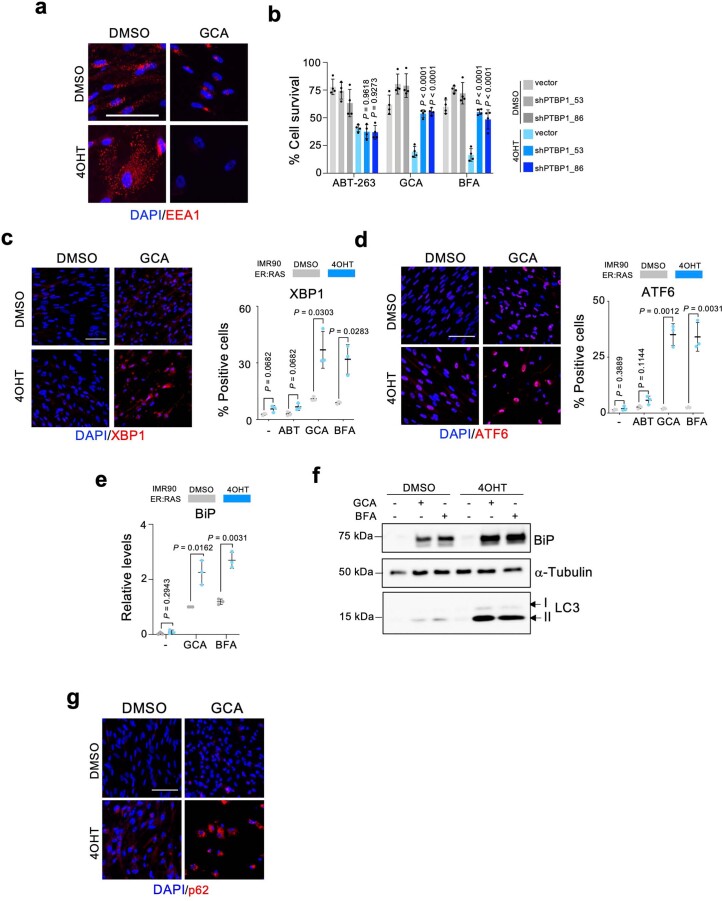
Effects of COPI inhibition in the unfolded protein response and autophagy. **a**, Representative IF images of EEA1 staining in control (DMSO) and senescent (4OHT) IMR90 ER:RAS treated with either DMSO, 1.25 µM of golgicide A (GCA) or brefeldin A (BFA) for 48 h, 7 days after senescence induction. (n = 3) (Quantification provided in Fig. 4b) Scale bar, 100 µm. **b**, Percentage cell survival of senescent (4OHT) and control (DMSO) IMR90 ER:RAS cells transduced with indicated shRNA or vector and treated with either ABT-263, 1.25 µM GCA or 150 nM BFA for 72 h, 7 days after senescence induction (n = 4). Ordinary Two-way ANOVA with comparison is to the corresponding IMR90 ER: RAS vector +4OHT condition. **c-d**, Percentage cells positive for nuclear XBP1 (c) and ATF6 (d) by IF, 48 hours after either control (DMSO) or oncogene-induced senescent (4OHT) cells were treated with either 1 µM ABT-263, 1.25 µM GCA or 150 nM BFA (n = 3). Representative IF images (left, c and d). Scale Bar, 100 µm. Significance was calculated using unpaired, two-tailed, Student’s t-test. **e-f**, Quantification (e) of the level of BiP protein (relative to α-tubulin) as assessed by western blot of either control (DMSO) or senescent (4OHT) 48 h after treatment with either 1.25 µM GCA or 150 nM BFA (n = 3) and 7 days after senescence induction. Unpaired, two-tailed, Student’s t-test. Representative immunoblot (out of three independent experiments) (f) shown for BiP, α-tubulin, and LC3 is shown. Immunoblot of α-tubulin is included as a loading control. **g**, Representative IF images for p62/SQSTM1 immunostaining are shown for control (DMSO) and senescent (4OHT) IMR90 ER: RAS treated with 1.25 µM GCA for 48 h 7 days post senescence induction (Quantification provided in Fig. 4h) (n = 3). Scale bar, 100 µm. Data throughout figure represented as mean ± SD where applicable. N represents independent experiments throughout figure. Source numerical data and unprocessed blots are available in source data. Source data

Treatment with GBF1 inhibitors GCA or BFA caused Golgi dispersal in both senescent and non-senescent cells (Fig. 4a) and prevented the expansion of early endosomes in senescent cells (Fig. 4b and Extended Data Fig. 4a). Moreover, treatment with GBF1 inhibitors caused the intracellular accumulation of SASP components, including IL-8 (Fig. 4c and Supplementary Fig. 7a), VEGF, GM-CSF and BMP2/4 in senescent cells (Supplementary Fig. 7b). This intracellular accumulation was not due to increased SASP transcription, as treatment with GBF1 inhibitors caused a decrease in the mRNA levels of SASP components (Supplementary Fig. 7c), probably due to a compensatory reduction in transcription following the UPR^34^. Secretion of multiple SASP components was also significantly reduced in senescent cells treated with GBF1 inhibitors (Supplementary Fig. 7d).

As we observed upon *COPB2* knockdown in senescent cells, inhibition of SASP production prevented the senolytic effects of GBF1 inhibitors (Fig. 4d and Extended Data Fig. 4b). We reasoned that intracellular accumulation of SASP components might reflect a wider trend for senescent cells to accumulate aberrant proteins upon COPI inhibition. To explore this possibility, we measured misfolded and aggregated proteins using Proteostat^35^. Proteostat staining showed higher levels of misfolded/aggregated proteins in senescent cells, an increase that was exacerbated upon treatment with GBF1 inhibitors (Fig. 4e).

GBF1 inhibitors also triggered selective UPR activation on cells undergoing OIS, as shown by the increased expression of CHOP, XBP1, ATF6 and BiP (Fig. 4f and Extended Data Fig. 4c–f). Importantly, UPR activation was also observed upon COPI inhibition in cells undergoing bleomycin-induced senescence (Supplementary Fig. 8), even though the SASP in cells undergoing bleomycin-induced senescence is not induced to the same extent as in cells undergoing OIS (Supplementary Fig. 9a). The effects of GBF1 inhibitors on Golgi dispersal are known to be reversible^36^. Consistent with this reversibility, the senolytic effects of GBF1 inhibitors on IMR90 cells undergoing bleomycin-induced senescence could be prevented if the drugs were removed 24 h, but not 48 h, post treatment (Supplementary Fig. 9b), whereas treatment with GBF1 inhibitors for 24 h was sufficient to trigger the death of cells undergoing OIS (Supplementary Fig. 9c), potentially due to its greater SASP response.

COPI inhibition can result in an accumulation of non-degradative autophagosomes and impaired autophagy^29^. COPI inhibition resulted in the accumulation of LC3 and p62, suggesting a block in normal autophagic flux (Fig. 4g and Extended Data Fig. 4f,g). Glucocorticoid treatment, which inhibits SASP production, also reduced p62 accumulation in senescent cells treated with GBF1 inhibitors (Fig. 4h), suggesting that the autophagy defect may be driven in part by overwhelming of the autophagy machinery by the accumulation of aberrant proteins. Moreover, glucocorticoids also prevented UPR, as suggested by the lower frequency of nuclear ATF6 in senescent cells treated with GBF1 inhibitors (Fig. 4i). Finally, to assess whether UPR activation mediates the senolytic effects of GBF1 inhibitors, we inhibited the UPR effector kinase PERK. Two PERK inhibitors (GSK2656157 and GSK2606414) did not affect senescence (Supplementary Fig. 10), but prevented senolysis by GCA and BFA (Fig. 4j). In summary, COPI inhibition causes Golgi dispersal, accumulation of aberrant proteins, early endosome disruption and dysfunctional autophagy in senescent cells, which results in proteotoxic responses and causes their selective killing (Fig. 4k).

### Therapeutic benefits associated with COPI inhibition

**Fig. 5:**
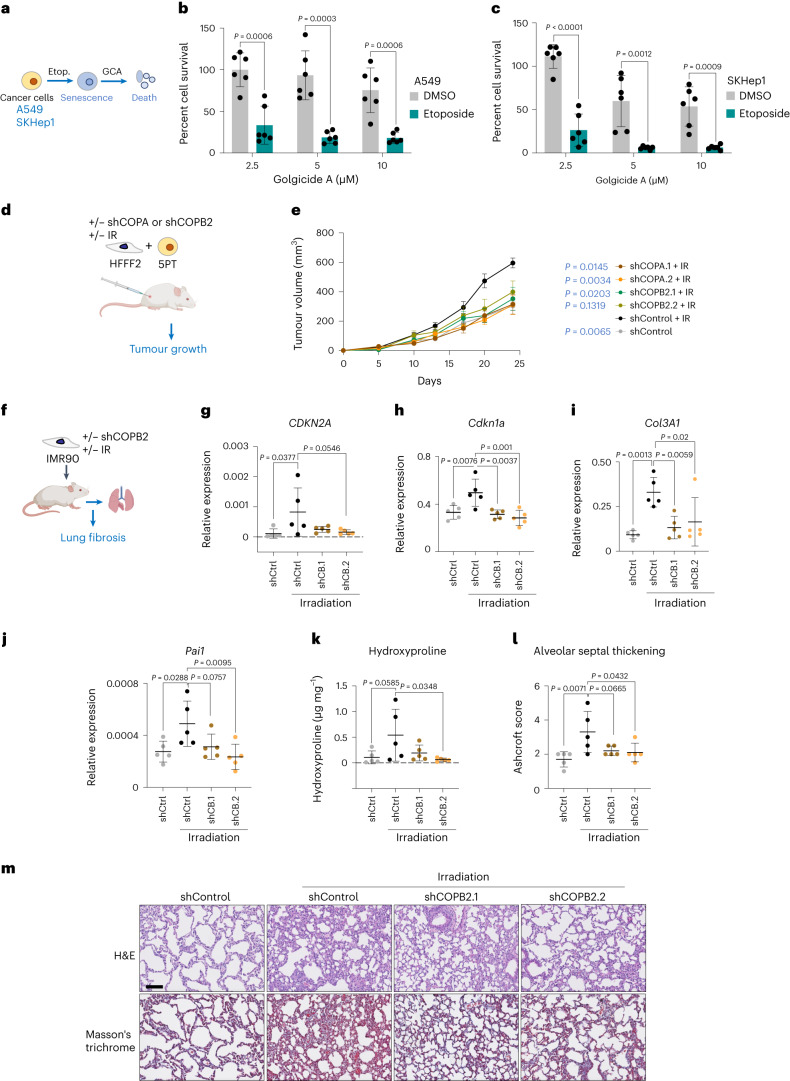
Therapeutic benefits of inhibiting the COPI pathway. **a**, Experimental design for the sequential treatment of cancer cells with chemotherapy and GCA. Etop., etoposide. **b**,**c**, Quantification of cell survival of A549 cells (**b**) or SKHep1 cells (**c**) after treatment with the indicated drug combinations (*n* = 6). Unpaired, two-tailed, Student’s *t*-test. Data are presented as mean ± s.d. **d**, Experimental design of tumour growth in mice co-injected with 5PT squamous cancer cells and HFFF2 fibroblasts. IR, irradiation. **e**, Tumour growth curves showing the tumour volume monitored over time. Data are presented as mean ± s.e.m. for all mice in each group (*n* = 7 mice per group, shCOPB2.1 + IR, *n* = 6 mice). Repeated Measure (RM) two-way ANOVA with Greenhouse–Geisser correction and Dunnett’s correction was used for statistical analysis of the day-20 timepoint relative to shControl+IR. The AUC analysis for data pooled from two experiments is shown in Extended Data Fig. 5d. All comparisons are to shControl+IR. **f**, Experimental design of the mouse model of lung fibrosis by intratracheal instillation of human senescent lung fibroblasts into nude mice. All analyses were performed three weeks after cell delivery (except those in Extended Data Fig. 5h, which were performed 48 h post-instillation). **g**–**j**, Relative expression of the mRNAs coding for human *CDKN2A* (**g**), or mouse *Cdkn1a* (**h**), *Col3a1* (**i**) and *Pai1* (**j**) in lung samples from the experiment described in **f** (*n* = 5 mice per group). Statistical analysis was performed using ordinary one-way ANOVA. Data are presented as mean ± s.d. **k**, Lung hydroxyproline content in samples from mice of the experiment described in **f** (*n* = 5 mice per group). Ordinary one-way ANOVA. Data are presented as mean ± s.d. **l**, Ashcroft scoring for alveolar septal thickening in sections from lungs of mice grafted with IMR90 cells treated as indicated (*n* = 5 mice per group). Ordinary one-way ANOVA. Data are presented as mean ± s.d. **m**, Representative images of lung sections stained with haematoxylin and eosin (H&E, top) and Masson’s trichrome (bottom) from mice of the experiment described in **f**. Scale bar, 100 µm. *n* represents independent experiments or mice throughout the figure. Source numerical data are available as source data. Source data

**Extended Data Fig. 5:**
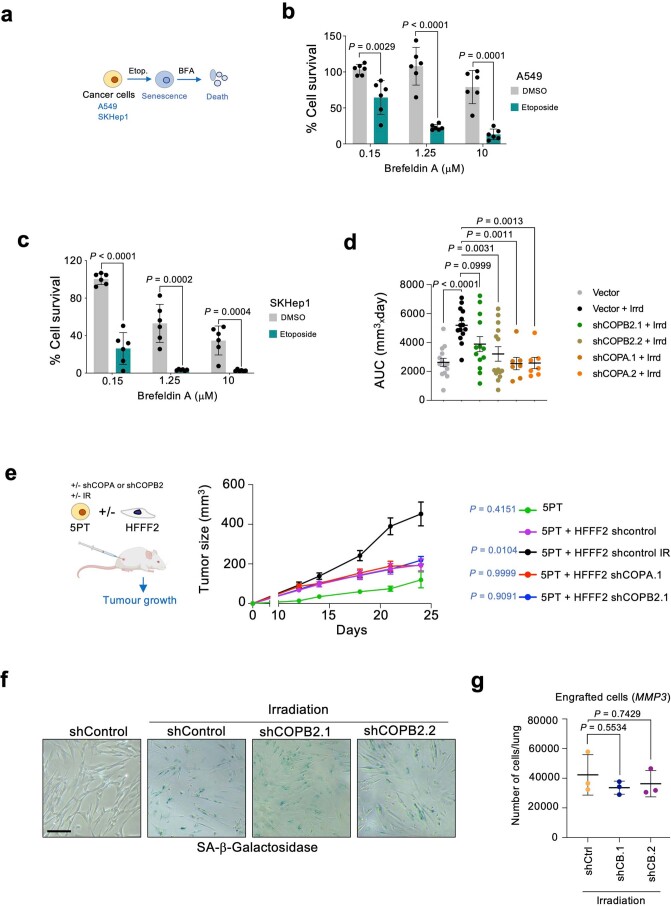
Therapeutic benefits of inhibiting the COPI pathway. **a**, Experimental design for the sequential treatment of cancer cells with chemotherapy and brefeldin A (BFA). **b-c**, Quantification of cell survival of A549 cells (b, n = 6) or SKHep1 cells (c, n = 6) after treatment with the indicated drug combinations. Unpaired, two-tailed, Student’s t-test. Data represented as mean ± SD. **d**, Area under the curve (AUC) analysis for tumour volume measured over time in two independent experiments (Experiment A and B; see Fig. 5e for the tumour growth curves of Experiment A and Supplemental Figure 12f for Experiment B). Data represented as mean ± SEM (n = 14 mice per group, Vector, Vector + Irrd and shCOPB2.2 + Irrd; n = 13 mice, shCOPB2.1 + Irrd; n = 7 mice per group shCOPA.1 + Irrd and shCOPA.2 + Irrd). Ordinary two-way ANOVA was used for the statistical comparison of groups to Vector + Irrd. **e**, Experimental design (left) of tumour growth in NSG cancer model with 5PT squamous cancer cells co-injected with HFFF2 fibroblasts (Right) Tumour growth curves showing the tumour volume monitored over time (IR=irradiation). Data represented as mean ± SEM (n = 8 mice per group, 5PT n = 6 mice). RM Two-way ANOVA with Geisser Greenhouse correction and Dunnet’s correction used for comparisons to 5PT + HFFF2 shControl used for the statistical test. P values are shown for the final time point. **f**, Representative images (of three independent experiments) of SA-β-galactosidase staining in irradiated IMR90s transduced with shRNAs against COPB2 with positive cells staining blue. Scale bar, 200 µm. **g**, Quantification of the initial engraftment of human fibroblasts in the lungs of nude mice measured 48 hours after the instillation of 5×10^5^ of the indicated cells. Engraftment was assessed by the expression levels of mRNAs coding for human MMP3. Ordinary one-way ANOVA and (shCntl, n = 3 mice, shCOPB2.1, n = 3 mice, shCOPB2.2, n = 3 mice). Data represented as mean ± SD. N in figure (b, c, e, g) represents independent experiments or mice. N in figure (d) represents mice from two pooled experiments (Experiment A and Experiment B) Source numerical data are available in source data. Source data

To determine whether chemotherapy renders cancer cells sensitive to GBF1 inhibitors, we induced senescence by treatment with etoposide (Supplementary Fig. 11) and subsequently treated them with GCA or BFA (Fig. 5a and Extended Data Fig. 5a). Treatment with GCA (Fig. 5b,c) or BFA (Extended Data Fig. 5b,c) selectively killed cancer cells that were previously rendered senescent by etoposide.

The SASP can enhance the proliferative potential of cancer cells and promote tumour progression^37^. To investigate whether COPI inhibition in senescent cells compromises their ability to promote tumourigenesis, we used an experimental xenograft mouse model that monitors the effect of senescent fibroblasts on tumour growth^32,38^. In addition, we took advantage of HFFF2 fibroblasts with doxycycline-inducible expression of shRNAs targeting either *COPA* or *COPB2* (Supplementary Fig. 12a–e). We subcutaneously co-injected squamous cell carcinoma 5PT cells^39^ with normal or senescent (irradiated) fibroblasts into immunodeficient mice (Fig. 5d) and confirmed that senescent fibroblasts enhanced tumour growth (Fig. 5e and Extended Data Fig. 5d). Depletion of *COPA* or *COPB2*, using two independent shRNAs targeting each gene, impaired the ability of irradiated, senescent fibroblasts to promote the growth of 5PT tumour cells in this setting (Fig. 5e, Supplementary Fig. 12f and Extended Data Fig. 5d).

We next examined a model of lung fibrosis^16,40^. In this model, normal or senescent (gamma-irradiated; Extended Data Fig. 5g) human IMR90 fibroblasts, bearing doxycycline-inducible shControl or shCOPB2, were transplanted into the lung of immunodeficient mice (Fig. 5f). Mice were treated with doxycycline to induce shRNA expression. We measured, by quantitative polymerase chain reaction (qPCR), the human-specific gene (*MMP3*) to check that the different cells were engrafted similarly (Extended Data Fig. 5h). Three weeks after intratracheal instillation, we assessed the expression of *CDKN2A* (the human gene encoding p16^INK4a^) to detect senescent human fibroblasts in the lung. *CDKN2A* expression was lower in the lungs of mice transplanted with senescent fibroblasts expressing shCOPB2, suggesting that *COPB2* depletion killed the transplanted senescent cells (Fig. 5g).

Interestingly, we observed increased expression of murine *Cdkn1a*, *Col3A1* and *Pai1* (Fig. 5h–j) in the lungs of mice transplanted with senescent fibroblasts that expressed shControl but not shCOPB2. These data suggest that senescent fibroblasts trigger senescence and lung fibrosis non-autonomously and that this consequence is attenuated by eliminating senescent cells via COPI inhibition. To assess lung fibrosis, we measured hydroxyproline levels in the lung. This analysis confirmed that the injection of senescent cells, but not senescent shCOPB2 cells, increased lung fibrosis (Fig. 5k). We stained the lungs with Masson’s trichome to further monitor fibrosis and observed increased fibrosis in the lungs from mice injected with senescent fibroblasts versus control fibroblasts, while fibrosis was reduced in the lungs of mice injected with senescent fibroblasts expressing shCOPB2 (Fig. 5l,m) as graded by Ashcroft scoring. In combination, these data suggest that inhibiting COPI can ameliorate the consequences associated with the presence of senescent cells in cancer and fibrosis.

### Targeting NMTs phenocopies COPI inhibition

The poor pharmacological properties of existing drugs targeting the COPI pathway (such as BFA) have hampered their use in the clinic^41^. Diverse post-translational modifications regulate the COPI pathway^42^. Recently, global analysis of *N*-myristoylated proteins identified ARF GTPase family members^43,44^, suggesting that impairment of COPI function may be a key effect of pharmacologically inhibiting *N*-myristoylation.

**Fig. 6:**
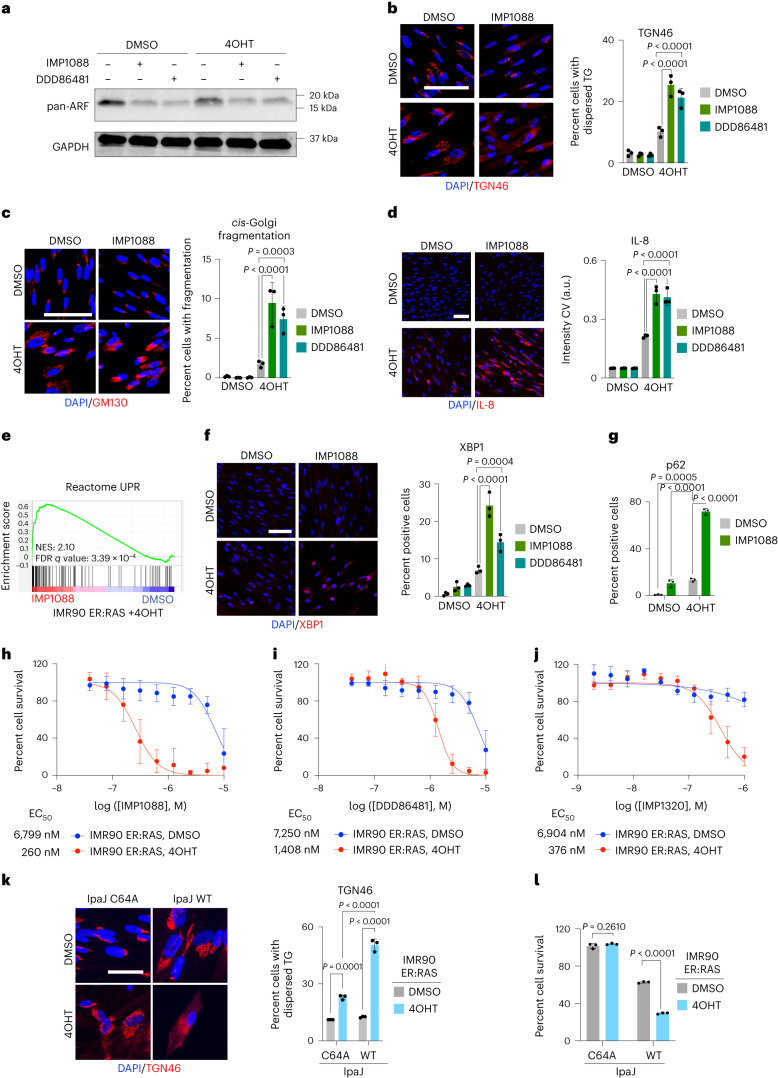
NMTi phenocopy COPI inhibition and are senolytic. **a**, Western blots of ARF GTPases for control (DMSO) or senescent (4OHT) IMR90 ER:RAS cells 72 h after treatment with 300 nM IMP1088 or 1.5 µM DDD86481, seven days after senescence induction. An immunoblot of GAPDH is included as a loading control. Representative immunoblots from three independent experiments are shown. **b**–**d**, Right: quantification of IF staining for *trans*-Golgi (TG) dispersal (TGN46, **b**), *cis*-Golgi dispersal (GM130, **c**) and intracellular levels of IL-8 (**d**) in control (DMSO) or senescent (4OHT) IMR90 ER:RAS cells five days after treatment with 300 nM IMP1088 or 1.5 µM DDD86481, seven days after senescence (*n* = 3). Left: representative IF images. Scale bars, 100 µm. **e**, GSEA plot of the UPR gene signature in IMR90 ER:RAS treated with the NMTi IMP1088. **f**, Right: quantification of XBP1 IF staining in control (DMSO) or senescent (4OHT) IMR90 ER:RAS cells treated for five days with 300 nM IMP1088 or 1.5 µM DDD86481 seven days after senescence induction (*n* = 3). Left: representative IF images. Scale bar, 100 µm. **g**, Quantification of IF staining for p62/SQSTM1. Control (DMSO) or senescent (4OHT) IMR90 ER:RAS cells were treated with 300 nM IMP1088 or 1.5 µM DDD86481 seven days after senescence induction for five days (*n* = 3). **h**–**j**, Dose–response curves of control (DMSO) or senescent (4OHT) IMR90 ER:RAS cells treated for seven days with NMT inhibitors, seven days after senescence induction, with IMP1088 (**h**, *n* = 9), DDD86481 (**i**, *n* = 5) and IMP1320 (**j**, *n* = 4). **k**, Right: quantification of dispersed *trans*-Golgi in C64A or WT IpaJ transduced control (DMSO) or senescent (4OHT) IMR90 ER:RAS cells (*n* = 3) seven days post senescence induction. Left: representative IF images. Scale bar, 50 μm. **l**, Percentage survival of C46A or WT IpaJ transduced control (DMSO) or senescent (4OHT) IMR90 ER:RAS cells seven days post senescence induction. Survival is measured relative to vector-transduced cells. Unpaired two-tailed Student’s *t*-test (*n* = 3). Data are presented as mean ± s.d. Statistical analysis was performed throughout the figure by ordinary two-way ANOVA unless otherwise specified. *n* represents independent experiments throughout. Source numerical data and unprocessed blots are available as source data. Source data

**Extended Data Fig. 6:**
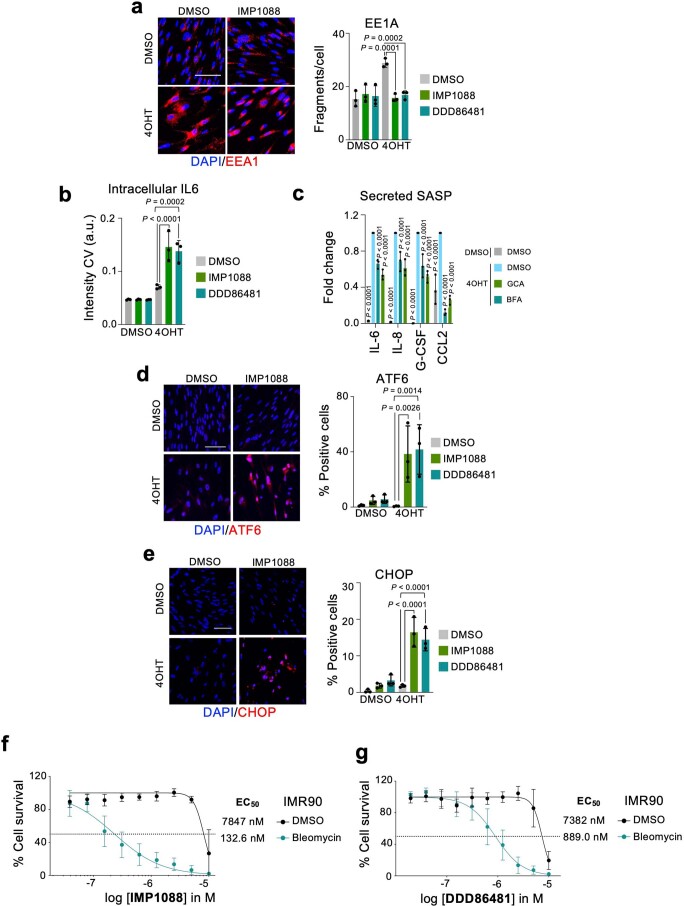
NMT inhibitors phenocopy the effects of inhibiting COPI in senescent cells. **a**, Quantification of IF staining for EEA1 vesicles in control (DMSO) or senescent (4OHT) IMR90 ER:RAS cells were treated with 300 nM IMP1088 or 1.5 µM DDD86481 for 5 days, 7 days after senescence induction (n = 3). Ordinary Two-way ANOVA. Representative IF image (left). Scale bar, 100 µm. **b**, Quantification of intracellular levels of IL6 in control (DMSO) or senescent (4OHT) IMR90 ER:RAS cells treated with 300 nM IMP1088 or 1.5 µM DDD86481 for 5 days, 7 days after senescence induction (n = 3). Ordinary Two-way ANOVA. Representative IF images (left). Scale bar, 100 µm. **c**, Relative fold change of IL6, IL8, G-CSF, and CCL2 in the supernatant of (DMSO) or day 7 senescent cells (4OHT + DMSO) treated with NMTi (4OHT + IMP1088/DDD8641) for 5 days. Concentration determined by ELISA normalized to cell counts and shown relative to senescent cells (4OHT + DMSO) (n = 3). Comparisons are to senescent cells (4OHT + DMSO). Significance was calculated using ordinary two-way ANOVA, Dunnett’s correction. **d-e**, Percentage of cells positive nuclear ATF6 (d) and nuclear CHOP (e) by IF in control (DMSO) or senescent (4OHT) IMR90 ER:RAS cells treated with 300 nM IMP1088 or 1.5 µM DDD86481 for 5 days, 7 days after senescence induction (n = 3). Ordinary two-way ANOVA, Dunnett’s correction. Representative IF images are shown (left). Scale bar, 100 µm. **f-g**, Dose-response curves for senolytic effect of NMT inhibitors in bleomycin-induced senescence. IMP1088 (f, n = 7), DDD86481 (g, n = 5). EC_50_, half-maximal effective concentration. Control (DMSO) or senescent (Bleomycin) IMR90 was treated with inhibitors 7 days after senescence induction and fixed for assessing survival 7 days after treatment. Data is represented as mean ± SD throughout the figure. N throughout figure represents independent experiments. Source numerical data are available in source data. Source data

Treatment of control and senescent IMR90 ER:RAS cells with two *N*-myristoyltransferase inhibitors (NMTi: IMP1088 and DDD86481)^45^ resulted in lower levels of ARF GTPases, as unveiled using a pan-ARF antibody (recognizing ARF1, ARF3, ARF5 and ARF6; Fig. 6a). This decreased expression probably reflects the increased proteasomal degradation that may be observed for proteins failing to undergo *N*-myristoylation^46^. Importantly, senescent cells treated with NMT inhibitors (IMP1088 and DD86481) displayed increased Golgi dispersal (Fig. 6b,c), endosomal disruption (Extended Data Fig. 6a) and intracellular accumulation of IL-8 and IL-6 (Fig. 6d and Extended Data Fig. 6b). NMTi did not affect SASP transcription (Supplementary Fig. 13a), but resulted in reduced secretion of multiple SASP components (Extended Data Fig. 6c). GSEA showed an enrichment of signatures related to UPR activation in senescent cells treated with NMTi (Fig. 6e and Supplementary Fig. 13b,c). Moreover, senescent cells treated with NMTi activated UPR (Fig. 6f and Extended Data Fig. 6d,e, as assessed by the accumulation of XBP1, ATF6 and CHOP) and displayed dysfunctional autophagy (Fig. 6g). This is consistent with a previous report showing that NMTi induce ER stress in cancer cells^47^. Treatment with three different NMTi (IMP1088, DD86481 and IMP1320)^45^ selectively killed cells undergoing OIS (Fig. 6h–j) by apoptosis (Supplementary Fig. 13d). NMTi also killed cells undergoing bleomycin-induced senescence (Extended Data Fig. 6f,g).

The *Shigella* virulence factor IpaJ can induce the proteolytic cleavage of the *N*-myristoylated N-terminal glycine of ARF1^48^. Importantly, substrate recognition makes IpaJ cleavage of *N*-myristoyl modifications selective for a limited number of proteins, including ARF1^49^. We expressed wild-type IpaJ (WT) or an inactive C64A mutant on IMR90 cells and, taking advantage of ω-alkynyl myristate (YnMyr) labelling^43,50^, we confirmed that while NMTi inhibited *N*-myristoylation of proteins with YnMyr, that was not the case when WT IpaJ was expressed (Supplementary Fig. 14a). Indeed, western quantifications showed that, in contrast with NMTi, which reduced the expression of multiple *N*-myristoylated proteins, the expression of WT IpaJ reduced the expression of ARF1 but not of other *N*-myristoylated proteins (Supplementary Fig. 14b–e). IpaJ WT, but not the inactive IpaJ C64A mutant, resulted in increased Golgi dispersal in senescent cells (Fig. 6k) and was also senolytic (Fig. 6l). These experiments further suggest that reducing ARF1 *N*-myristoylation disrupts the COPI pathway. Overall, these results demonstrate that NMT inhibitors behave as senolytic agents, and phenocopy the effects of COPI inhibition.

### NMTi are senolytic in different cancer models

**Fig. 7:**
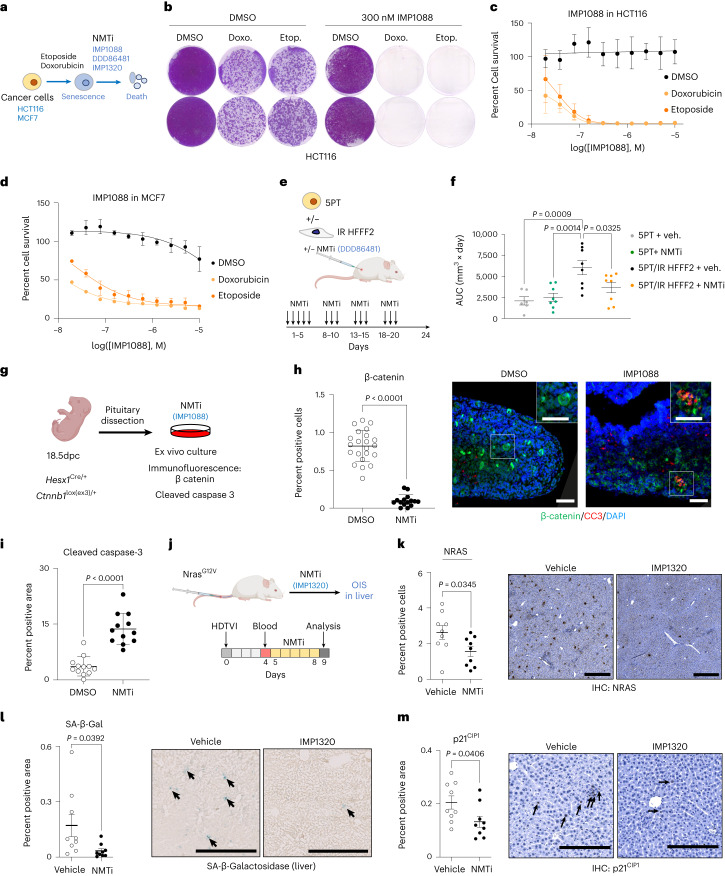
NMTi target senescent cells in cancer models. **a**, Experimental design for the sequential treatment of cancer cells with chemotherapy and NMTi. **b**, Crystal violet staining of control (DMSO) and senescent (treated with doxorubicin or etoposide) HCT116 cells treated with 300 nM IMP1088 for seven days, seven days after senescence induction. The images show the results of two independent experiments. **c**,**d**, Dose–response curves in HCT116 (**c**, *n* = 4) or MCF7 (**d**, *n* = 3 for DMSO and etoposide and *n* = 2 for doxorubicin) cells treated with either doxorubicin or etoposide and treated with IMP1088 seven days post senescence induction. Data are presented as mean ± s.d. **e**, Experimental design for **f**. **f**, AUC analysis for tumour volume measured over time. Data are presented as mean ± s.e.m. (*n* = 6 mice, 5PT+veh; *n* = 8, other groups; also Extended Data Fig. 7e). Ordinary one-way ANOVA. **g**, Tumoural pituitaries from 18.5dpc *Hesx1*^Cre/*+*^;*Ctnnb1*^lox(ex3)/+^ embryos were cultured in the presence of NMTi (600 nM IMP1088) or vehicle (DMSO) and processed for histological analysis after 72 h. **h**, Left: quantification of β-catenin-accumulating cells after NMTi treatment. Right: images with representative IF staining with β-catenin and cleaved caspase-3 (CC3). Main scale bars, 50 μm; inset scale bars, 40 μm. Data are presented as mean ± s.d. *n* represents the total number of histological sections analysed (*n* = 22, DMSO; *n* = 15, NMTi). Unpaired, two-tailed Student’s *t*-test. **i**, Quantification of CC3-positive area (percent of the pituitary surface) after NMTi treatment (*n* = 12 pituitary sections per group). Data are presented as mean ± s.d. Unpaired, two-tailed, Student’s *t*-test. **j**, Experimental design of the liver oncogene-induced senescence experiment. **k**–**m**, Left: quantification of Nras-positive cells (**k**), SA-β-Gal staining (**l**) and p21^CIP1^ staining by immunohistochemistry (IHC) (**m**) in the liver of mice treated with vehicle or IMP1320 (*n* = 9 mice per group). Data are presented as mean ± s.e.m. Unpaired, two-tailed, Student’s *t*-test. Right: representative IHC images (**k**–**m**). Arrows indicate examples of SA-β-Gal-positive cells. Scale bars, 100 μm. *n* represents independent experiments or mice unless otherwise specified. Source numerical data are available as source data. Source data

**Extended Data Fig. 7:**
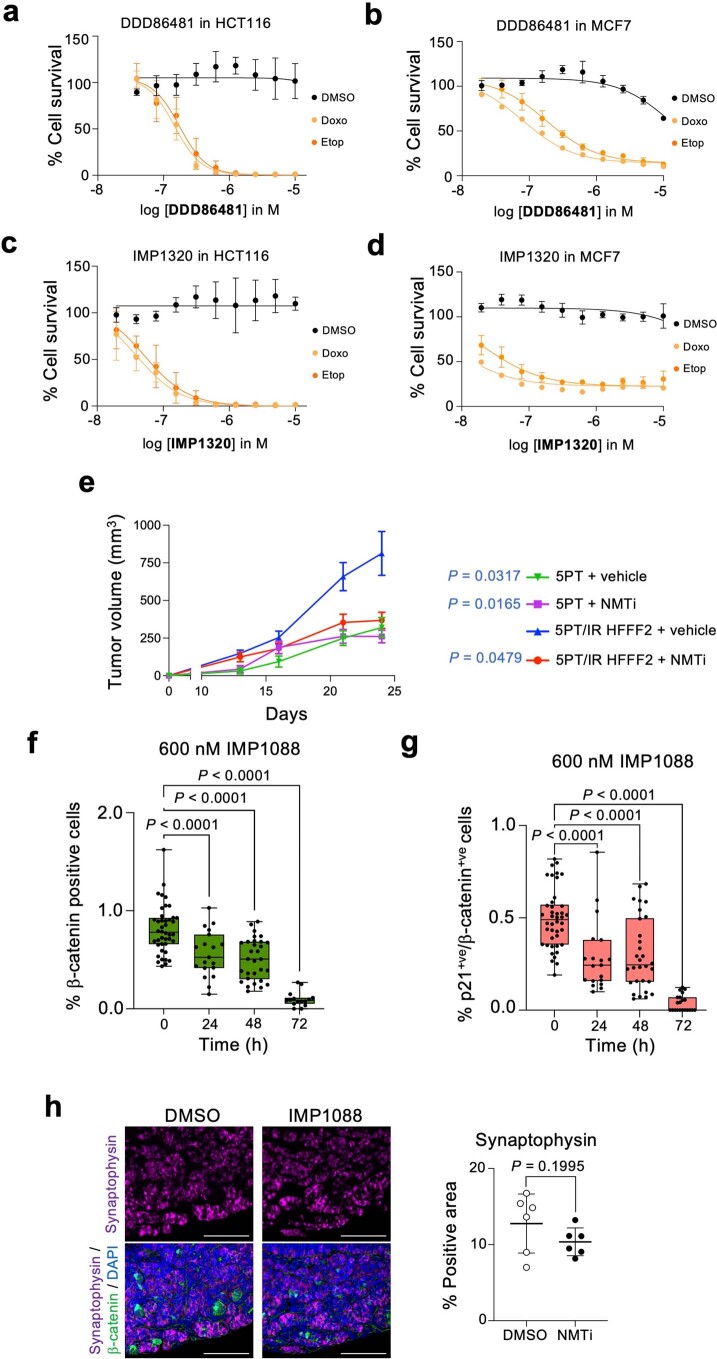
NMT inhibitors target senescent cells in cancer models. **a-b**, Dose-response curves for assessing the senolytic activity of DDD86481 treated HCT116 (a, n = 4 for each condition) or MCF7 (b, n = 3 for DMSO, n = 3 for etoposide, n = 2 for doxorubicin) cells induced to senesce with either doxorubicin or etoposide. Data represented as mean ± SD **c-d**, Dose-response curves for assessing the senolytic activity of IMP1320 treated HCT116 (c, n = 4 for each condition) or MCF7 (d, n = 3 for DMSO, n = 3 for etoposide, n = 2 for doxorubicin) cells induced to senesce with either doxorubicin or etoposide treatment. EC_50_, half-maximal effective concentration. Data represented as mean ± SD. **e**, Tumour growth curves showing the tumour volume monitored over time (IR=irradiation). Data represented as mean ± SEM (n = 6 mice 5PT + Veh, n = 8 mice per group all other groups; see the Fig. 7f for the relative AUC analysis. RM Two-way ANOVA with Geisser Greenhouse correction and Dunnet’s correction used for to 5PT/IR HFFF2 + vehicle used for the statistical test. P values are shown as the final timepoint. **f-g**, Quantification of β-catenin positive (f) and p21^Cip1^ positive/ β-catenin positive (g) cells in the pituitary gland at 0, 24, 48, and 72 h after treatment with 600 nM IMP1088 *ex vivo*. Data is box and whisker plot; box, 25^th^ to 75^th^ percentile; whisker minimum to maximum with all points shown; n represents the number of sections. (f, 0 h, n = 42; 24 h, n = 19; 48 h, n = 30; 72 h, n = 15) (g, 0 h, n = 42; 24 h, n = 19; 48 h, n = 30; 72 h, n = 21) Ordinary One-way ANOVA with Dunnett’s correction. **h**, Synaptophysin is a marker of the normal hormone-producing cells in the pituitary gland, quantification of synaptophysin-positive area (purple; % of the pituitary surface) after NMTi treatment highlights no significant effect of the treatment on normal cells. The left panel shows representative images (n = 6 sections for all groups). Unpaired, two-tailed, Student’s t-test. Scale bar, 50 µm. N in figure (a-e) represent independent experiments or mice. N in figure (f and g, h) represents histological sections. Source numerical data are available in source data. Source data

To understand whether NMTi could act as a senolytic in a ‘one–two punch’ strategy, we induced senescence in cancer cells with etoposide or doxorubicin (Supplementary Fig. 15) and subsequently treated them with NMTi (Fig. 7a). Treatment with IMP1088 (Fig. 7b–d), DDD86481 (Extended Data Fig. 7a,b) or IMP1320 (Extended Data Fig. 7c,d) selectively killed senescent cancer cells.

NMTi are tolerated at moderate doses both in mice^51^ and humans^52^. To confirm that our NMTi treatment regimens did not cause toxicities, we administered either DDD86481 or IMP1320 to mice and monitored several markers in blood without detecting any significant metabolic alterations (Supplementary Fig. 16a,b) or any changes in glucose, insulin levels or cell-type composition in the blood (Supplementary Fig. 16c,d).

To understand the potential benefit of NMTi in targeting senescent cells in the tumour microenvironment, we took advantage of a previously described experimental model that combines the xenograft of senescent fibroblasts and 5PT cancer cells^32,38^ (Fig. 5d,e). We co-injected 5PT cells alone or together with senescent, irradiated HFFF2 cells and assessed the effect of the NMTi DDD86481 on tumour growth (Fig. 7e). Although NMTi did not interfere with the growth of a xenograft caused by 5PT cells implanted alone, it abrogated the promotion of tumour growth caused by co-injecting senescent fibroblasts (Fig. 7f and Extended Data Fig. 7e).

To understand whether NMTi could also eliminate preneoplastic senescent cells in a tissue context, we used a model of adamantinomatous craniopharyngioma (ACP), a Wingless-related integration site (WNT) pathway-driven clinically relevant pituitary paediatric tumour in which clusters of β-catenin-positive preneoplastic senescent cells promote tumourigenesis in a paracrine manner^53^. We have previously used ex vivo pituitary cultures of this model to test senolytic drugs^15,19^. Embryonic pituitaries at 18.5 days post-coitum (18.5 d.p.c.) were dissected and cultured ex vivo with or without NMTi IMP1088 (Fig. 7g). IMP1088 eliminated senescent cells, as assessed by a significant decrease in β-catenin-positive and β-catenin-positive/p21^Cip1^-positive cells (Fig. 7h, Extended Data Fig. 7f,g and Supplementary Fig. 17a) by selectively inducing apoptosis (Fig. 7h,i). IMP1088 did not affect other cell types in the pituitary, such as hormone-producing cells that express synaptophysin (Extended Data Fig. 7h)^54^.

Next, we employed a model of liver tumour initiation in which senescence is induced in hepatocytes by transposon-mediated transfer of oncogenic NRAS (NRAS^G12V^)^55^. We expressed NRAS^G12V^ in livers, taking advantage of hydrodynamic tail-vein injection (HDTVI), and treated a cohort with the NMTi IMP1320 (Fig. 7j). Mice treated with the NMTi displayed reduced numbers of NRAS-positive senescent hepatocytes, as assessed by reduced staining of NRAS (Fig. 7k), SA-β-Gal (Fig. 7l) and p21^CIP1^ (Fig. 7m). Together, these results imply that NMTi phenocopy COPI inhibition and can be used as senolytic drugs in vivo.

### NMTi improve fibrosis and NASH-induced liver steatosis

**Extended Data Fig. 8:**
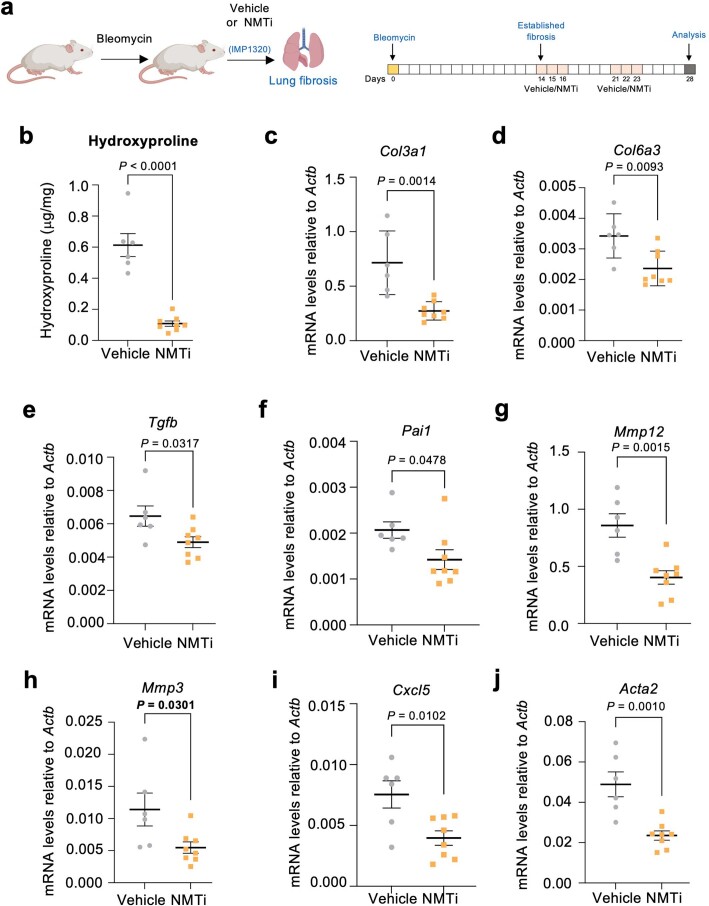
The therapeutic benefit of NMTi in a model of bleomycin-induced lung fibrosis. **a**, Experimental design of the mouse model of bleomycin-induced lung fibrosis. All analyses were performed at 28 days after treatment with bleomycin (intratracheal 0.75 U/kg) into the lungs of 6-8 weeks old, C57BL/6 J male mice. **b**, Lung hydroxyproline content in samples from mice of the experiment described here. (n = 6 mice, vehicle; n = 8 mice, NMTi). **c-j**, Relative expression of the indicated mRNAs in lung samples (n = 6 mice, vehicle; n = 8 mice, NMTi). Data represented as mean ± SD throughout figure. Unpaired two-tailed t-test used throughout figure. N represents mice throughout figure. Source numerical data are available in source data. Source data

**Extended Data Fig. 9:**
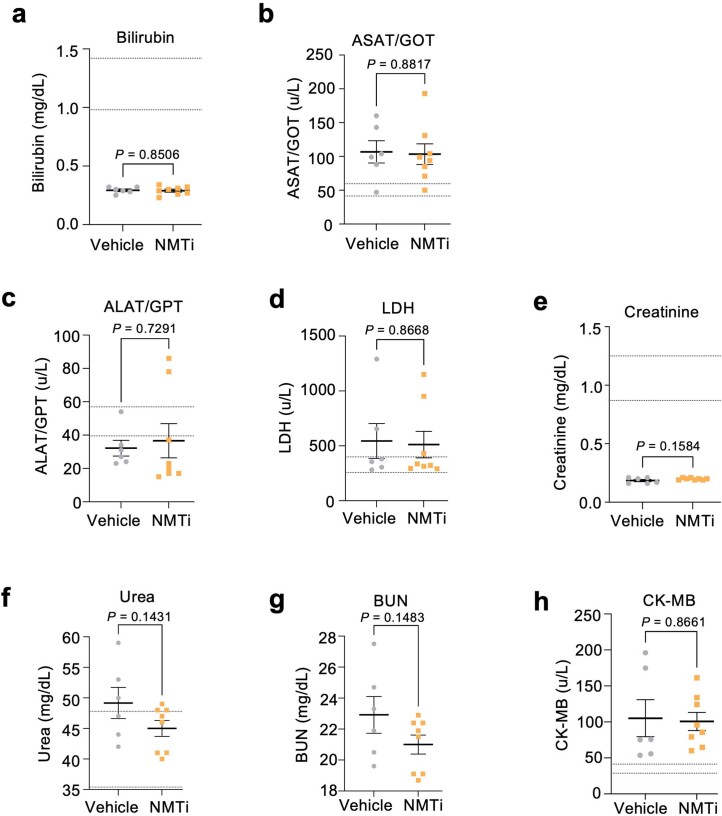
Treatment with NMTi does not induce toxicities in a model of bleomycin-induced lung fibrosis. **a-h**, Bilirubin (a), ASAT/GOT (b), ALAT/GPT (c), LDH (d), Creatine (e), Urea (f), BUN (g) and CK-MB (h) were measured in the blood of mice subjected to the bleomycin-induced lung fibrosis experiment (described in Extended Data Fig. 8), showing no significant increase in toxicity associated with the NMTi treatment. Dotted lines indicate normal range of the different metabolites. (n = 6 mice, vehicle; n = 8 mice, NMTi). Unpaired, two-tailed t-test. Data represented as mean ± SD. Source numerical data are available in source data. Source data

Elimination of senescent cells has a positive impact on many age-related phenotypes and diseases^4^, including idiopathic pulmonary fibrosis (IPF)^56^ or non-alcoholic steatohepatitis (NASH)^57^. To understand the potential of NMTi as a senotherapy for IPF, we used a model of bleomycin-induced lung fibrosis. We subjected six- to eight-week old C57BL/6J male mice to a single intratracheal dose of bleomycin (0.75 U kg^−1^) and treated them with the NMTi IMP1320 (Extended Data Fig. 8a). Lungs from animals treated with NMT inhibitor showed reduced levels of hydroxyproline compared to the control group, suggestive of reduced fibrosis (Extended Data Fig. 8b). Similarly, expression levels of collagen genes, pro-fibrotic factors, metalloproteinases, the inflammatory cytokine Cxcl5 and alpha-smooth muscle actin (α-SMA) were all significantly decreased in the NMTi-treated group compared to controls (Extended Data Fig. 8c–j). Profiling different blood biomarkers did not unveil any toxicities induced by NMTi treatment, but we observed an elevation in some markers, which could be attributed to bleomycin treatment (Extended Data Fig. 9). These findings show that treatment with an NMT inhibitor is well tolerated and leads to reduced fibrosis in a model of IPF.

**Fig. 8:**
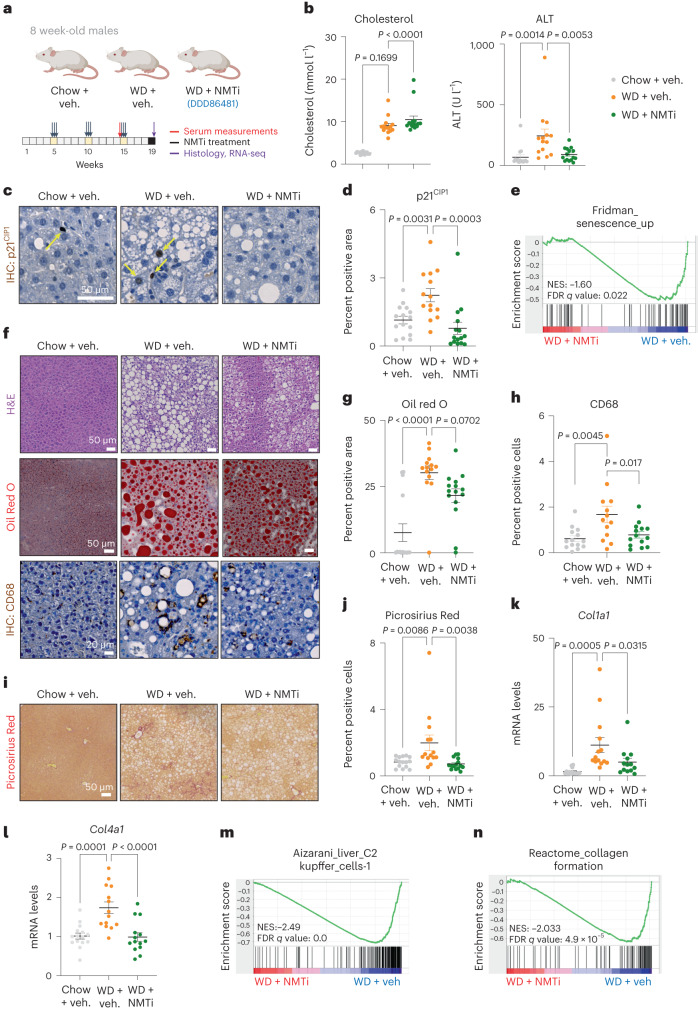
NMTi eliminate senescent cells and improve NASH-induced liver steatosis and fibrosis. **a**, Experimental design for the model of WD-induced NASH. **b**, Quantification of blood serum levels of cholesterol and ALT in normal, WD mice treated with vehicle (Chow+veh, *n* = 15, WD+veh, *n* = 14) or WD mice treated with DDD86481 (WD+NMTi, *n* = 15). Ordinary one-way ANOVA. **c**,**d**, Representative images (**c**) and quantification (**d**) of p21^CIP1^ staining of liver sections. Yellow arrows in **c** indicate examples of p21^CIP1^-positive cells. Scale bar, 50 μm. Chow+veh, *n* = 15; WD+veh, *n* = 14; WD+NMTi, *n* = 15. **e**, GSEA plot showing that a senescence signature is downregulated in WD-fed mice treated with NMTi. **f**–**h**, Representative images (**f**) of H&E (top), Oil Red O (middle) (chow+veh, *n* = 15; WD+veh, *n* = 14; WD+NMTi, *n* = 15) and CD68 IHC (bottom) stained liver sections (chow+veh, *n* = 14; WD+veh, *n* = 13; WD+NMTi, *n* = 14) (scale bars, 50 µm (H&E), 20 µm (Oil red O and CD68)) and quantification of Oil Red O staining (**g**) and CD68 staining (**h**). **i**,**j**, Representative images of Picrosirius Red-stained liver sections (**i**) and quantification (**j**). Scale bar, 50 μm. Chow+veh, *n* = 15; WD+veh, *n* = 14; WD+NMTi, *n* = 15. **k**,**l**, Levels of *Col1a1* (**k**) and *Col4a1* (**l**) mRNA from bulk liver extracts (chow+veh, *n* = 15; WD+veh, *n* = 14; WD+NMTi, *n* = 15). **m**,**n**, GSEA plots showing that senescence signature of Kupffer cells (**m**) and collagen formation (**n**) are downregulated in WD-fed mice treated with NMTi. Data are presented as mean ± s.e.m. Ordinary one-way ANOVA. *n* represents number of mice throughout the figure. Source numerical data are available as source data. Source data

**Extended Data Fig. 10:**
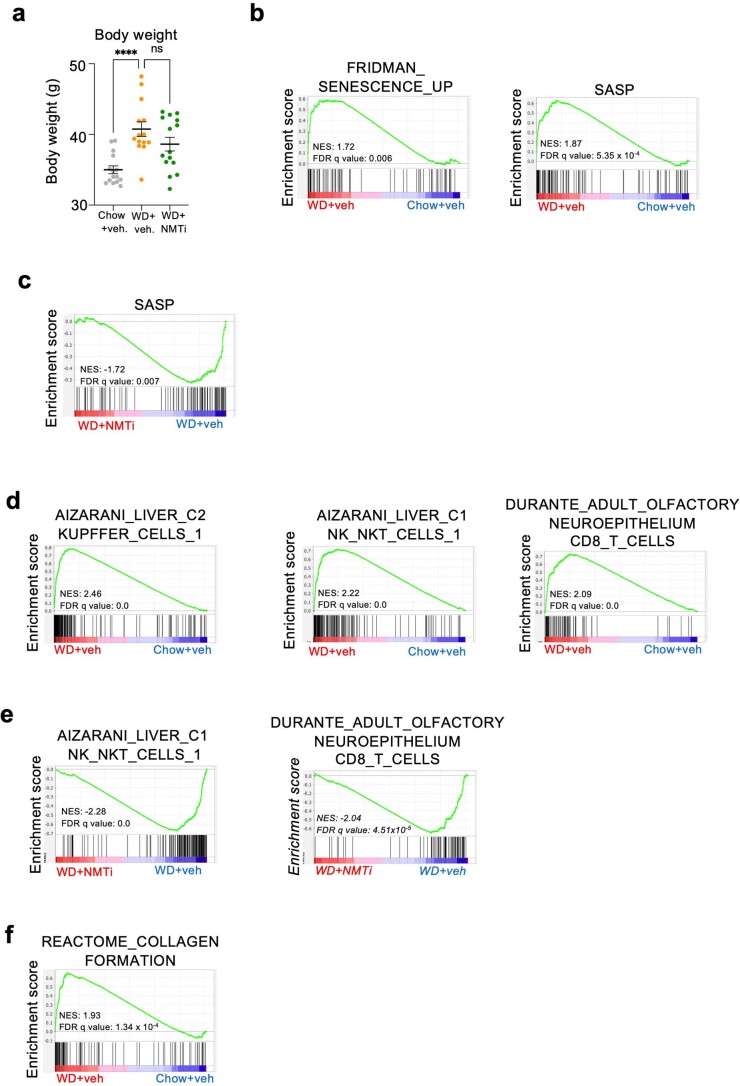
NMT inhibitors eliminate senescent cells and improve NASH-induced liver steatosis and fibrosis. **a**, Body weight of chow-fed (n = 15 mice) and western diet (WD)-fed mice treated with vehicle (n = 14 mice) or NMT inhibitor (n = 15 mice) following the last round of treatment. Ordinary One-way ANOVA. Dunnett’s test. **b-f**, GSEA plots showing the enrichment of the indicated signatures in mice fed WD (WD + veh) as compared with mice fed with chow diet (Chow + veh; b, d, f) or in mice fed with WD and treated with NMTi (WD + NMTi) as compared with mice fed with WD and treated with vehicle (WD + vehicle; c, e). NES, normalized enrichment score; FDR, false discovery rate. Source numerical data are available in source data. Source data

Next, we tested NMTi as a senolytic in a model of NASH. To this end, we fed eight-week-old males with a normal diet (chow) or a well-characterized western diet (WD)-based model of mouse NASH that is rich in fats and sugars (fructose and sucrose) for 19 weeks^58^. A cohort was treated with the NMTi DDD86481 for three consecutive days during weeks 5, 10 and 15, as summarized in Fig. 8a. Assessment at the end of the experiment showed a significant increase in body weight in the WD-fed mice and a non-significant trend of lower weight in the NMTi-treated cohort when compared with the WD-fed, vehicle-treated group (Extended Data Fig. 10a). Although both WD-treated groups showed significantly higher levels of serum cholesterol, the cohort treated with NMTi had lower levels of serum alanine aminotransferase (ALT; Fig. 8b), suggestive of reduced liver damage.

GSEA analysis showed an enrichment of senescence and SASP signatures in mice fed with WD when compared with the cohort fed with a chow diet (Extended Data Fig 10b). Quantitative immunohistochemistry (IHC) of whole liver sections showed a significant increase in p21^CIP1^-positive cells in the cohort fed with WD and a significant reduction in NMTi-treated mice (Fig. 8c,d), suggesting that NMTi treatment caused a reduction of p21^CIP1^-positive senescent cells. Signatures of senescence and SASP were indeed downregulated in the NMTi-treated cohort when compared with WD-fed vehicle-treated mice (Fig. 8e and Extended Data Fig. 10c).

H&E-stained liver sections showed increased hepatic steatosis in WD-fed mice that was less pronounced in the NMTi-treated cohort (Fig. 8f, upper panels). To directly assess how NMTi affected lipid accumulation and liver steatosis, we stained lipids in liver sections using Oil Red O. Oil Red O staining revealed an increased accumulation of lipid deposits in the livers of WD-fed mice when compared with chow-fed mice that reduced upon NMTi treatment (Fig. 8f, middle panels and Fig. 8g).

NASH is associated with chronic inflammation that results in the recruitment and activation of different immune cell populations^59,60^. WD-fed mice displayed a significant increase in macrophages/monocytes (as assessed by CD68 staining), which was not observed in the NMTi-treated cohort (Fig. 8f, lower panels and Fig. 8h), suggesting that NMTi treatment might reduce liver inflammation.

Fibrosis is a primary determinant of outcome in NASH^61^. Using Picrosirius Red staining, we observed a significantly lower fibrotic area in liver sections from mice fed with WD and treated with NMTi when compared with their vehicle-treated counterparts (Fig. 8i,j). Consistent with these results, the expression of collagens (*Col1a1* and *Col4a1*) was higher in mice fed with WD than in their chow diet-fed counterparts and was found to be significantly decreased in the NMTi-treated group when compared with the WD + vehicle cohort (Fig. 8k,l).

Finally, we used GSEA to corroborate these observations. GSEA suggested an increased presence of different immune cells, including Kupffer cells, NKT cells and CD8 T cells, in the livers of mice fed with WD (Extended Data Fig. 10d). These immune cells have been linked with NASH progression^59,60,62^. Conversely, these immune gene signatures were found to be downregulated in the NMTi-treated cohort (Fig. 8m and Extended Data Fig. 10e). Moreover, gene signatures related to collagen were upregulated in the WD + vehicle cohort (Extended Data Fig. 10f), but downregulated in their NMTi-treated counterparts (Fig. 8n). Overall, the above results show that treatment with NMTi reduced senescence, inflammation, steatosis and fibrosis in a WD-induced mouse model of NASH.

## Discussion

Here we have described the identification of components of the COPI pathway, which regulates a variety of dynamic membrane-trafficking events^24^ in RNAi screens, to identify the vulnerabilities of senescent cells. Cells undergoing senescence reorganize their endomembrane system to cope with the increase in secretion necessary for the SASP^30^. Depletion of *COPB2* caused a more profound Golgi disruption in senescent cells compared to non-senescent cells, suggesting that their reorganized Golgi might increase their dependence on COPI. Interestingly, differential Golgi disruption was not observed upon treatment with GBF1 inhibitors. This could be due to a more profound and sustained effect of GBF1 inhibitors when compared with transient *COPB2* knockdown. Importantly, interfering with the secretory apparatus by disrupting COPI results in the aberrant accumulation of SASP components and in general misfolded proteins in senescent cells, which saturates autophagy and activates the UPR, explaining the enhanced sensitivity of senescent cells to COPI inhibition.

We investigated whether COPI inhibition could be beneficial for the outcome of cancer and fibrosis. Inhibiting COPI with BFA or GCA killed cancer cells that had been treated with chemotherapeutic agents. Moreover, *COPB2* depletion prevented the senescence-fuelled increase in tumour growth in a xenograft cancer model. and improved outcomes in a model of lung fibrosis^16,40^. Because interfering with COPI (or NMT) also impairs the SASP, we speculate that the benefits associated with COPI inhibition might be the combined result of SASP suppression and selective killing of senescent cells.

Despite the promising therapeutic effects associated with COPI inhibition, the poor pharmacological properties of existing drugs targeting the pathway (such as BFA) have hampered their use in the clinic^41^. Based on existing knowledge of COPI regulation^43,44,63,64^, we hypothesized that NMTi would phenocopy COPI inhibition. Indeed, treatment with NMTi reduced the levels of ARF GTPases, resulting in Golgi dispersal in senescent cells, intracellular accumulation of secreted cytokines, and UPR activation. More importantly, NMTi are potent senolytics. Although our results suggest that the senolytic effect of NMTi might be explained by their effect on COPI signalling, NMTi have a wider selective window than GBF1 inhibitors. This could reflect the different mechanisms of action of the two drugs, with GBF1 inhibitors directly targeting GBF1 and affecting ARF function, and NMTi inhibiting the myristoylation of newly synthesized proteins. Alternatively, additional targets besides the COPI pathway could contribute to explaining the senolytic effects of NMTi.

NMTi has been tested as an anticancer^51^ and antiviral^65,66^ treatment. Our results make the case for using the NMTi as senolytics. To evaluate their senolytic potential, we tested NMTi in models of cancer, NASH and IPF. Two different NMTi (IMP1088 or IMP1320) reduced the numbers of preneoplastic senescent cells in models of paediatric pituitary tumours and liver cancer while eliminating the tumour growth conferred by co-injected senescent cells in a xenograft cancer model. Treatment with NMTi reduced fibrosis in a model of IPF and resulted in decreased inflammation, steatosis and liver fibrosis in a NASH model. Therefore, our results encourage further development of NMTi to treat cancer and other senescence-associated pathologies.

In summary, our data identified COPI signalling and *N*-myristoylation as targetable vulnerabilities of senescent cells. Although existing GBF1 inhibitors (such as BFA and GCA, which target COPI signalling) are not appropriate for preclinical or clinical use, the most recent generation of NMTi show senolytic potential and hold promise for clinical development as senolytic medicines that could be used to target a wide range of senescence-associated pathologies.

## Methods

Our research complies with all relevant ethical regulations and guidelines. The lung fibrosis experiments were performed in compliance with guidelines established by the Barcelona Science Park’s Committee on Ethics for Animal Experimentation (CEEA) and under approved protocol no. 10884. All other mouse procedures were performed under licence, according to the UK Home Office Animals (Scientific Procedures) Act 1986, ARRIVE and local institutional guidelines. The mouse pituitary experiments were approved by the UCL ethical review committee (PPL P5FB9D417). Liver cancer initiation and the WD experiments were approved by the animal welfare and ethical review board at Imperial College London (PPL 70/09080 and PPL PE02064666, respectively). Cancer xenograft experiments were performed by national and international guidelines and were approved by the institutional review board at Southampton University (PPL P81E129B7).

### Drugs

The following compounds were used in the present study: ABT-263 (Selleckchem, S1001), etoposide (Sigma-Aldrich, E1383), QVD-OPh hydrate (Sigma-Aldrich, SML0063), 4OHT (Sigma-Aldrich, H7904), doxycycline hyclate (Sigma-Aldrich, D9891), doxorubicin hydrochloride (Cayman Chemical, 15007), triamcinolone (Selleckchem, S1933), beclomethasone dipropionate (Selleckchem, S3078), GSK2606414 (Tocris, 5107), GSK2656157 (Selleckchem, S7033), GCA (Selleckchem, S7266), BFA (Selleckchem, S7046), IMP1088 (Myricx), DDD86481 (Myricx), IMP1320 (Myricx) and bleomycin sulfate (Generon, A10152).

### Antibodies

The following primary antibodies were used in this study: mouse monoclonal anti-BrdU (3D4, BD Biosciences, 555627) 1:2,000, mouse monoclonal anti-p16^INK4a^ (JC8, CRUK) 1:1,000, rabbit polyclonal anti-glyceraldehyde 3-phosphate dehydrogenase (anti-GAPDH) (Abcam, ab22555) 1:2,000, mouse monoclonal anti-IL-8 (6217, R&D systems, MAB208) 1:100, goat polyclonal anti-IL-6 (R&D Systems, AF-206-NA) 1:40–1:200, mouse monoclonal anti-ARF1/3/5/6 (1D9, Invitrogen, MA3-060) 1:500, rabbit monoclonal anti-COPB2 (899, gifted from F. Weiland) 1:10,000, mouse monoclonal anti-EEA1 (14, BD Biosciences, 610457) 1:200, rabbit polyclonal anti-XBP1 (Abcam, ab37152) 1:200, rabbit polyclonal anti-ATF6 (Abcam, ab37149) 1:500, sheep polyclonal anti-TGN46 (Bio-Rad, AHP500G) 1:400, mouse monoclonal anti-GM130 (35, BD Biosciences, 610822) 1:500, mouse monoclonal anti-CHOP (L63F7, CST, 2895S) 1:1,000, rabbit monoclonal anti-p21^CIP1^ (12D1, CST, 2947S) 1:2,000, rabbit monoclonal anti-p21^CIP1^ (EPR18021, Abcam, ab188224) 1:700, mouse monoclonal anti-N-Ras (F155, Santa Cruz, sc-31) 1:100, mouse monoclonal anti-β-catenin (6F9, Sigma, C7082) 1:500, rabbit polyclonal anti-β-catenin (Thermo, RB-9035-P1) 1:500, mouse monoclonal anti-synaptophysin (27G12, Leica, SYNAP-299-L) 1:200, rabbit polyclonal anti-CC3 (CST, 9661S) 1:1,000, goat polyclonal anti-CXCL1 (R&D, AF-275) 1:100, mouse monoclonal anti-BMP2/4 (100230, R&D, MAB3552), mouse monoclonal anti-VEGF (23410, R&D MAB2931) 1:100, mouse monoclonal anti-GM-CSF (3209, R&D, MAB215) 1:100, rabbit polyclonal anti-CD68 (Abcam, ab125212) 1:100, rabbit polyclonal anti-ARF1 (10790-1-AP, Proteintech) 1:1,000, rabbit polyclonal anti-ARL1 (16012-1-AP, Proteintech) 1:1,000, rabbit polyclonal anti-PPM1B (HPA-016745, Cambridge Bioscience) 1:1,000 and rabbit polyclonal, mouse monoclonal anti-TUBA (ab1729, Abcam) 1:1,000.

We used the following secondary antibodies: goat anti-mouse IgG-HRP (immunoglobulin-G–horseradish peroxidase; Santa Cruz, sc-2005) 1:2,000, goat anti-rabbit IgG-HRP (Santa Cruz, sc-2004) 1:2,000, goat anti-mouse IgG (H + L) AlexaFluor488 conjugated (Invitrogen, A-11029) 1:2,000, goat anti-mouse IgG (H + L) AlexaFluor594 conjugated (Invitrogen, A-11032) 1:2,000, goat anti-rabbit IgG (H + L) AlexaFluor594 conjugated (Invitrogen, A-11037) 1:2,000, donkey anti-sheep IgG (H + L) AlexaFluor594 conjugated (Invitrogen, A-11016) 1:2,000 and donkey anti-sheep IgG (H + L) AlexaFluor488 conjugated (Invitrogen, A-11015) 1:2,000. For the IpaJ western blot experiments, we used the following secondary antibodies: IRDye 800CW goat anti-rabbit IgG (H + L, 926-32211, Li-Cor, 1:10,000)and IRDye 800CV goat anti-mouse IgG (H + L, 926-32210, Li-Cor, 1:10,000).

### Cell lines

IMR90 (ATCC, CCL-186), SK-HEP-1 (ATCC, HTB-52), A549 (ATCC, CCL-185), HFFF2 (ECACC, 86031405), HCT116 (ATCC, CCL-247), MCF7 (ATCC, HTB-22), 5PT^39^ (a gift from I. C. Mackenzie, QMUL), PBEC (ATCC, PCS-300-010) and NHLF (Lonza, CC-2512). PBECs were cultured in airway epithelial cell basal medium (ATCC-PCS-300-030; ATCC) supplemented with bronchial epithelial cell growth kit supplements (ATCC-PCS-300-040; ATCC) and 0.1% antibiotic–antimycotic solution (Gibco) with media replenished every 48 h. Adult NHLFs were cultured in fibroblast basal medium (CC-3131; Lonza) supplemented with a SingleQuot Kit of supplements and growth factors (CC-4126; Lonza), with media replenished every three to four days as required. All other cells lines were maintained on Dulbecco’s modified eagle medium (DMEM; Gibco) supplemented 1% 100X Gibco antimycotic-antibiotic and 10% (vol/vol) FBS (Labtech, Batch 41213), hereinafter referred to as DM10 medium. Passaging of cells was performed by enzymatic detachment using 0.05% Trypsin-EDTA (Gibco) on cells for 5 min, followed by inactivation in DM10 medium and centrifugation at 180*g* for 5 min. The supernatant was aspirated to remove dead cells and debris, and the pellet was resuspended in fresh DM10. Cell numbers were determined using a Guava EasyCyte platform (Millipore) using Guava ViaCount reagent. In-built GuavaSoft software was used to define live cells and remove cell debris/dead cells from the final cell count. Experiments using IMR90 cells or cell lines generated from them were carried out using cells between passages 10 and 14. To generate ER:RAS^GV12^ and other derived cells, IMR90 or HFFF2 cells, retroviral and lentiviral infections were carried out as described in ref. ^32^. Treatment with 100 nM 4-OHT (Sigma, in DMSO) was used to induce IMR90 ER:RAS cells to undergo OIS. Therapy-induced senescence (TIS) was induced in IMR90 cells by treatment with 33 μM (50 µg ml^−1^) bleomycin sulfate (Generon, A10152) for 24 h, 20 μM palbociclib (Selleckchem, S1116) for seven days or 100 nM doxorubicin (Cayman Chemical, #15007) for seven days. Senescence was induced in A549 and SK-HEP-1 cells by treatment with 2 μM etoposide (Sigma-Aldrich, E1383) for seven days. HCT116 senescence was induced by 100 nM treatment with doxorubicin (Cayman Chemical, 15007) or 2 µM etoposide for three days, followed by four days culture in medium without chemotherapy. Senescence was induced in MCF7 by treatment with 200 nM doxorubicin or 2 µM etoposide for seven days.

### Mice

All mice were purchased from Charles River UK Ltd except where noted otherwise.

For HDTVI experiments, female C57BL/6J mice aged five to six weeks were given 20 μg of a vector expressing Nras^G12V^ and Gaussia luciferase (Gluc) along with 5 μg of SB13 transposase-expressing plasmid. Experiments were performed as described in ref. ^55^. Four days after HDTVI, mice were bled to assess the presence of a Gaussia luciferase signal in the blood plasma and used to randomize groupings for vehicle and drug-treated groups. On day 5, mice were given 25 mg kg^−1^ of IMP1320 (*n* = 9 mice) or vehicle (*n* = 9 mice) (10 mM Na_2_HPO_4_-7H_2_O and NaH_2_PO_4_H_2_O buffer, 0.2% Tween-80, pH 7.4) intraperitoneally (i.p.) daily for four days. Twenty-four hours after the last drug injection, mice were culled and livers collected for paraffin embedding and frozen in optimal cutting temperature compound (OCT).

For cancer xenograft experiments, 6.7 × 10^5^ 5PT cells ± 2 × 10^6^ HFFF2 cells were injected subcutaneously (s.c.) into the flanks of immunocompromised, male NOD SCID Gamma (NSG) mice (three to five months old). For knockdown experiments, HFFF2 fibroblasts expressing inducible shRNAs targeting *COPA*, *COPB2* or control were irradiated at 10 Gy using a MultiRad350 X-ray irradiation cabinet (from Precision X-ray) just before implantation. In vivo, expression of the shRNA was induced using doxycycline given in drinking water throughout the experiment (2 mg ml^−1^ with 5% sucrose in the drinking water). Experiment A (Fig. 5e) included six mice for shCOPB2.1 + irradiation and seven mice for all other groups. In experiment B (Supplementary Fig. 12f) *n* = 7 mice per group were utilized. The data presented in Extended Data Fig. 5d compile all mice used in experiment A and experiment B. For the control experiment described in Extended Data Fig. 5e, *n* = 6 mice were included in the 5PT group and *n* = 8 mice in all other groups.

For xenograft experiments with NMTi (Fig. 7e and Extended Data Fig. 7f), 10 mg kg^−1^ NMTi (DDD86481) was dissolved in water containing 5% DMSO, 20% PEG400, 10 mM Na_2_HPO_4_.7H_2_O and NaHPO_4_H_2_0 buffer, 0.5% Tween-80, pH 7.3, and administered by intraperitoneal (i.p.) injection as indicated in Fig. 7e. Tumour size was measured over time using an electronic caliper and calculated using the formula 4π/3 × *r*^3^ (radius (*r*) calculated from the average diameter, measured as the tumour width and length). For this experiment, *n* = 6 mice were utilized for the 5PT + vehicle treatment group and *n* = 8 mice per group for all other groups. We did not exceed the maximal tumour size permitted under the licence (1,750 mm^3^) during the experiments. The area under the curve (AUC) for each tumour within a treatment group for single experiments was analysed, and statistical analysis comparing the AUCs was performed on pooling multiple experiments.

For testing senolytics ex vivo in the ACP model of OIS, neoplastic pituitaries from 18.5dpc *Hesx1*^Cre/+^;*Ctnnb1*^lox(ex3)/+^ embryos^67^ were dissected. Both male and female embryos were used, and the numbers were equalized in experimental and control groups. For the experiments shown in Fig. 7h,i and Extended Data Fig. 7h, *n* = 8 18dpc embryos were dissected (*n* = 5 embryonic pituitaries were treated with vehicle and *n* = 3 with IMP1088). For the experiments described in Extended Data Fig. 7f,g, *n* = 14 18dpc embryos were dissected (*n* = 5 embryonic pituitaries were fixed at *t* = 0 h; *n* = 3 embryonic pituitaries were fixed at *t* = 24 h; *n* = 3 embryonic pituitaries were fixed at *t* = 48 h; *n* = 3 embryonic pituitaries were fixed at *t* = 72 h). In both sets of experiments, after treatment and fixation, pituitaries were sectioned and stained (the specific numbers of sections stained and analysed for each experiment are described in the figure legends). For the lung fibrosis experiments, we used a previously described mouse model of lung fibrosis induced by intratracheal administration of senescent human cells^16,40^. Normal proliferating (IMR90 vector) or gamma-irradiated senescent human fibroblasts IMR90 (IMR90 vector, IMR90 shCOPB2.1 or IMR90 shCOPB2.2) (500,000 cells) were delivered into the lungs of six- to eight-week-old athymic (nu/nu) male mice (Envigo Laboratory). Two days before intratracheal instillation, these animals started treatment with doxycycline (1 mg ml^−1^ in the drinking water) until the end of the experiment. Three weeks after intratracheal instillation, their lungs were removed and analysed. To estimate the number of senescent IMR90 cells engrafted in the lung after 48 h post-instillation, we first performed a calibration using a known amount of IMR90 cells mixed with lung homogenates. Specifically, the right lobes of nude mice were surgically dissected and placed into 1.5-ml tubes. Homogenates of the lung samples were performed by grinding the frozen samples with liquid nitrogen using a mortar and pestle. Tissues were then thawed, 1 ml of distilled water was added to the tissues, and the resulting suspensions were homogenized using a micro-sample homogenizer (Precellys). Different quantities of senescent shControl IMR90 cells (0, 1,000, 5,000, 10,000, 50,000 or 100,000 cells) were mixed with 1 ml of homogenized lung tissue. After Trizol extraction of RNA and cDNA synthesis using SuperScript III reverse transcriptase (Thermo Fisher), real-time qPCR was performed using the PowerUp SYBR Green Master Mix (Applied Biosystems). Gene expression analysis was performed using predesigned primers and probes for human *MMP3*. Data were normalized using mouse *Actin b*. The resulting calibration curve was used to interpolate the data obtained using lung samples 48 h post-intratracheal instillation of shControl, shCOPB.1 and shCOPB.2 IMR90 cells (500,000 cells) in nude mice under doxycycline. For lung fibrosis experiment, *n* = 5 mice per group were used, and for validation of engraftment, *n* = 3 mice per group were utilized.

For the mouse model of bleomycin-induced lung fibrosis, pulmonary fibrosis was initiated by intratracheal instillation of bleomycin (0.75 U kg^−1^) into the lungs of six- to eight-week-old male C57BL/6J mice (Envigo Laboratory). Fourteen days after intratracheal instillation, once the mice had developed well-established pulmonary fibrosis, these animals started treatment with NMTi (IMP1320; 10 mg kg^−1^, i.p.) or vehicle, administered in a cyclical pattern, consisting of three consecutive days of treatment followed by three consecutive days without treatment. Two weeks after treatment started, the lungs were removed and analysed. For these experiments, we used *n* = 8 mice per group. Two mice in the vehicle-treated group died before the end of the experiment and were not included in any analysis.

For WD experiments, C57BL/6J male mice aged eight weeks (*n* = 15 for each of the three groups) were placed on chow (4.25% fat, RM3, Special Diet Services) or WD (Research Diets, D16022301; 40% kcal fat (non-trans-fat Primex shortening), 22% (wt:wt) fructose, 10% (wt:wt) sucrose and 2% (wt:wt) cholesterol) for four weeks before the first round of injections. Mice were then injected i.p. daily with vehicle (10 mM Na_2_HPO_4_.7H_2_O and NaH_2_PO_4_H_2_O buffer, 0.2% Tween-80, pH 7.4) or 10 mg kg^−1^ DDD86481 (5% DMSO, 20% PEG400, 10 mM Na_2_HPO_4_.7H_2_O) and NaH_2_PO_4_-H_2_O buffer, 0.5% Tween-80, pH 7.3, dissolved by cold water bath sonication, for three days, then given two rounds of a four-week rest period and three-day daily i.p. injection. Blood was collected before the final round of injection for physiological assessments. Mice were allowed to rest for four weeks before being culled and organs were collected for freezing in OCT, paraffin embedding, blood collection for physiological measurements, and tissue snap-freezing for RNA extraction. A mouse of the WD + vehicle group died before the end of the experiment and was not included in any analysis.

To assess the effect of NMTi on metabolic function, a cohort of C57BL/6J male mice aged eight weeks were treated with either vehicle (*n* = 5 mice), 10 mg kg^−1^ DDD86481 (*n* = 5 mice) or 25 mg kg^−1^ IMP1320 (*n* = 4 mice), then blood was collected after seven days Supplementary Fig. 16a,b). To assess the effect of NMTi on insulin secretion (Supplementary Fig. 16c), 5 µl of blood was collected after seven days from mice treated with either vehicle (*n* = 5 mice) or 10 mg kg^−1^ DDD86481 (*n* = 6 mice). Blood was processed using an Ultra Sensitive Mouse Insulin ELISA kit (Crystal Chem) according to the manufacturer’s instructions. To assess the effect of NMTi on immune cell composition, C57/BL6 mice were treated with either vehicle, 10 mg kg^−1^ DDD86481 or 25 mg kg^−1^ IMP1320, and blood was collected one day or seven days after treatment (day 1, *n* = 5 mice per group; day 7, *n* = 3 mice per group).

### Vector construction

pLNC-ER:RAS-neo has been described previously in ref. ^68^. The mutant GBF1_M832L_ construct was a gift from F. J. M. van Kuppeveld (Utrecht University). Cloning of GBF1_M832L_ intro retroviral expression vector (pBabe-puro) was performed by PCR amplification using Human5SnaBIGBF1 (5′-CGTACGTAGCCATGGTGGATAAGAATATTT-3′) and Human3SalIGBF1 (5′-CGGTCGACGCCTTAGTTGACCTCAGAGGTG-3′) primers with Q5 High-Fidelity DNA polymerase (New England Biosciences) according to the manufacturer’s instructions. Amplified GBF1_M832L_ was subcloned into pBabe-puro using standard cloning with SnaBI and SalI restriction enzymes. Gaussia luciferase (Gluc) containing plasmid was a gift from U. Griesenbach (Imperial College London). To generate Gluc expressing HDTVI construct (CaNiGluc), Gluc was PCR-amplified using 5BmgBIsogLUX: (5′-GATTAAGACG TGGTTTTCCT TTGAAAAACA CGATGATAAT ATGGGAGTGA AGGTGCTGTT-3′) and 3sogLUXAge1 (5′-TTTGTTACCG GTCTCATCAA TCTCCCCCAGCT-3′) primers and Q5 High-Fidelity DNA polymerase (New England Biosciences), according to the manufacturer’s instructions. Amplified Gluc was subcloned into HDTVI construct (CaNiG) by restriction enzyme excision of GFP and annealing of Gluc amplicon processed with BmgBI and AgeI into CaNiG plasmid. The IpaJ construct was a gift from E. Tate (Imperial College London). Cloning of IpaJ_WT/C64A_ was performed by PCR amplification of IpaJ using 5′EcoRIKozIpaJ (5′-tggtggaattcgccaccATGTCGGAACAACGGAAG-3′) and 3′IpaJPmeI (5′-agcaggtttaaacTTACAAAGCCTCATTAGT-3′) and subcloning into pLenti-puro vector (Addgene 39481) with EcoRI and PmeI restriction enzymes. Tetracycline inducible (Tet-ON all-in-one) shRNA vector (LT3GEPIR) was a gift from J. Zuber (IMP, Vienna). The generation of miRE-based inducible shRNA vectors was performed as previously described^32^. The shRNA sequences used in this study are described in Supplementary Table 1.

### IF and high-throughput microscopy

IF staining was carried out by first fixing wells of 96-well plates at the desired timepoint for 1 h using 4% paraformaldehyde (PFA; wt/vol, in phosphate-buffered saline (PBS)) followed by washing three times with PBS. Wells were then permeabilized using 0.2% Triton X-100 (vol/vol, PBS) for 10 min and then washed twice with PBS to halt permeabilization. Non-specific antibody binding was blocked by incubation with a blocking solution for 1 h at room temperature (r.t.). The blocking solution contained 1% bovine serum albumin (BSA; wt/vol, PBS) supplemented with 0.4% fish skin gelatin (vol/vol, PBS). Primary antibodies were diluted in blocking solution and wells were incubated with primary antibody solution for 1 h at r.t. For BrdU staining, primary antibody solution was supplemented with 0.5 U μl^−1^ DNase (Sigma) and 1 mM MgCl_2_, and the incubation times were reduced to 30 min. Following incubation, the primary antibody was then removed by washing three times with PBS. Secondary antibodies conjugated to Alexa-594 or Alexa-488 fluorophores were then diluted in blocking solution and added to wells to be incubated in the dark for 1 h. The secondary antibody was then removed by washing three times with PBS and nuclei counterstaining with 1 μg ml^−1^ 4′,6-diamidino-2-phenylindole (DAPI; wt/vol, PBS) for 10 min. Wells were then washed with PBS three times.

Immunofluorescence image acquisition was performed using an automated InCell Analyzer 2000 high-throughput microscope. Multiple 96-well plates were placed into stacks by a KiNEDx robotic arm (PAA) running Overlord software so that the plates could be sequentially loaded into, imaged and removed from the InCell microscope. Wells were imaged using a ×20 objective except for wells stained only with DAPI or Golgi-related staining, which were performed at ×10 and 40, respectively, then 2 × 2 binning of images was used to reduce the image file sizes. Fluorophores were imaged using pre-set ‘DAPI’, ‘Texas Red’ and ‘FITC’ wavelengths on the microscope for DAPI stain, AlexaFluor594 and AlexaFluor488, respectively. Eight, 24 and 18 fields per well were captured for the ×10, ×20 and ×40 objectives, respectively.

High-content image analysis was carried out using the InCell Investigator 2.7.3 software (GE Healthcare). DAPI nuclear counterstain was used to segment cells using a top-hat method and used to provide a mask for nuclear-localized stains. For cytoplasmic stains, a 6-μm collar was applied around the cell and, for detection of cytoplasmic organelles such as Golgi, a ‘region growing’ collar was used. Quantification for nuclear staining was measured as the average pixel intensity (greyscale) for the wavelength of fluorophore across the area of the nuclear mask. Cytoplasmic staining quantification was of either the average pixel intensity or the coefficient of variance of pixel intensities within the collar area. Golgi structural analysis utilized a multiscale top-hat segmentation method to detect organelle structures between 1 and 3 pixels in size within a region growing collar. Cells with >25 Golgi organelle structures per cell were classified as cells with dispersed Golgi.

### Growth assays

BrdU incorporation and colony formation assays were performed as previously described in ref. ^38^. Briefly, for BrdU incorporation assays, cells were incubated with 10 μM BrdU for 18 h before being fixed using 4% PFA (vol/vol, PBS). BrdU incorporation was assessed via IF and high-content analysis. For crystal violet staining, cells were seeded at low density in 10-cm dishes and cultured for 10–14 days or until proliferating cells had reached 80–90% confluency. To assess senolysis, cells were seeded in 10-cm plates at high density. Senolytic drugs were added at their indicated concentration in DMSO (<0.5% vol/vol final concentration) and cultured for a further three days. If longer drug treatment was required, fresh drug and media were added on day 3 and cultured for a further four days. At the endpoint, plates were fixed with 0.5% (wt/vol, PBS) glutaraldehyde (Sigma) for 1 h, washed twice with dH_2_O, and left to dry overnight. Dried plates were then stained with a 0.2% (wt/vol, PBS) solution of crystal violet (Sigma, C6158).

### Senolytic assays

Senolytic assays were performed as described previously^15^. Briefly, at the indicated timepoints, confluent senescent or control cells in 96-well plates were switched to DMEM 0.5% FBS and drugs in DMSO were added (<5% vol/vol final concentration). Drugs were replenished after three days if the assay length was longer than 72 h. For TIS of PBECs (ATCC-PCS-300-010), cells were seeded at passage 3 and treated with bleomycin (100 ng ml^−1^) or vehicle for five days, followed by washout. Seven days post senescence induction, cells were treated with the indicated drugs for 72 h. Adult NHLFs (Lonza CC-2512) at passages 4 to 5 were seeded into 96-well plates and induced to senesce by treatment with bleomycin (50 mg ml^−1^), or vehicle, for 24 h. Seven days post-induction of senescence, cells were treated with the indicated drug concentrations for 72 h. Cells were fixed and stained with DAPI, followed by assessment by automated microscopy. The percentage survival was calculated by dividing the number of cells post-drug treatment by the corresponding number of cells treated with the vehicle at the same time.

For senolytics assays during replicative senescence, PBECs were serially passaged until passages 4–6, whereby a mixed population of senescent and growing cells can be distinguished. PBECs were plated into 96-well plates and treated with the indicated drug for 72 h. Cells were fixed and stained with anti-p16 antibody and DAPI, followed by assessment by automated microscopy. The percentage survival for p16-negative and p16-positive fractions was calculated by dividing the number of cells post-drug treatment by the number of cells treated with the vehicle.

### Tissue processing

Organs were fixed in 4% PFA overnight before being transferred to 70% ethanol. Tissue processing before paraffin embedding was performed on a Sakura Tissue-Tek VIP 6 automated tissue processor. Briefly, specimens in embedding cassettes were dehydrated by progressing through steps of 70% ethanol for 45 min at 37 °C, 80% ethanol for 45 min at 37 °C, 90% ethanol for 30 min at 37 °C, 96% ethanol for 45 min at 37 °C, 100% ethanol for 30 min at 37 °C, 100% ethanol for 1 h at 37 °C and 100% ethanol for 1 h at 37 °C. Dehydrated samples were then cleared by three washes in xylene for 30 min, 45 min and 1 h at 37 °C. Finally, the specimens were infiltrated by two immersions in 62 °C paraffin wax for 45 min and 1 h, followed by two immersions in 62 °C paraffin wax for 30 min. The specimen was then embedded in a paraffin block on an embedding centre (Leica EG1160), and 4-μm sections were made using a Thermo Fisher scientific microtome (Microm HM355S) and attached to slides.

### IHC staining

The slides were deparaffinized by washing them twice in Histoclear for 5 min each, followed by 5-min washes in decreasing concentrations of ethanol (100%, 75%, 50% and 25% ethanol) before a final wash of 5 min in dH_2_O. Heat-induced epitope retrieval (HIER) was then performed in a pressure cooker for 20 min using either antigen-unmasking solution, citrate-based at pH 6.0 (VectorLab, H-3300-250), or antigen-unmasking solution, Tris-based at pH 9.0 (VectorLab, H-3301-250), depending on the antibody manufacturer’s instructions. Following HIER, slides were cooled on ice for 10 min and then washed in PBS for 5 min. For intracellular stains, sections were permeabilized with 0.2% Triton X-100 in PBS for 10 min and washed twice in PBS for 5 min. For NRAS staining, liver slides were washed in 0.1% H_2_O_2_ in PBS for 15 min, followed by washing twice in PBS to reduce endogenous peroxide activity. Sections were marked using a hydrophobic pen, and non-specific antigen binding was blocked by incubating the slides with CAS-Block histochemical reagent (Thermo Fisher, 008120) for 30–45 min in a humidified chamber. The slides were then incubated with primary antibody overnight in a humidified chamber at 4 °C. Slides were washed twice in PBS for 5 min and incubated with secondary antibody SignalStain Boost IHC detection reagent with mouse HRP (Cell Signalling Technology, 8125) or rabbit HRP (Cell Signalling Technology, 8114) for 30–45 min. Next, the slides were washed twice in PBS for 5 min and incubated for 2–10 min with a SignalStain DAB substrate kit (CST, 8059) to detect the HRP signal. Signal development was stopped when visible positive cells could be detected on a microscope, by washing slides in dH_2_O. To counterstain the DAB signal, slides were incubated for 30 s in modified Mayer’s haematoxylin (Lillie’s modification; DAKO), washed in dH_2_O, and incubated for 30 s in 0.05% ammonium solution (PBS) followed by washing in dH_2_O. Before mounting the coverslips with VectaMount aqueous mounting medium (VectorLab, H-5501-60), the slides were dehydrated by washing for 1 min in 75% ethanol, 5 min in 100% ethanol and 5 min in Histoclear. Slide images were acquired using a ×20 bright-field objective on a Zeiss AxioScan Z.1 slide scanner, and analysis was performed on fields using QuPath version 0.2.0-m9 using an in-built positive cell detection tool to segment haematoxylin-stained nuclei and quantify the mean intensity of DAB.

### Histologic analysis of the mouse fibrosis experiment

Left lung tissue was fixed in a 10% neutral buffered formalin solution for 24 h and subsequently transferred into tissue cassettes and placed into PBS for a minimum of 24 h. The tissues were then shipped to the Institute for Research in Biomedicine (IRB) Histopathology Facility for paraffin embedding, sectioning and Masson’s trichrome and haematoxilin and eosin staining. Samples were examined first in a blinded fashion and in a second round in an unblinded fashion. Semiquantitative histological scoring of fibrosis was scored at ×20–40 using the following scale: 1, ×1; 2, ×2; 3, ×3 increase in the thickening of alveolar walls; 4, >×3 thickening of alveolar walls and focal areas of single fibrotic masses. If there was difficulty in deciding between two scores, the intervening number was given.

### Hydroxyproline assay

Superior and middle lung lobes were surgically dissected, weighed and placed into 1.5-ml sterile tubes and flash-frozen until all the samples were collected. Homogenates of the lung samples were made by grinding the frozen samples with liquid nitrogen using a mortar and pestle. On the day of the assay, tissues were thawed, and 1 ml of distilled water was added to the tissues. Tissues were homogenized using a micro-sample homogenizer (Precellys), then 200 µl of 12 N hydrochloride was added to 200 µl of homogenized tissues. The samples were placed into a preheated oven set to 120 °C and incubated overnight. The next morning, samples were cooled and vortexed. Biochemical quantification of hydroxyproline was performed using a hydroxyproline assay kit (Amsbio).

### Senescence-associated β-galactosidase assay

Cells grown in six-well plates were fixed with a solution of 0.5% glutaraldehyde (wt/vol, PBS; Sigma) for 10 min and washed twice in a solution of 1 mM MgCl_2_/PBS (pH 6.0). For staining, the plates were incubated with X-gal staining solution for 18 h at 37 °C. Images were acquired by bright-field microscopy using an inverted microscope (Olympus CKX41) with an attached digital camera (Olympus DP20). Cells were counted using ImageJ software to determine the percentage of positive cells.

Liver samples frozen in OCT were cryosectioned (15 μM), and the frozen sections were fixed in ice-cold 0.5% glutaraldehyde (wt/vol, PBS) for 15 min and washed 1 mM MgCl_2_/PBS (pH 6.0) for 5 min. The β-galactosidase activity was stained for with X-gal staining solution (1 mg ml^−1^ X-gal, Thermo Scientific, 5 mM K_3_(Fe(CN)_6_), 5 mM K_4_(Fe(CN)_6_)) diluted in 1 mM MgCl_2_/PBS (pH 6.0) for 18 h at 37 °C. Slides were dehydrated and coverslips mounted before being imaged using ×20 bright-field objective on a Zeiss AxioScan Z.1 slide scanner. ImageJ was used to quantify staining by measuring the SA-β-Gal-stained area as a percentage of the total tissue area excluding luminal spaces.

### Sirius Red staining

Sirius Red staining was carried out for collagen I/III fibre-containing connective tissue on paraffin-embedded sections using a Picrosirius Red stain kit (Abcam, ab150681). Before staining, sections were deparaffinized in Histoclear and graded ethanol washes as already described (IHC staining section), then hydrated in distilled water. Sections were then incubated with Picrosirius Red solution for 60 min at r.t. and then rinsed twice with 0.5% glacial acetic acid solution (in dH_2_O). Excess water was then removed by shaking the slides and then rinsing in 100% ethanol. Sections were then dehydrated by two washes of 100% ethanol for 2 min each and two washes in Histoclear for 2 min each. Coverslips were mounted and slides were imaged on a Zeiss AxioScan Z.1 system. Staining was quantified by thresholding the collagen-stained area for detection of fibres (red) and measuring this area relative to the total tissue area.

### Blood chemistry and immune cell composition analysis

For analysis of immune cell composition in whole blood, tail-vein blood was collected two days after the last treatment. Whole blood was diluted in saline to a volume of 200 μl and run on a Sysmex XE2100 automated cell counter. Blood glucose levels were determined by collecting whole blood from the tail vein into heparinized tubes (Abraxis), then 120–140 μl of whole blood was loaded onto a comprehensive diagnostic profile reagent rotor (Abraxis) or Mammalian Liver Profile reagent rotor and run on a VetScan VS2 Chemistry Analyzer (Abraxis, 500-7123).

### Oil Red O staining

Staining for lipids was carried out on liver tissue in OCT that was snap-frozen in liquid N_2_ and cryosectioned (15 µm). Sections were equilibrated to r.t. for 10 min and then stained with 0.5% Oil Red O solution (wt/vol, in isopropanol; Sigma, O1391) for 5 min, rinsed in tap water and counterstained with Mayer’s haematoxylin for 30 s. Sections were then rinsed again in tap water for 30 min and coverslips mounted. Images were acquired on a Zeiss AxioScan Z.1 system and ImageJ quantification of the Oil Red stain area was carried out relative to the background tissue area.

### Ex vivo culture of mouse pituitaries

Neoplastic pituitaries from 18.5dpc *Hesx1*^Cre/+^;*Ctnnb1*^lox(ex3)/+^ embryos were dissected and placed on top of 5 μM Nuclepore membranes (VWR) in 24-well plates containing 500 μl of medium (DMEM-F12 (Gibco), 1% pen/strep (Sigma) and 1% FBS (Thermo Fisher Scientific)) supplemented with either IMP1088 or vehicle (DMSO). The media were changed every 24 h, and the pituitaries were processed for analysis after 72 h. IF staining was performed as described in ref. ^53^. The proportion of β-catenin-accumulating cells was calculated as an index out of the total DAPI-stained nuclei. Over 120,000 DAPI nuclei were counted from 15 to 22 histological sections per sample, in a total of eight neoplastic pituitaries. The proportions of CC3 and synaptophysin-positive cells were calculated as an index out of the total tissue area, from 6 to 12 histological sections per sample.

### Immunoblotting

Cells were collected for protein extraction by first washing twice with ice-cold PBS, scraping, then centrifugation performed at 180*g* for 5 min at 4 °C. Cell pellets were then resuspended in RIPA lysis buffer (Thermo Scientific, 89900) supplemented with one tablet of PhosSTOP (Roche) and one tablet of cOmplete, Mini, EDTA-free Protease inhibitor (Roche). Lysis was performed on ice for 30 min with periodic vortexing. The lysis samples were centrifuged at 14,500*g* for 20 min at 4 °C and protein-containing supernatant was transferred to a fresh tube. RIPA lysed samples quantification was then performed using a Pierce BCA assay (Thermo Scientific) and equal amounts of sample was resuspended in required volumes of 4× Laemmli sample buffer (Bio-Rad, 1610747) and boiled at 95 °C for 10 min. To immunoblot the proteins, samples were separated by size on pre-cast polyacrylamide gradient gels (Bio-Rad, 4561084) and transferred onto 0.2-µm nitrocellulose membranes (Bio-Rad). Efficient transfer and correct gel loading were verified by Ponceau S staining before 1 h blocking of membranes with 5% milk (wt/vol) diluted in TBS supplemented with 0.1% Tween-20 (vol/vol; TBST). Primary antibodies were diluted in 5% milk (wt/vol, TBST) and incubated with membranes overnight at 4 °C. This was then followed by three washes with TBST followed by 1 h incubation with HRP-conjugated secondary antibody. Secondary antibody binding was visualized using Amersham ECL Prime western blotting detection reagent (Cytiva) and imaged on an Amersham Imager 680 blot and gel imager (Cytiva).

### RNA extraction

Total RNA from the tissues was extracted in a bulk way by bead disruption in 800 µl of TRIzol reagent (Invitrogen) using a TissueLyser system (Qiagen) followed by further homogenization using a QIAshredder kit (Qiagen), according to the manufacturer’s instructions. The homogenized tissue in TRIzol was then mixed with 160 µl of chloroform (Sigma) and vortexed for 15 s, then centrifuged at 14,500*g* at 4 °C for 30–45 min. The top aqueous phase containing RNA was then column-purified using an RNAeasy Mini Kit (Qiagen) and subjected to DNase treatment according to the manufacturer’s instructions. RNA concentration was determined using a NanoDrop ND-1000 UV–vis spectrophotometer at a wavelength of 260 nm.

For extraction of total RNA from cells, six-well plates were scraped in 800 µl of TRIzol reagent (Invitrogen), mixed with 160 µl of chloroform (Sigma), vortexed and centrifuged as stated above. The aqueous phase was then transferred to a new tube and processed from step 2 onwards of the manufacturer’s instructions for the RNAeasy Mini Kit (Qiagen).

### cDNA synthesis and quantitative RT–PCR

To generate cDNA, total RNA was diluted in nuclease-free water to the same concentration across samples of the same experiment, and 1–5 µg was amplified using a SuperScript II reverse transcriptase kit (Invitrogen) combined with 1 µl of random hexamer primers (50 ng µl^−1^, Invitrogen), 1 µl dNTP mix (10 mM, Bioline) and made up to a final volume of 11 µl in nuclease-free water. The mixture was then added to a thermocycler for one cycle of 10 min at 25 °C, 50 min at 42 °C and 15 min at 70 °C. cDNA samples were then diluted at 10 ng µl^−1^ based on input RNA concentration.

mRNA expression analysis was carried out using real-time quantitative PCR (RT–qPCR) by way of amplification of cDNA using SYBR Green PCR Master Mix (Applied Biosystems) run on a CFX96 Real-Time PCR Detection system (Bio-Rad). RT–qPCR primers were selected from PrimerBank^69^ spanning exon–exon junctions. The relative gene expression in human cell lines was determined using the ΔΔCt method by measuring the RT–qPCR signal relative to the signal of the housekeeping gene *RPS14* and normalization to control samples. For mouse mRNA expression, the ΔΔCt method was again used, but the signal was measured relative to *GAPDH*.

For the mouse fibrosis experiments, tissues were homogenized in TRIzol and the cDNA was synthesized using SuperScript III reverse transcriptase (Thermo Fisher). Real-time PCR was performed using the PowerUp SYBR Green Master Mix (Applied Biosystems). Gene expression analysis was performed using the indicated primers. The results were then normalized using the housekeeping gene *Gapdh*, *Actin b* or *Hprt*. The primer pairs used are presented in Supplementary Table 2.

### RNA-seq and GSEA

The total RNA extracted and purified from tissues or cell extraction was analysed on a 2100 Bioanalyzer (Agilent) using an RNA 6000 Nano Kit (Agilent) to verify the RNA purity and integrity before library preparation. RNA from tissue samples with an RNA integrity number (RIN) corresponding to a ratio of the 18S-to-28S rRNA peaks on the bioanalyser trace of less than 3 were not submitted for library processing. Library preparation to generate cDNA was performed by the MRC–LMS genomics core facility with 200 ng of starting RNA using the NEBNext Poly(A) mRNA magnetic isolation kit (NEB, E7490) to isolate mRNA from the total RNA sample. Purified samples were then processed using the NEBNext Ultra II Directional RNA Library Prep Kit for Illumina (NEB, E7760). Libraries were then assessed on a 2100 bioanalyzer and concentration was determined using a Qubit fluorometer and the Qubit dsDNA HS assay kit (Thermo Scientific). Indexed libraries were then run on two lanes of a NextSeq 2000 sequencer (Illumina), with >10 million single-end 75-bp reads being generated per sample. Human RNA-seq reads were assessed for quality using FASTQC and then aligned to human genome hg19 by Tophat (v. 2.0.11) using ‘-library-type- fr-firststrand’ parameters along with gene annotation from Ensembl (v.67). GSEA was carried out on the differential expression of vehicle and drug-treated aged tissues using Wald statistics parameters in DESeq2 and all curated gene sets in MSigDB.

### Live-cell microscopy

To analyse the live-cell induction of apoptosis, cells were incubated with IncuCyte caspase-3/7 reagent (1:500, Essen Bioscience) following reverse transfection with senolytic siRNAs or drug treatment. Four images per well of a 96-well plate were collected every 2 h for 3–4 days using a ×10 objective on an IncuCyte microscope, and fluorescence images were analysed with IncuCyte Zoom software (Essen Bioscience).

### Druggable genome siRNA screening and siRNA transfection

Druggable genome siRNA libraries were purchased from Qiagen (Human Druggable Genome siRNA Set V4.1, 2 siRNA per gene) and Dharmacon (siGenome human druggable genome, four siRNA per gene). Individual siRNAs were purchased from the siGenome reagent family of Dharmacon (Horizon Discovery) and came lyophilized in tube format or coated onto 96-well plates. Before transfection, plates containing 0.1 nM of lyophilized siRNA were resuspended in 100 µl of nuclease-free water and 3.6 µl of siRNA aliquoted into daughter plates. For large-scale libraries, daughter plates were aliquoted using a laboratory automation workstation (Biomek NX^P^, Beckman Coulter). Transfection mix containing 0.2 µl of DharmaFECT 1 with 17.4 µl of DMEM only or 0.4 µl DharmaFECT 1 with 17.2 µl of DMEM was added to daughter siRNA plates for IMR90 ER:RAS or IMR90 experiments, respectively. To reverse-transfect cells, 100-µl suspensions of proliferating or senescent cells in medium with DMEM supplemented with 10% FBS only were added to plates with combined transfection mix and siRNA (final siRNA concentration 30 nM). After 18 h, when cells had been allowed to adhere, the medium was replaced with DMEM supplemented with 0.5% (wt/vol) FBS and 1% antibiotic–antimycotic solution. Plates were then fixed in 4% PFA (wt/vol) 72 h after a medium change, to then be processed for quantitative IF. For analysis of mRNA, the protocol was scaled to a six-well-plate format and cells were collected by the addition of TRIzol RNA isolation reagent (Invitrogen) to the well followed by scraping and collection. Information about the siRNAs used in this study is provided in Supplementary Table 3.

### B-score normalization analysis

To analyse the siRNA screen, cell counts were normalized by B-score using the R package CellHTS2 (10.18129/B9.bioc.cellHTS2)^70^. Cell count normalization was performed using the plate-averaging method and on separate batches for control and senescent cells, in addition to a separate normalization performed for each batch of plate transfections.

### Enzyme-linked immunosorbent assay

For the detection of secreted factors in conditioned media of IMR90 ER:RAS cells, 100 µl of medium (DMEM supplemented with 0.5% (wt/vol) FBS and 1% antibiotic–antimycotic solution) incubated with cells and inhibitors for 48–72 h was collected and filtered using a 0.2-µm cellulose acetate membrane (Gilson). Filtered samples were then subject to an enzyme-linked immunosorbent assay (ELISA) kit according to the manufacturer’s instructions (R&D: IL-6, DY206; IL-8, DY208; VEGF, DY293B; CXCL1, DY275; G-CSF, DY214; GM-CSF, DY215; CCL2, DY279; CCL20, DY360; LIF, DY7734). Cell numbers were calculated using high-throughput microscopy and used to normalize the levels of secreted factors.

### Proteostat assay

Relative levels of protein aggregates were measured using the PROTEOSTAT protein aggregation assay (ENZ-51023) according to the manufacturer’s instructions. Briefly, cells plated in a 96-well format and treated with the drug were incubated with PROTEOSTAT detection reagent for 15 min at r.t. and read on a FLUOstar Omega plate reader at 550 nm (ex.) and 600 nm (em.). The background was subtracted and intensity values normalized to cell counts from fixed DAPI-stained plates using high-throughput microscopy.

### Visualization of IpaJ effects on *N*-myristoylation with YnMyr and immunoblot analysis

In triplicate for each condition, IMR90 cells (controls, and cells transduced with IpaJ WT and IpaJ C64A constructs) were seeded in six-well plates and grown to 70–80% confluence. IMR90 control cells were incubated for 1 h with DMSO or 100 nM IMP1088. All conditions, including IpaJ variant-expressing cells, were thereafter metabolically labelled with 20 μM YnMyr for 18 h. The cells were then washed with PBS, collected by trypsinization, and the cell pellets were stored at −80 °C until further analysis. The cell pellets were lysed and the YnMyr-labelled proteins were functionalized with fluorescent capture reagent, then resolved by fluorescence scanning after separation on 15% (wt/vol) SDS–PAGE gels as previously described^45,50^. Immunoblotting was performed on ARF1, ARL1, PPM1β and TUBA, then read out on a Li-Cor Odyssey CLx system using IRDye 800CW-functionalized secondary antibodies. Fluorescence intensities were quantified by ImageJ and normalized to the TUBA loading control.

### Statistics and reproducibility

Statistical analyses were performed and plotted using GraphPad Prism 9 software. Details of the test used are given in the corresponding figure legends and the source data. Statistical analysis was performed using either an unpaired two-tailed *t*-test with Holm–Sidak multiple comparison correction or with ordinary one- or two-way ANOVA with Dunnett’s or Tukey’s multiple comparison correction. Tumour growth curves were analysed using Repeated Measure (RM) two-way ANOVA with Greenhouse–Geisser correction and Dunnett’s correction. *P* values and adjusted *P* values are shown for values lower than *P* = 0.1. *P* values and adjusted *P* values for other comparisons and per experiment statistical test details are available in the source data.

No statistical method was used to predetermine sample size. For the bleomycin-induced fibrosis experiment, two mice in the vehicle-treated group died before the end of the experiment and were not included in any analysis. For the WD experiment, a mouse of the WD + vehicle group died before the end of the experiment and was not included in any analysis. RNA isolation from one mouse liver of the WD + NMTi group failed and it could not be included in subsequent RT–qPCR analysis. Three sections from Fig. 8h were excluded due to poor section processing. No further data were excluded from analyses. For in vivo studies, mice were randomized to treatment groups. Cell-culture experiments were not randomized. Histologic analysis of Masson’s trichrome and haematoxilin & eosin (H&E) staining for the mouse fibrosis experiment was examined first in a blinded fashion and in a second round in an unblinded fashion. Tumour measurements were taken blindly and independently by the researcher or the animal technicians in the mouse facility. For the HDTVI and WD experiments, staining and analysis were performed in a blinded fashion. Investigators were not blinded during the other experiments.

### Reporting summary

Further information on research design is available in the Nature Portfolio Reporting Summary linked to this Article.

## Online content

Any methods, additional references, Nature Portfolio reporting summaries, source data, extended data, supplementary information, acknowledgements, peer review information; details of author contributions and competing interests; and statements of data and code availability are available at 10.1038/s41556-023-01287-6.

### Supplementary information

Supplementary Information Supplementary Figs. 1–17 with their legends, Source numerical data for Supplementary figures and source gel data for Supplementary figures.

Reporting Summary

Supplementary Table Contains Supplementary Tables 1–3 as different sheets in the Excel file

### Source data

Source Data Fig. 1 Statistical source data.

Source Data Fig. 2 Statistical source data.

Source Data Fig. 3 Statistical source data.

Source Data Fig. 4 Statistical source data.

Source Data Fig. 5 Statistical source data.

Source Data Fig. 6 Statistical source data.

Source Data Fig. 6 Unprocessed western blots.

Source Data Fig. 7 Statistical source data.

Source Data Fig. 8 Statistical source data.

Extended Data Fig. 1 Statistical source data.

Extended Data Fig. 2 Statistical source data.

Extended Data Fig. 3 Statistical source data.

Extended Data Fig. 4 Statistical source data.

Extended Data Fig. 4 Unprocessed western blots.

Extended Data Fig. 5 Statistical source data.

Extended Data Fig. 6 Statistical source data.

Extended Data Fig. 7 Statistical source data.

Extended Data Fig. 8 Statistical source data.

Extended Data Fig. 9 Statistical source data.

Extended Data Fig. 10 Statistical source data.

## Data Availability

RNA-seq data have been deposited in the Gene Expression Omnibus (GEO) under accession codes GSE224070, GSE224071 and GSE224069. All other data supporting the findings of this study are available from the corresponding author upon reasonable request. Source data are provided with this paper.
