# Supplementary material for: COPI vesicle formation and N-myristoylation are targetable vulnerabilities of senescent cells
Source: Nat Cell Biol. 2023 Nov 27;25(12):1804–20. doi: 10.1038/s41556-023-01287-6 (PMC10709147; doi:10.1038/s41556-023-01287-6)
Supplement: Supplementary file 1 — Supplementary Figs. 1–17 with their legends, Source numerical data for Supplementary figures and source gel data for Supplementary figures. [file 41556_2023_1287_MOESM1_ESM.pdf]

nature cell biology

Article

https://doi.org/10.1038/s41556-023-01287-6

COPI vesicle formation and *N*-myristoylation are targetable vulnerabilities of senescent cells

In the format provided by the authors and unedited

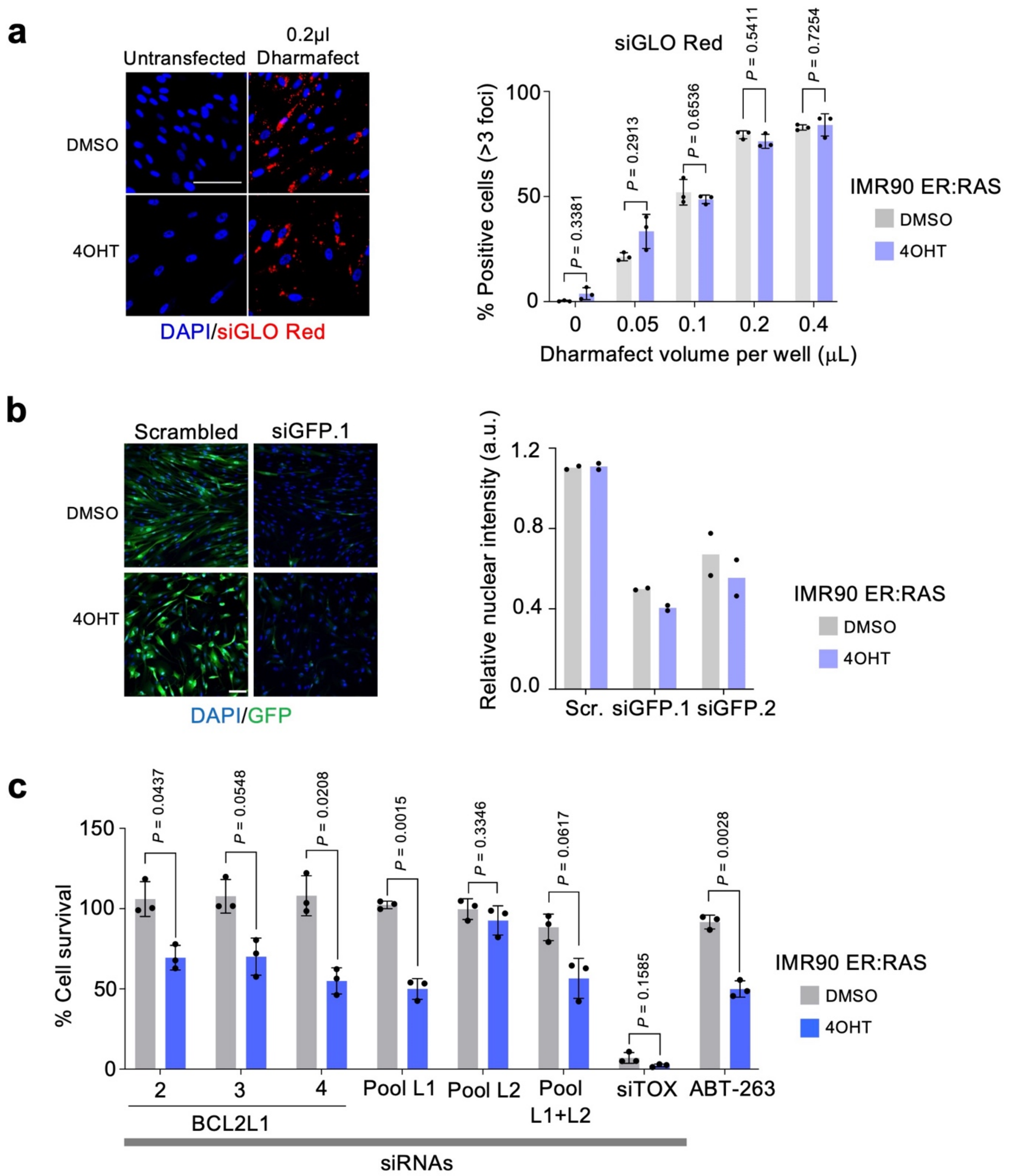

Supplemental Figure 1. Setting up RNAi screens for senolytic targets.a, Quantification of the percentage of cells with 3 or more cytoplasmic foci of fluorescent siGLO Red (Right) in control (DMSO) or senescent (4OHT) IMR90 ER:RAS cells 72h after reverse transfection at day 6 after senescence induction. Cells were transfected with varying volumes of Dharmafect 1 per well. Representative IF images are shown (left). (n=3). Scale bar, 100µm. **b,** Quantification of GFP nuclear intensity (right) relative to un-transfected control for control (DMSO) or senescent (4OHT) IMR90 ER:RAS cells 72h after reverse transfection with GFP siRNAs on day 6 post-

senescence induction cells. (n=2) Representative IF images shown (left). Scale bar, 100µm. **c,** Survival of control (DMSO) or senescent (4OHT) IMR90 ER:RAS cells 72h after reverse transfection with the indicated siRNAs on day 6 post-senescence induction. Pools of 4 individual siRNAs targeting BCL2L1 (pool L1), BCL2L2 (pool L2), or 4 siRNAs each against BCL2L1 and BC2L2 (pool L1+L2) were used. Treatment of cells with 1µM ABT-263 for 72 h starting 6 days after senescence induction was used as a senolytic control (ABT-263). (n=3). Data throughout the figure is represented as mean±SD where applicable. Statistical tests throughout figure were performed using unpaired, two-tailed, student's t-test. N represents independent experiments.

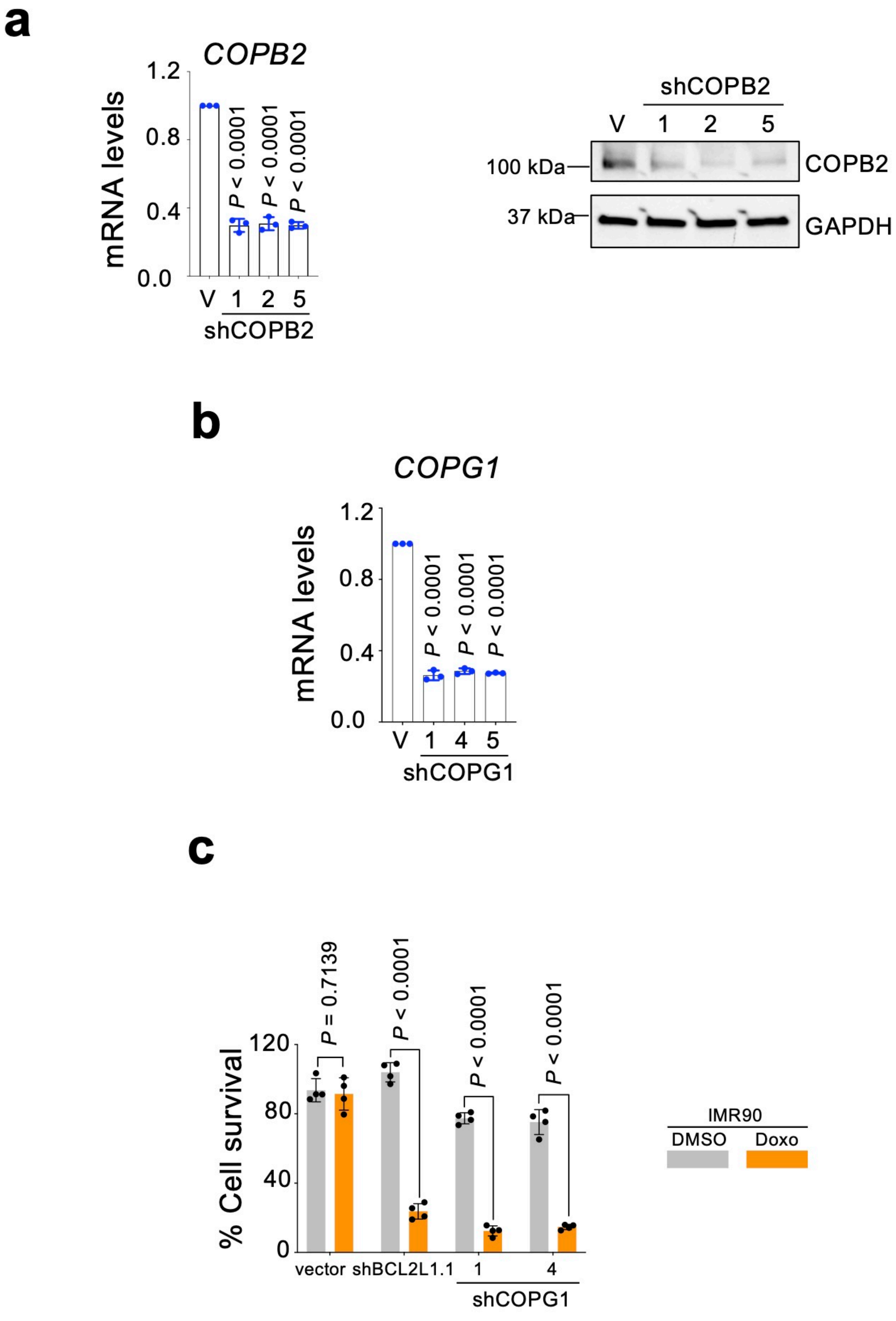

Supplemental Figure 2. Knockdown of COPB2 and COPG1 using shRNAs. a, Levels of COPB2 after knockdown with three independent shRNAs. (Left) qRT-PCR

(n=3) One-way ANOVA. (Right) Panel showing immunoblot representative of two independent experiments. Immunoblot of GAPDH is included as a loading control. **b,** Levels of COPG1 after knockdown with three independent shRNAs. (n=3). One-way ANOVA. **c,** Quantification of cell survival of control (DMSO) and doxorubicin-induced senescent (Doxo) cells. (n=4) Unpaired two-tailed Student's t-test. Data represented as mean±SD throughout figure. N represents independent experiments.

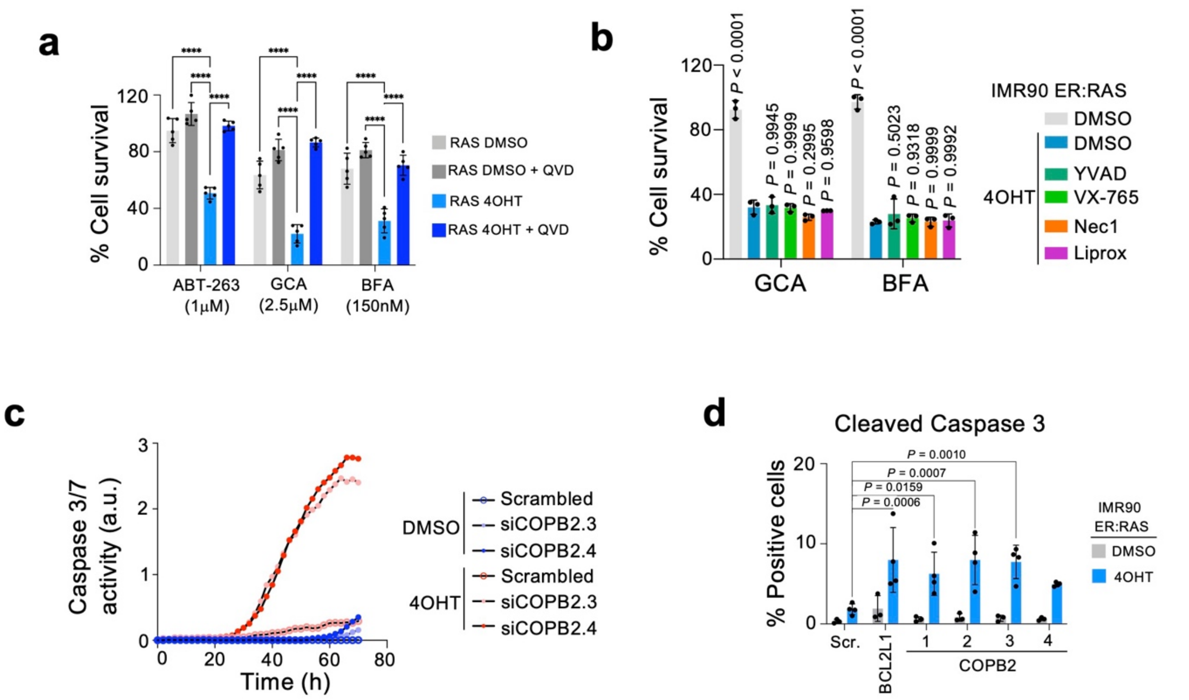

Supplemental Figure 3. COPI inhibition causes apoptosis in senescent cells. a, Quantification of percentage cell survival of control (DMSO) or senescent (4OHT) IMR90 ER:RAS cells treated in parallel with 20 μM of pan-caspase inhibitor (Q-VD-OPh) and either 1μM ABT-263, 2.5μM golgicide A (GCA) or 150nM brefeldin A (BFA) for 72h. (n=5). Ordinary Two-way ANOVA**. b,** Quantification of percentage cell survival of senescent (4OHT) treated in parallel with inhibitors to inhibit pyroptosis (10μM Z-YVAD-FMK, 20μM VX-765), necroptosis (10μM Nec-1) or ferroptosis (1μM Liproxstatin) and either 2.5μM golgicide A (GCA) or 150nM brefeldin A (BFA) for 72h. (n=3). Comparisons are to the corresponding senescent cells treated with DMSO (blue bars). Ordinary Two-way ANOVA. **c,** Caspase-3/7 activity in control (DMSO) or oncogene-induced senescent (4OHT) cells after reverse transfection with COPB2 siRNAs 6 days after senescence induction (n=2). **d,** Quantification of cells positive for cleaved caspase 3 as assessed by IF analysis (n=4). Unpaired, two-tailed, Student's t-test. Data is represented throughout the figure as mean ± SD. N represents number of independent experiments.

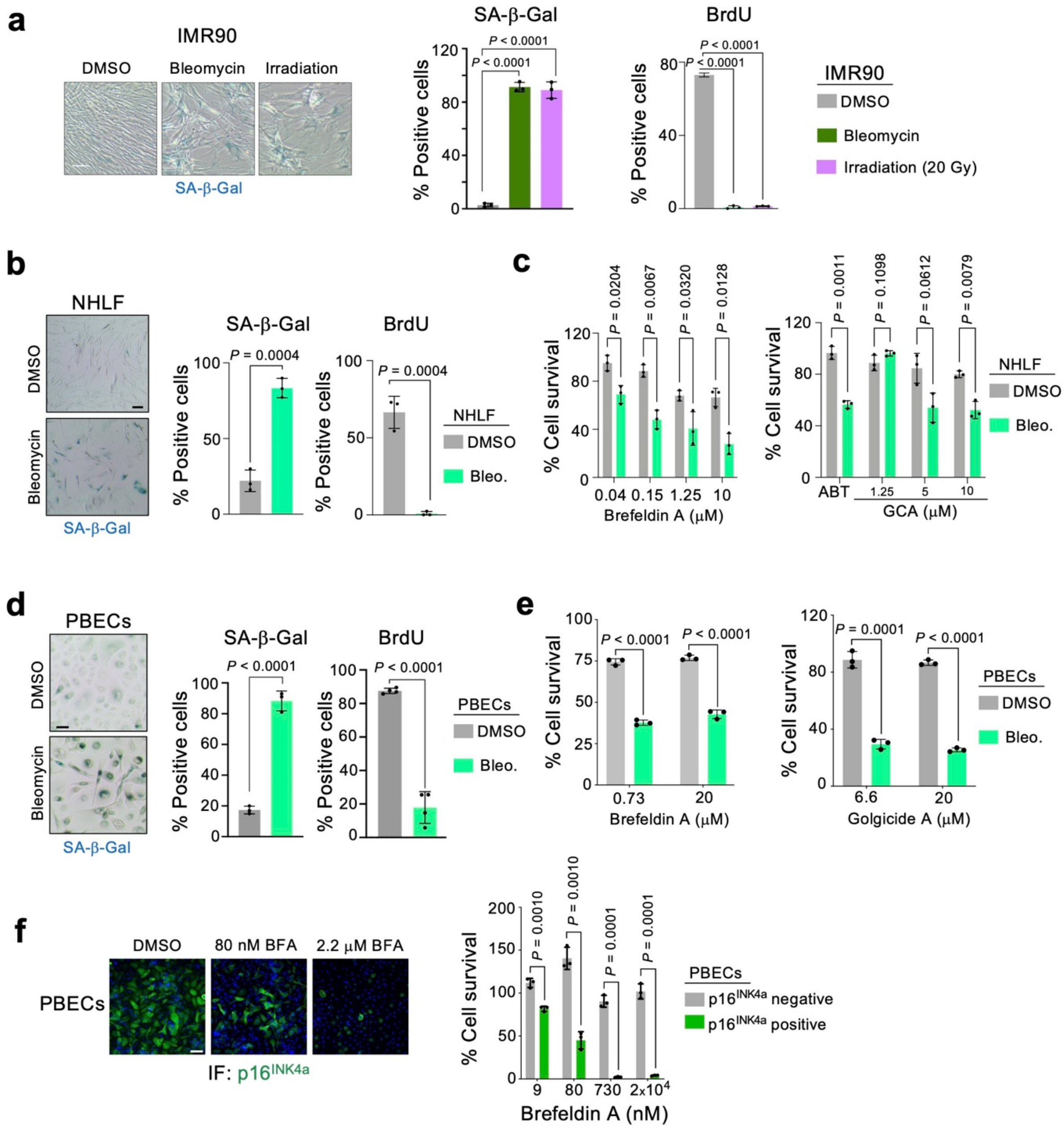

Supplemental Figure 4. GBF1 inhibitors are senolytic. a, Induction of senescence by bleomycin and irradiation in IMR90 cells. Quantification of the percentage of IMR90 cells positive for SA-β-gal staining (middle) or BrdU incorporation (right) 6 days after treatment of cells with 33μM bleomycin or 20Gy Irradiation. (n=3). Representative image of SA-β-gal staining shown (left). Scale bar, 100μm. One-way ANOVA. **b,** Bleomycin-induced senescence in NHLF cells. Quantification of the percentage of NHLF cells positive for SA-β-gal staining (middle) or BrdU incorporation (right) 6 days after treatment of cells with 50 μg/ml bleomycin (n=3). Representative image of SA-β-gal staining shown (left). Scale bar, 50μm. Unpaired, two-tailed Student's t-test. **c,** Percentage cell survival in either control (DMSO) or bleomycin-treated (Bleo) NHLF

cells 72h after treatment on day 7 with brefeldin A (left) or golgicide A (GCA, right). (n=3). Unpaired, two-tailed. Student's t-test. **d,** Bleomycin-induced senescence in PBECs. Quantification of the percentage of PBECs staining positive for SA-β-gal activity (middle) or BrdU incorporation (right) 6 days after treatment with 100ng/ml bleomycin (n=3). Representative image of SA-β-gal staining shown (left). Scale bar, 50μm. Unpaired, two-tailed. Student's t-test. **e,** Percentage cell survival in either control (DMSO) or bleomycin-treated (Bleo) PBECs 72h after treatment on day 7 with brefeldin A (left) or golgicide A (GCA, right). (n=3). Unpaired, two-tailed. Student's t-test.. **f,** Quantification of percentage cell survival in either PBECs staining negative ($p16^{INK4a}$ negative) or positive ($p16^{INK4a}$ positive) for $p16^{Ink4a}$ 72h after treatment with brefeldin A (n=3). Representative images of $p16^{INK4a}$ IFs are shown (left). Unpaired, two-tailed. Student's t-test. Data is represented throughout the figure as mean±SD. N represents number of independent experiments.

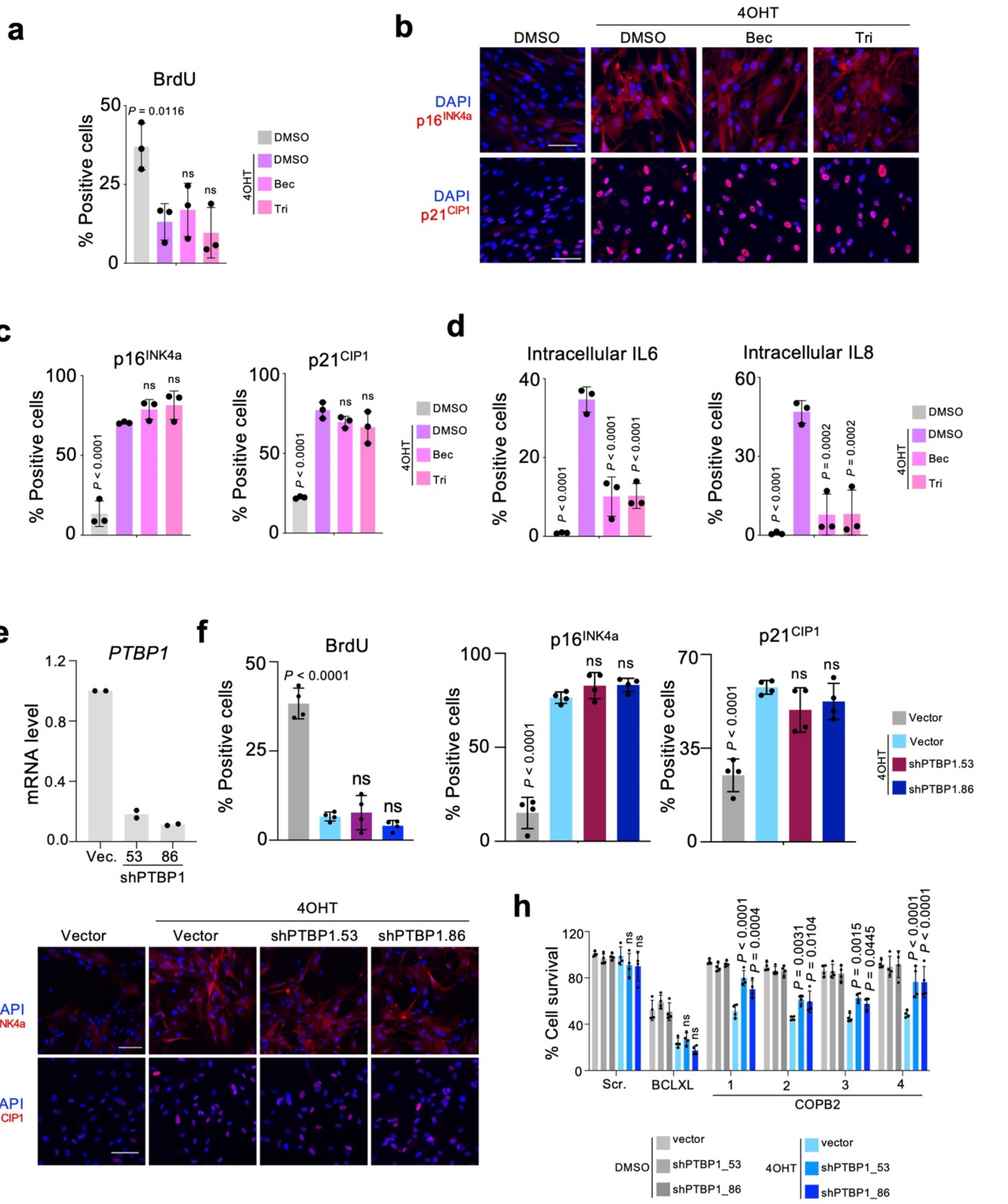

Supplemental Figure 5. Glucocorticoid treatment or PTBP1 depletion downregulate SASP without preventing the growth arrest associated with senescence. a Percentage of IMR90 ER:RAS cells positive for BrdU incorporation on day 6 post senescence induction. Cells were treated on day 4 with 10 µM beclomethasone (Bec) or on day 0 with 10 µM triamcinolone (Tri) (n=3). One-way ANOVA. **b,** Representative immunofluorescence images of IMR90 ER:RAS cells treated with beclomethasone or triamcinolone, fixed and stained 6 days post-induction. Scale Bar, 100µm. **c,** Quantification of percentage cells positive for p16^{INK4a}

(left) and p21^{CIP1} (right) on day 6 after senescence induction (n=3). Data represented as mean±SD. One-way ANOVA.**d,** Percentage of cells positive for IL6 (Left) and IL8 (Right) on day 10 after senescence induction (n=3) One-way ANOVA, Dunnett's correction. **e,** Relative mRNA levels of PTPB1 on day 4 following transduction with shRNAs. (n=2). p21^{CIP1}**. f,** Percentage of cells positive for BrdU incorporation (left), p16^{INK4a} (middle), or p21^{CIP1} (right) on day 6 after senescence induction (n=4). Doxycycline was added on day 0 to induce shRNAs. One-way ANOVA, Dunnett's correction. **g,** Representative IF images of cells from the experiment described in e-f. Scale Bar, 100μm. **h,** SASP inhibition caused by the knockdown of PTBP1 prevents the senolysis induced by COPB2 depletion. Quantification of cell survival of senescent (4OHT) and control (DMSO) IMR90 ER:RAS cells infected with the indicated shRNAs (n=4). Data represented as mean±SD. Statistical comparisons of 4OHT Vector vs. 4OHT shPTBP1 shRNAs are shown. Two-way ANOVA. Data throughout the figure is represented as mean ± SD. N throughout figure represents independent experiments.

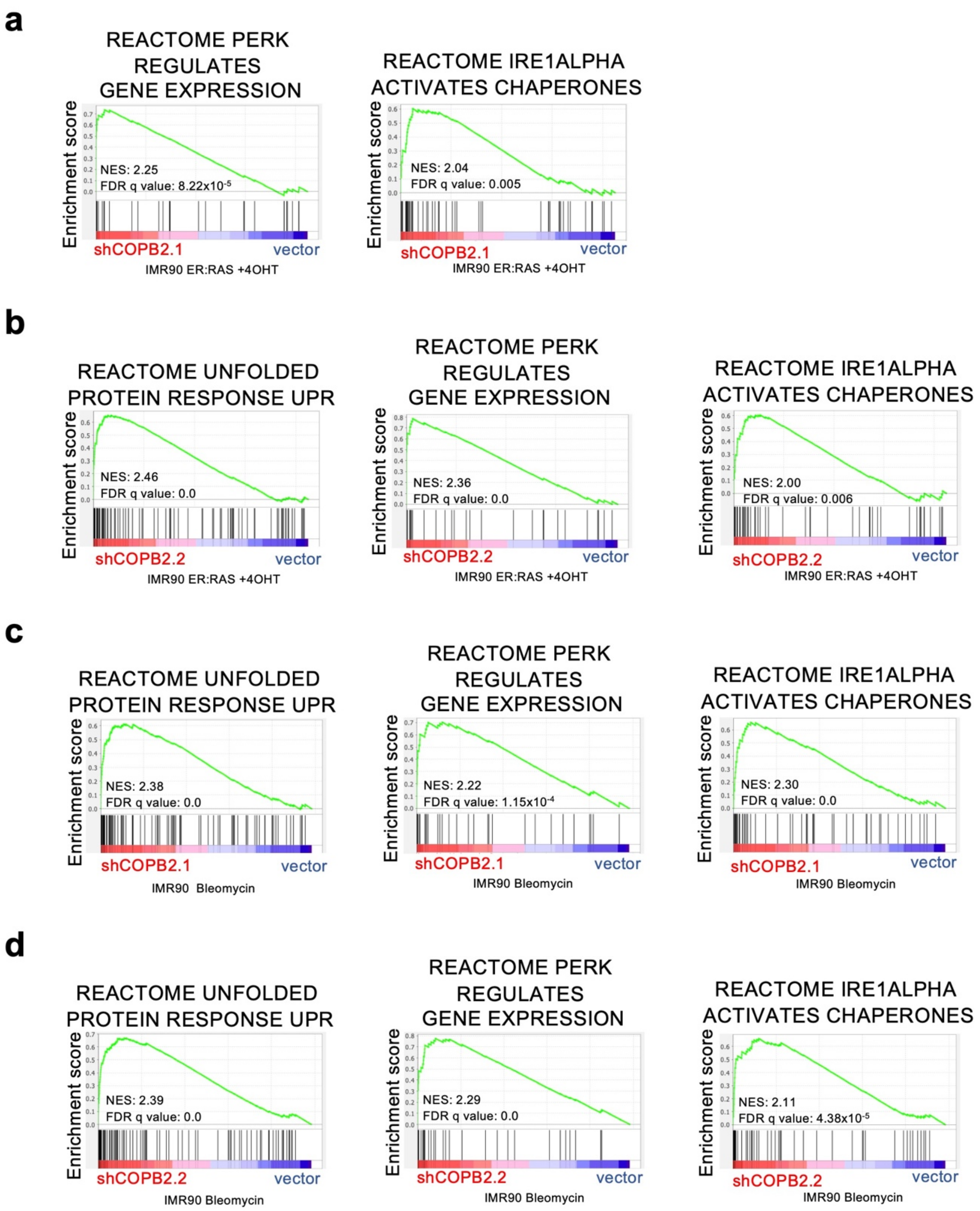

Supplemental Figure 6. COPB2 depletion activates the unfolded protein response. a-b, GSEA plot showing enrichment of the indicated signatures after COPB2 knockdown with either shCOPB2.1 (a, n=3) or shCOPB2.2 (b, n=3) in senescent IMR90 ER:RAS cells. **c-d,** GSEA plot showing enrichment of the indicated signatures after COPB2 knockdown with either shCOPB2.1 (a, n=3) or shCOPB2.2 (b, n=3) in IMR90 cells undergoing bleomycin-induced senescence. NES, normalized enrichment score; FDR, false discovery rate. N throughout figure represents independent experiments.

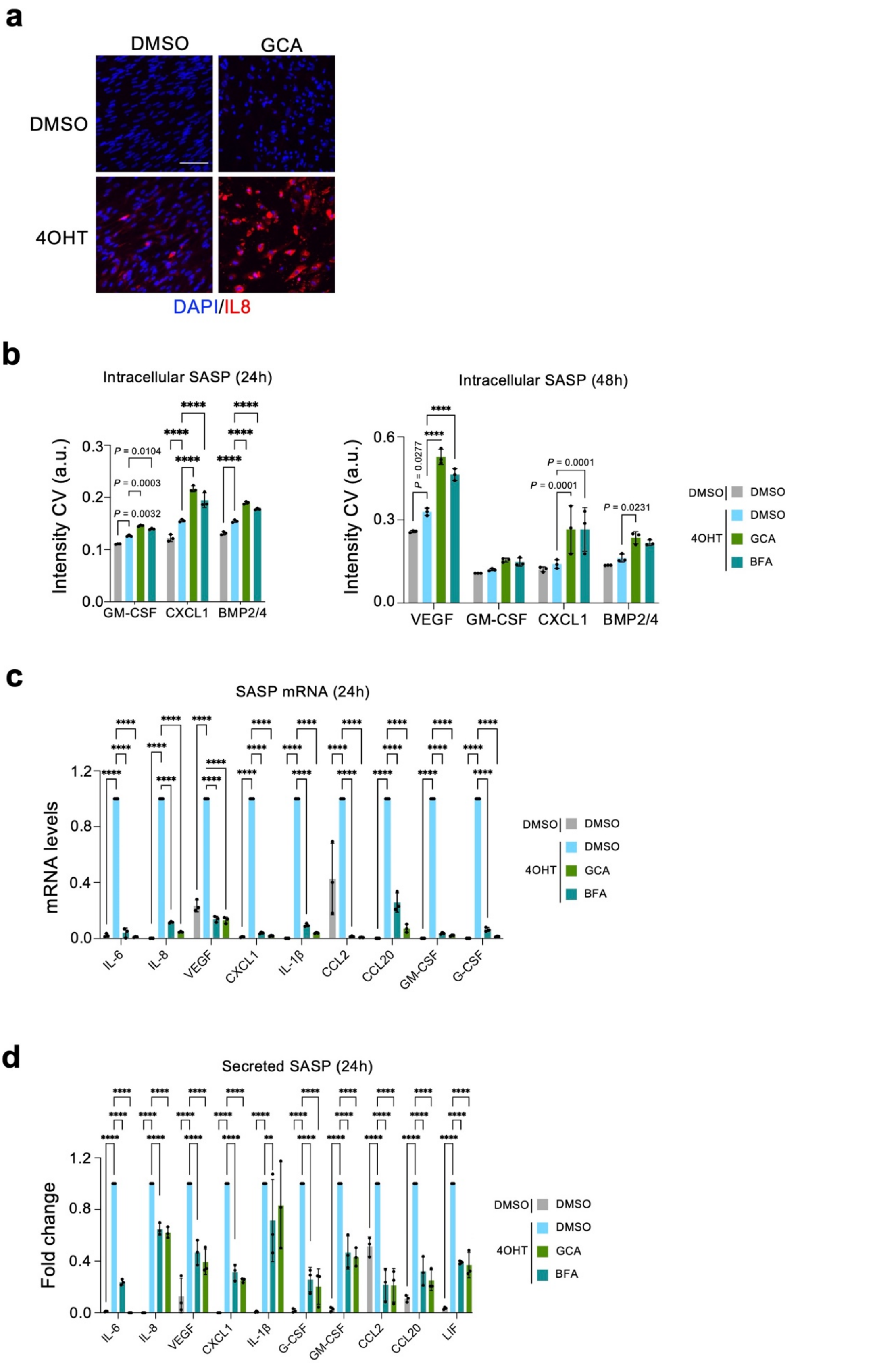

Supplemental Figure 7. Effect of GBF1 inhibitors on SASP production, secretion, and intracellular accumulation. a, Representative IF images showing IL8

staining of either control (DMSO) or senescent (4OHT) IMR90 ER:RAS cells, 48h after treatment with 1.25μM golgicide A (GCA). (n=3, quantification in Figure 4c) Scale bar, 100μm. **b,** Quantification of intracellular levels of SASP factors as assessed by IF at either 24h (left) or 48h (right) following treatment of IMR90 ER:RAS cells with either 1.25μM golgicide A (GCA) or 150nM brefeldin A (BFA), 7 days after senescence induction. (n=3). Two-way ANOVA. **c,** Quantification of mRNA levels for the indicated SASP factors 24h after treatment of IMR90 ER:RAS with either 1.25μM golgicide A (GCA) or 150nM brefeldin A (BFA), 7 days after senescence induction. (n=3). Two-way ANOVA. **d,** Fold change (relative to senescent cells) of secreted SASP levels 24h after treatment of day 7 IMR90 ER:RAS with either 1.25μM golgicide A (GCA) or 150nM brefeldin A (BFA). (n=3) as quantified by ELISA. Two-way ANOVA. Data throughout the figure are represented as mean±SD. N throughout figure represents independent experiments.

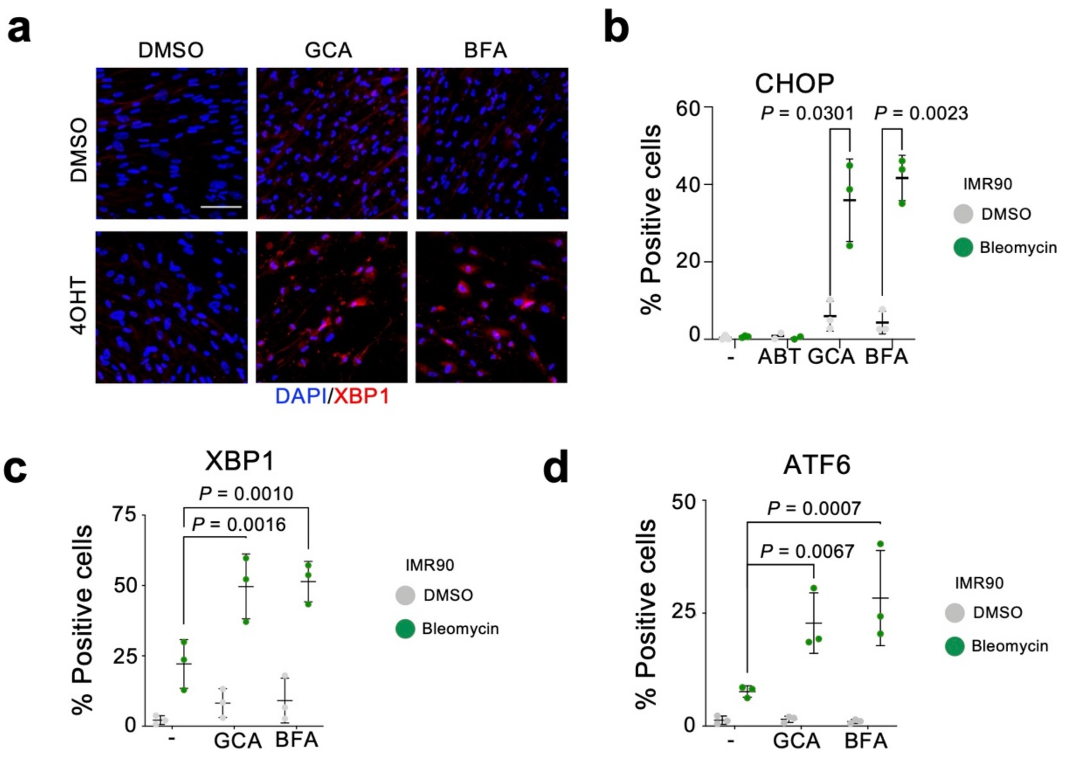

Supplemental Figure 8. COPI inhibition results in an unfolded protein response in cells undergoing bleomycin-induced senescence. a - b, Representative IF images of XBP1 staining (a) and quantification (b) in either control (DMSO) or senescent (4OHT) IMR90 ER:RAS cells treated with either 1.25µM golgicide A (GCA) or 150nM brefeldin A (BFA) for 48h. (n=3 (-), GCA & BFA; n=2, ABT-263). Unpaired, two-tailed, t-test. Scale bar, 100µm. **c-d,** Quantification of percentage of positive cells staining for nuclear XBP1 (c), or ATF6 (d) in control (DMSO) or therapy-induced senescent (Bleomycin) IMR90 cells treated on day 7 for 48h with either 1.25µM golgicide A (GCA) or 150nM brefeldin A (BFA) for 48h. (n=3) Unpaired, two-tailed Student's t-test. Data represented as mean ± SD. N represents number of independent experiments throughout figure.

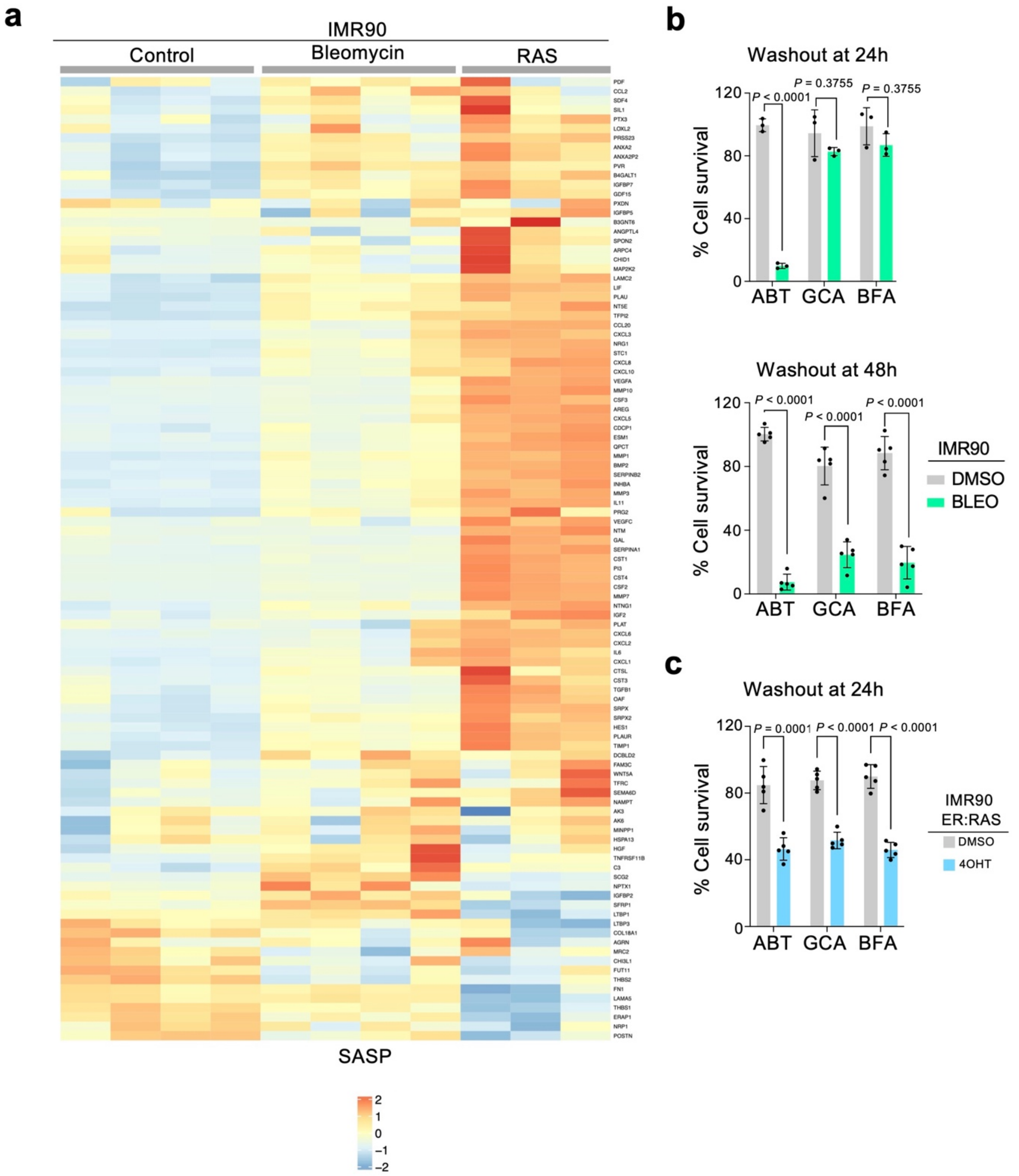

Supplemental Figure 9. Washout experiments with GBF1 inhibitors. a Heatmap showing expression of SASP factors in control (DMSO), oncogene-induced senescent (4OHT), and therapy induced senescence (BLEO) IMR90 cells. Data are shown as row z-score normalized. **b,** Percentage cell survival of control (DMSO) or bleomycin-treated (BLEO) IMR90 cells treated with 1μM ABT-263 (ABT), 2.5μM golgicide A (GCA) and 150nM brefeldin A (BFA) on day 7 post-induction for 24h (top, n=3) or 48h (bottom, n=5) followed by drug washout and fixation 72h after initial treatment. Unpaired, two-tailed Student's t-test**. c,** Percentage cell survival of control (DMSO) or

senescent (4OHT) IMR90 ER:RAS cells treated with 1μM ABT-263 (ABT), 2.5μM golgicide A (GCA) and 150nM brefeldin A (BFA) on day 7 post-induction for 24h, followed by drug washout and fixation 72h after initial treatment. (n=5). Unpaired, two-tailed Student's t-test. Data represented as mean ± SD throughout figure. N represents independent experiments.

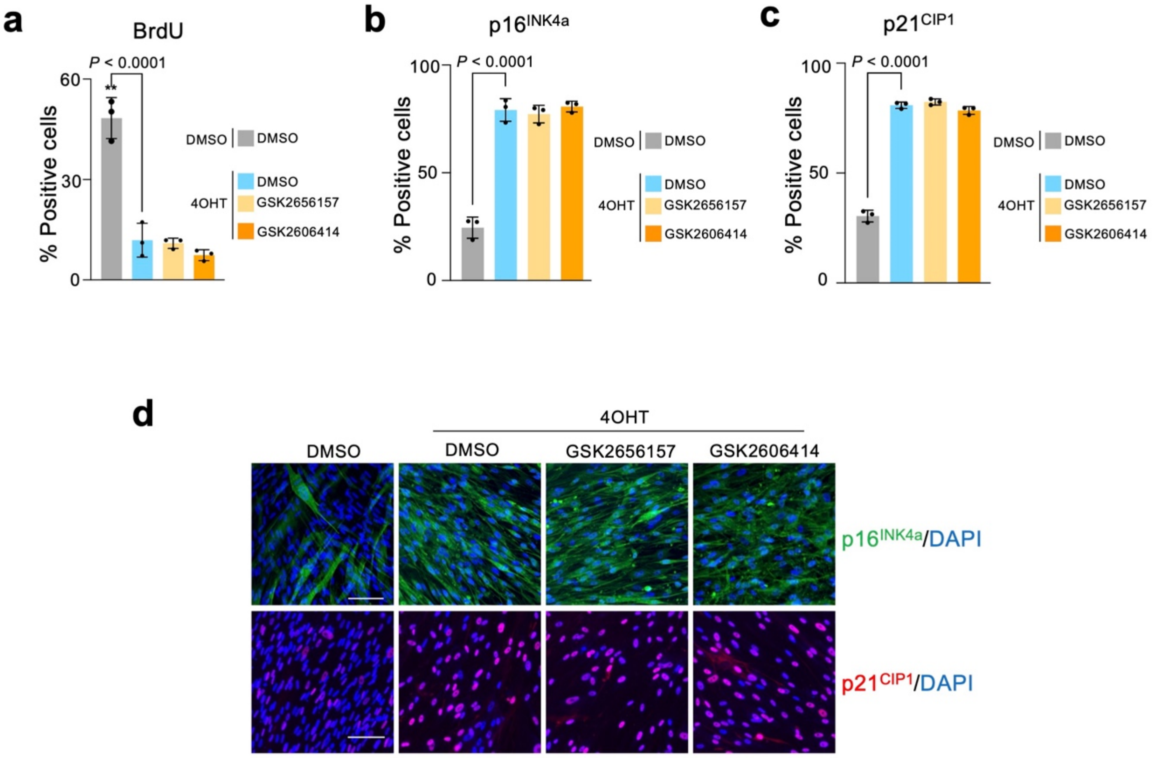

Supplemental Figure 10. Effect of PERK inhibitors on senescence. a-c, Quantification of BrdU (a), $p16^{INK4a}$ (b), and $p21^{CIP1}$ (c) staining by IF of either control (DMSO) or senescent (4OHT) IMR90 ER: RAS cells treated with either 1μM GSK2656157 or 1μM GSK2606414 on day 4 post senescence induction. Cells were fixed 6 days post senescence induction. (n=3) One-way ANOVA, Dunnett's Correction. Data represented as mean ± SD. **d,** Representative IF images of cells of the experiment described in a-c. Scale Bar, 100μm. N represents independent experiments.

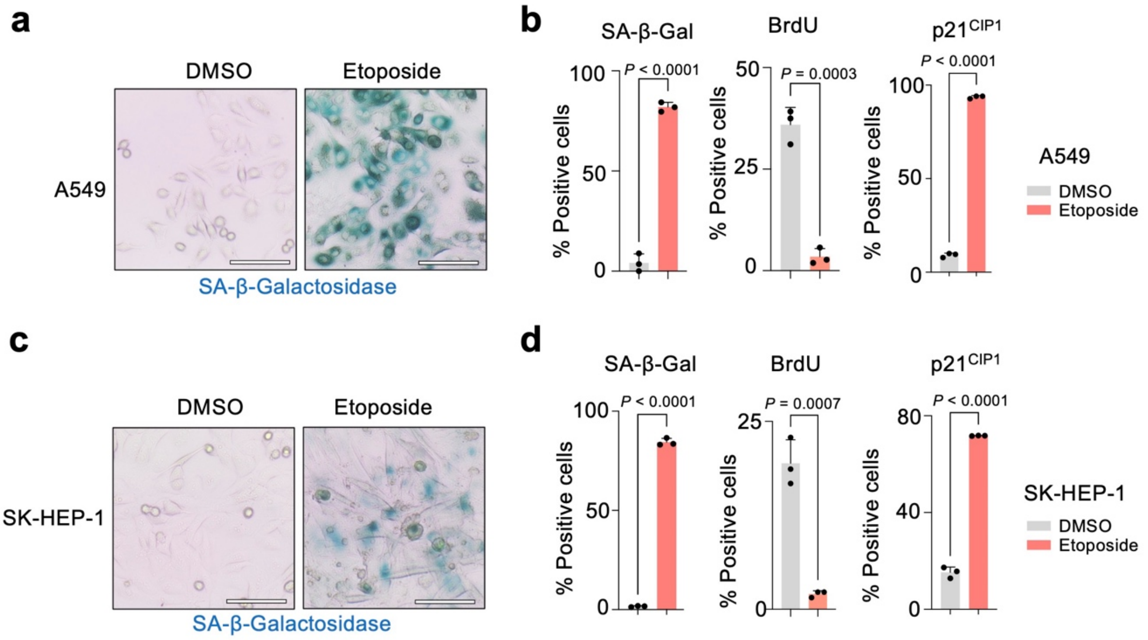

Supplemental Figure 11. Induction of senescence in A549 and SK-HEP-1 cells. **a,** Representative images of SA-β-gal staining in control (DMSO) or therapy-induced senescent (Etoposide) A549 cells 6 days after treatment with etoposide. Scale bar, 100μm. **b,** Quantification of percentage positive control (DMSO) or therapy induced senescent (etoposide) A549 cells for SA-β-gal staining (left), BrdU incorporation (middle) or $p21^{CIP1}$ staining s(right) on day 6. (n=3). Unpaired, two-tailed Student's t-test. **c,** Representative images of SA-β-gal staining in control (DMSO) or therapy-induced senescent (Etoposide) SK-HEP-1 cells 6 days after treatment with etoposide. Scale bar, 100 μm. **d,** Quantification of percentage positive control (DMSO) or therapy-induced senescent (Etoposide) SK-HEP1 cells for SA-β-gal staining (left), BrdU incorporation (middle) or $p21^{CIP1}$ staining s(right) on day 6. (n=3). Unpaired, Student's t-test. Data represented as mean ± SD. N represents independent experiments.

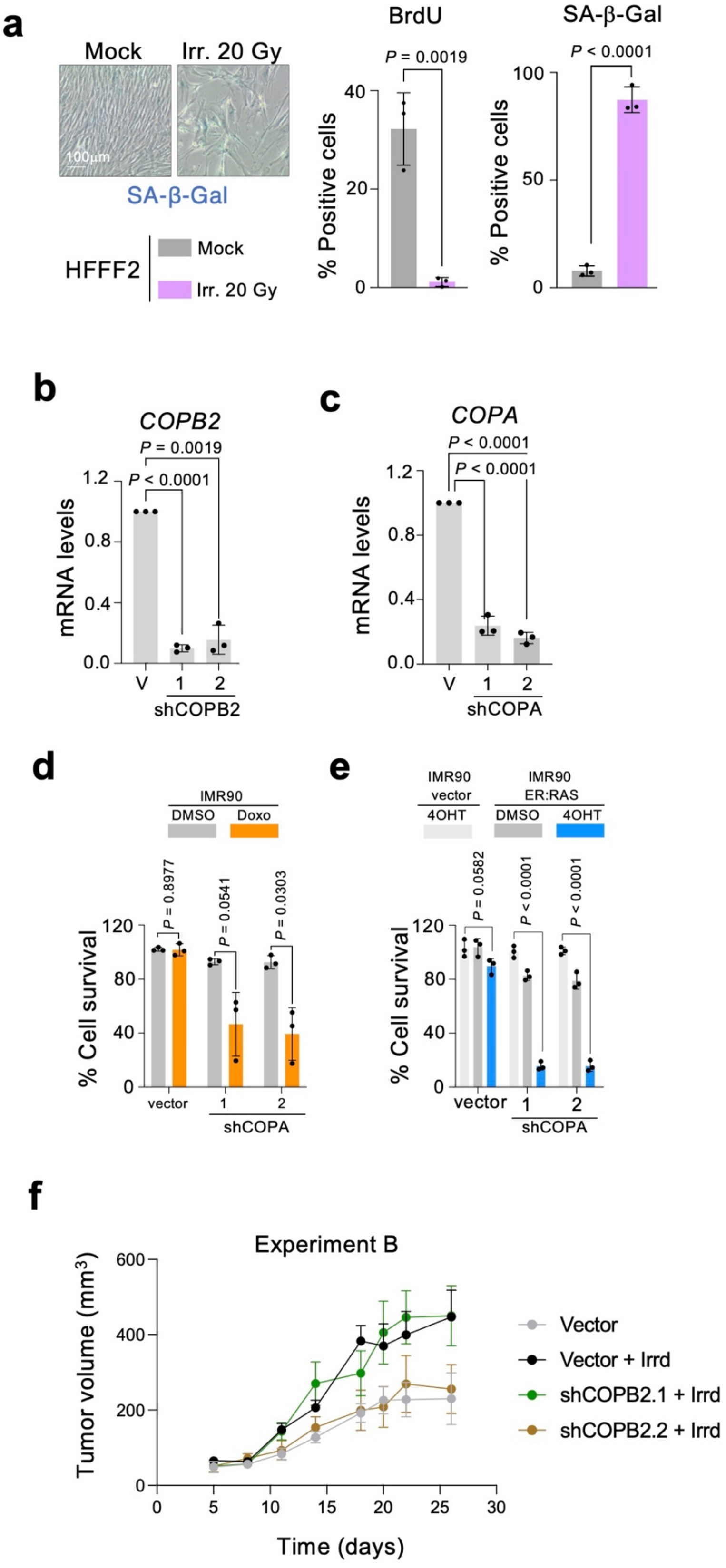

Supplemental Figure 12. Irradiation causes senescence in HFFF2 cells. a, Representative images of SA-β-gal staining (left) in either control (Mock) or irradiation-induced senescent (Irr. 20Gy) HFFF2 cells. Quantification of percentage cells positive

for BrdU incorporation (middle) or SA-β-gal staining (right) is shown. (n=3). Unpaired, two-tailed Student's t-test. **b-c,** Relative mRNA levels of COPB2 (b) and COPA (c) following transduction of HFFF2 cells with the corresponding shRNAs again each. (n=3) One-way ANOVA. **d,** Quantification of percentage cell survival in either control (DMSO) or therapy-induced senescent (Doxo). IMR90 cells following transduction with inducible shRNAs against COPA. shRNAs were induced 7 days after induction of senescence and cells fixed 10 days after doxycycline addition. (n=3) Unpaired, two-tailed Student's t-test. **e,** Senolytic activity of COPA depletion during OIS in IMR90 ER: RAS cells (n=3). shRNAs were induced 7 days after induction of senescence and cells fixed 10 days after doxycycline addition. (n=3) Unpaired, two-tailed Student's t-test**. f,** Tumor growth curves of a second experiment (Experiment B) showing the tumour volume monitored over time (IR=irradiation). Data represented as mean ± SEM for all mice in each group. (n=7 per group). This experiment and the one shown in Fig 5e (Experiment A) were pooled in the analysis shown in Extended Data Figure 5d. Data represented as mean ± SD throughout the figure unless otherwise stated. N represents independent experiments or mice throughout figure.

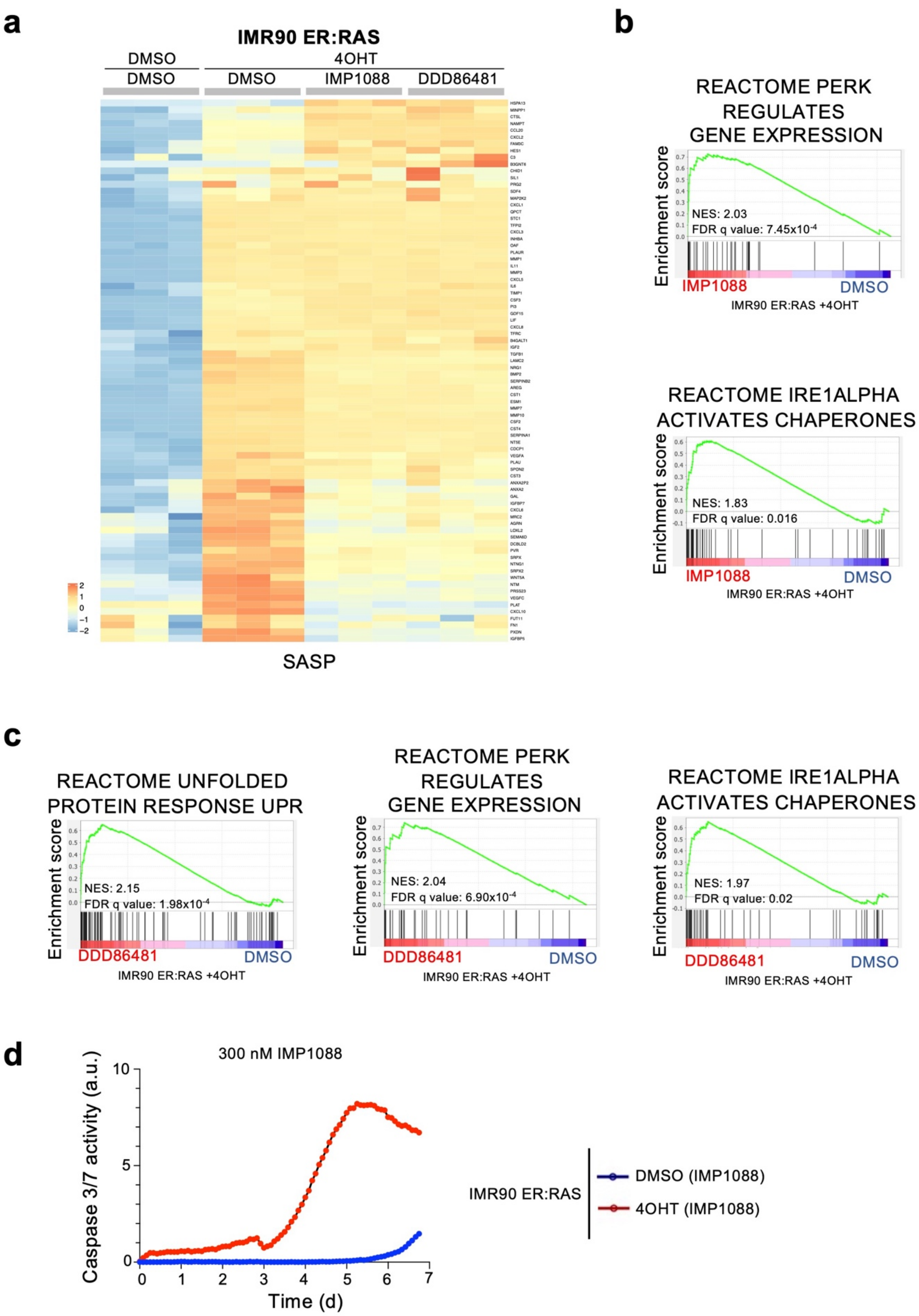

Supplemental Figure 13. NMT inhibitors are senolytic. a, Heatmap showing expression of SASP factors in either control (DMSO) or senescent (4OHT) IMR90 ER:RAS cells treated with 300nM IMP1088 or 1.5µM DDD86481. Data displayed as

row-z-score normalized. (n=3) **b-c,** GSEA plot showing enrichment of the indicated signatures in senescent cells treated with 300nM IMP1088 (b) or 1.5μM DDD86481 (c). NES, normalized enrichment score; FDR, false discovery rate. **d,** Caspase-3/7 activity in control (DMSO) or oncogene-induced senescent (4OHT) cells after treatment with DMSO or 300nM IMP1088. Measures start 7 days after senescence induction (n=2). Data represented as mean±SD. N represents independent experiments throughout figure.

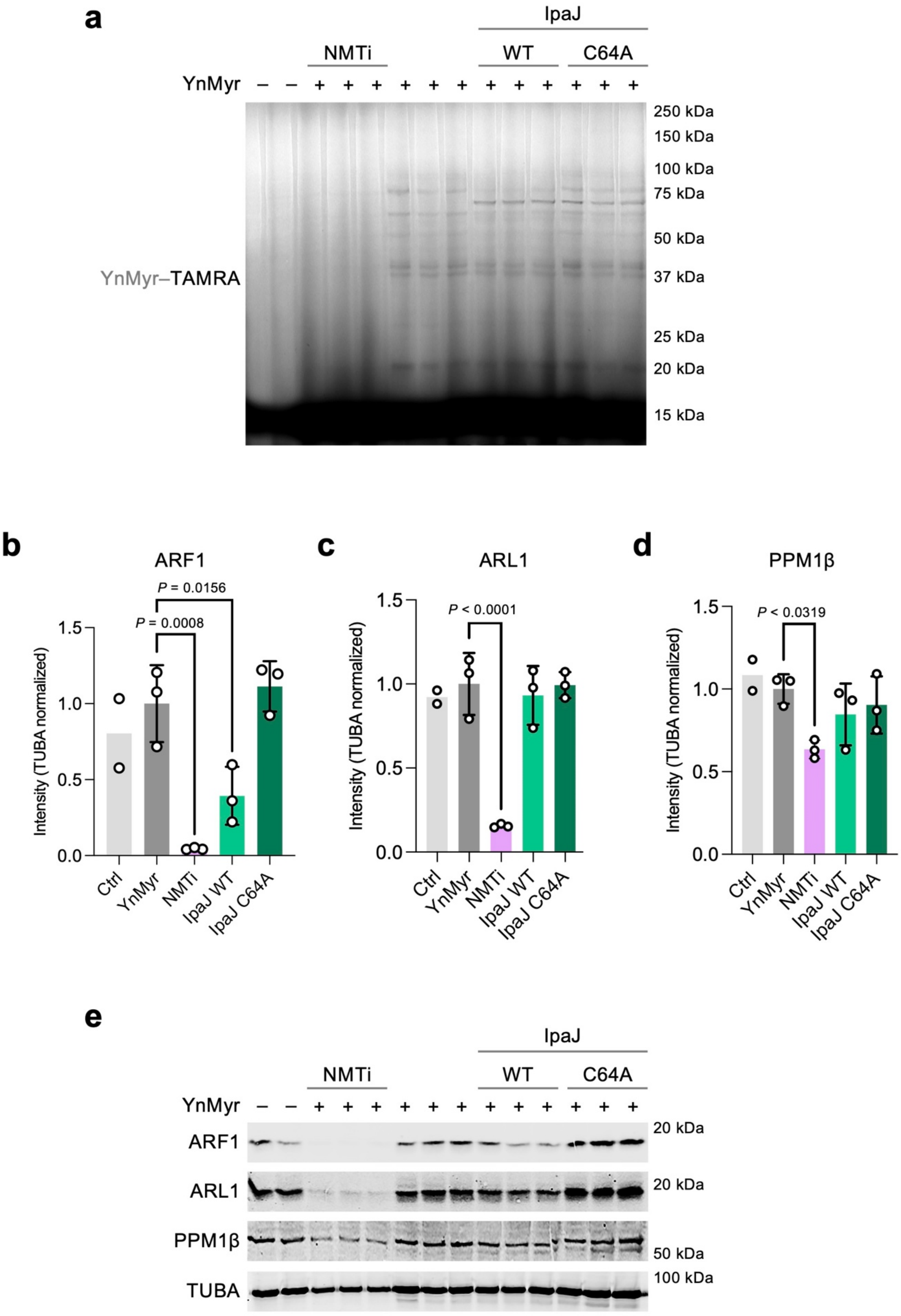

Supplemental Figure 14. Expression of IpaJ wt targets ARF1. a, In-gel visualization of the effect of NMTi (IMP-1088), IpaJ wild-type (WT), and inactive mutant (C64A) on N-myristoylation of proteins with YnMyr. Each lane shows an independent

sample, gel was run once. **b-d,** Densitometric intensity quantified of ARF1 (b), ARL1 (c), and PPM1b (d) normalized to TUBA levels. Ordinary One-way ANOVA, Dunnett's correction. (n=2 independent samples for controls, n=3 independent samples for all other groups). Each western blot includes the stated biological replicates per condition and was run once. Data represented as mean±SD. **e,** Immunoblots of ARF1, ARL1, PPM1B, and TUBA were used for the quantification shown in b-d. Immunoblot of TUBA is included as a sample processing control.

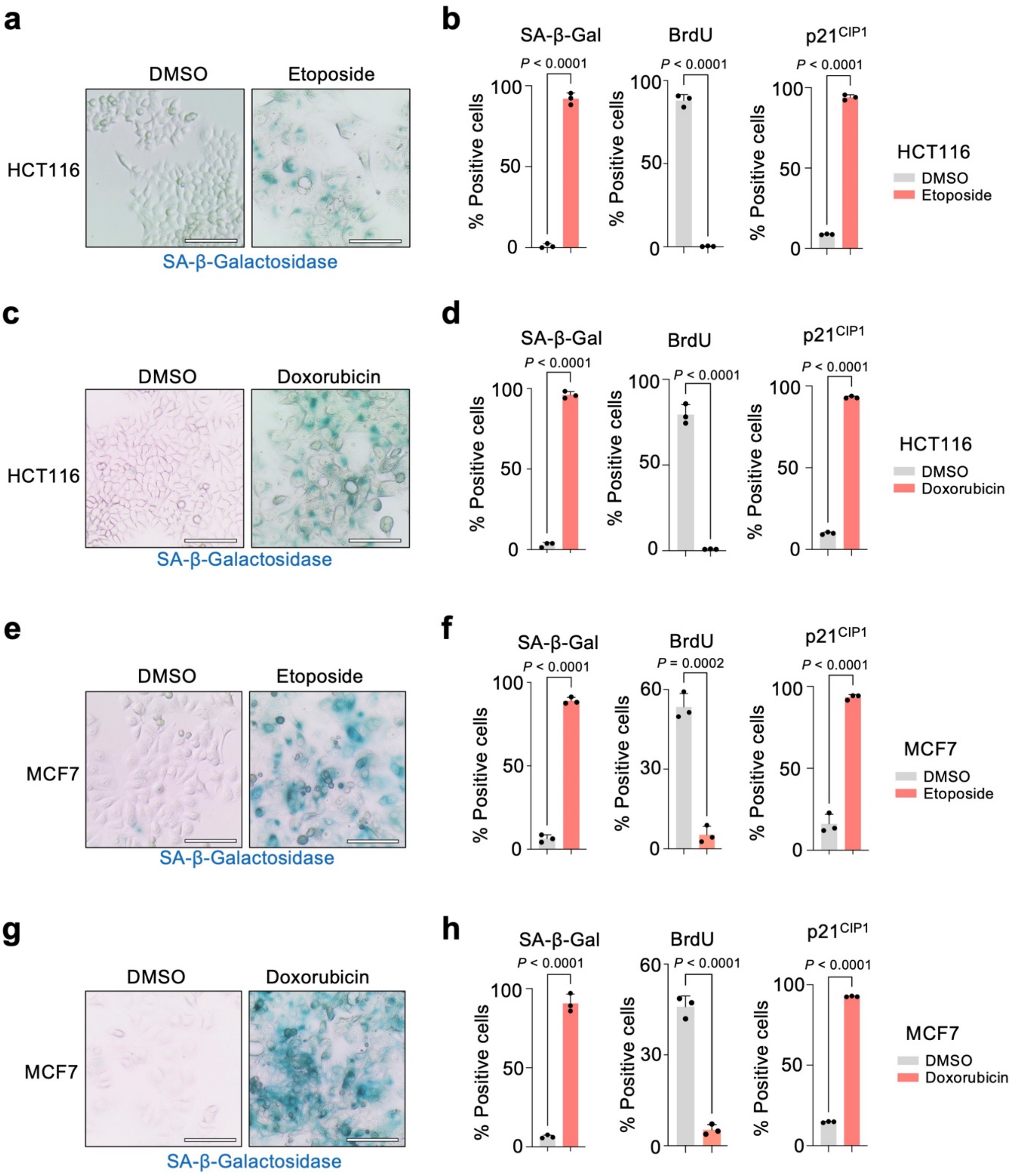

Supplemental Figure 15. Induction of senescence in HCT116 and MCF7 cancer cells. a, c, Representative images of three independent experiments of SA-β-gal staining in HCT116 cells 6 days after treatment with DMSO (control, a and c), etoposide (a), or doxorubicin (c). Scale bar, 100μm. **b, d,** Quantification of percentage positive control (DMSO) or therapy-induced senescent (etoposide, b or doxorubicin, d) HCT116 cells for SA-β-gal staining (left), BrdU incorporation (middle) or $p21^{CIP1}$ staining (right) on day 6. (n=3). Unpaired, two-tailed Student's t-test. Data represented as mean±SD. **e, g,** Representative images of three independent experiments of SA-

β-gal staining in MCF7 cells 6 days after treatment with DMSO (control, e, and g), etoposide (e), or doxorubicin (g). Scale bar, 100μm. **f, h,** Quantification of percentage positive control (DMSO) or therapy-induced senescent (etoposide, f or doxorubicin, h) MCF7 cells for SA-β-gal staining (left), BrdU incorporation (middle) or $p21^{CIP1}$ staining (right) on day 6. (n=3). Unpaired, two-tailed Student's t-test. Data represented as mean ± SD.

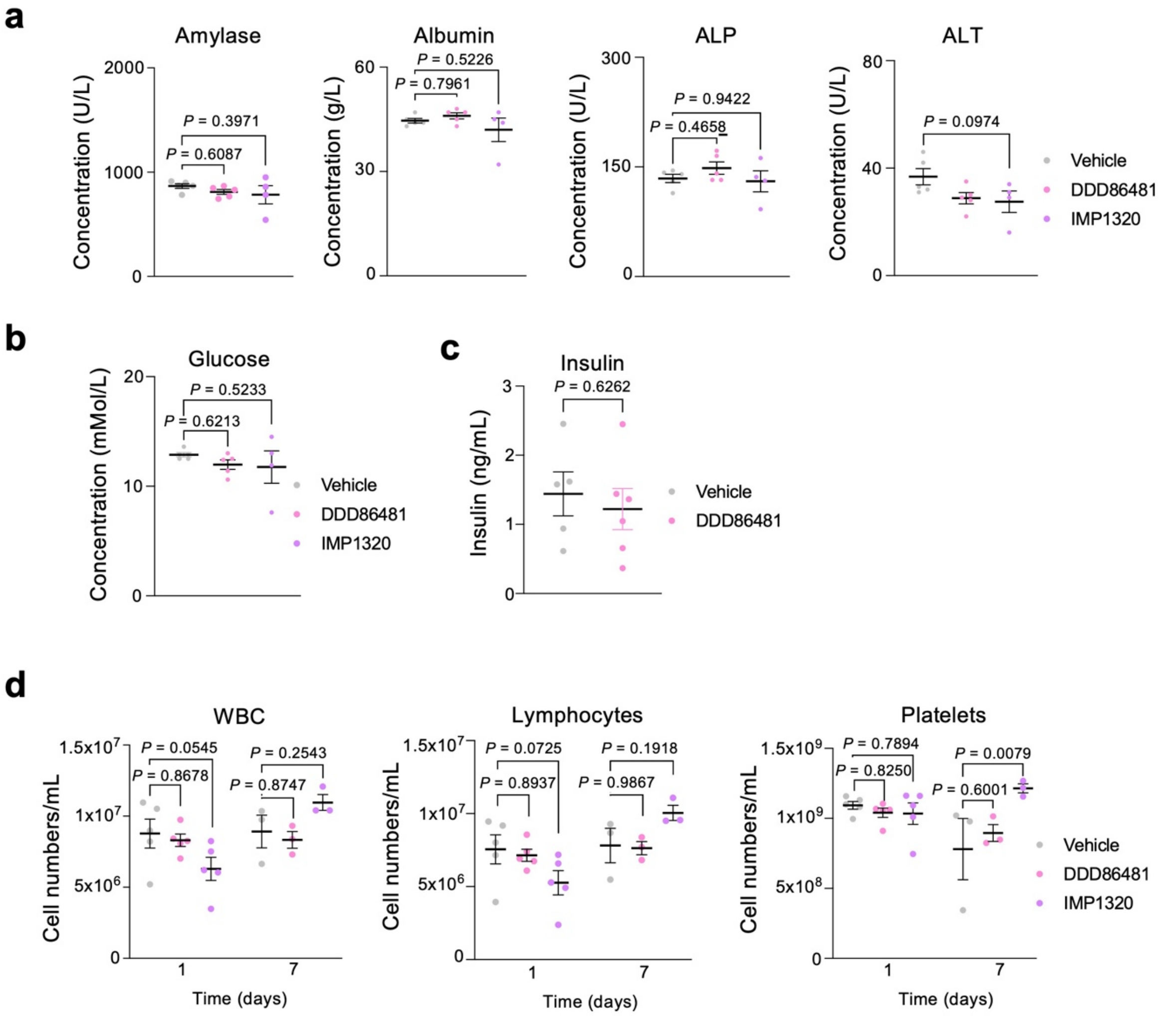

Supplemental Figure 16. NMTi are well tolerated *in vivo*. a Analysis of markers of liver function for C57/BL6 mice given 1 round of treatment with 10mg/kg DDD86481 or 25 mg/kg IMP1320. Blood was collected 7 days after. (n=5 mice for Vehicle and DDD86481, n=4 mice for IMP1320). One-way ANOVA, Dunnett's correction. **b,** Blood glucose levels for C57/BL6 mice given 1 round of treatment with 10mg/kg DDD86481 or 25 mg/kg IMP1320 and blood collected 7 days after. (n=5 mice for Vehicle and DDD86481, n=4 mice for IMP1320). One-way ANOVA. **c,** Blood insulin levels for C57/BL6 mice given 1 round of treatment with 10mg/kg DDD86481 and blood collected 7 days after. (n=5 mice for Vehicle, n=6 mice for DDD86481) Unpaired, two-tailed, Student's t-test. **d,** Blood cell counts for C57/BL6 mice given 1 round of treatment with 10mg/kg DDD86481 or 25 mg/kg IMP1320 and blood collected 1 and 7 days after. (day 1, n=5 mice per group; day 7, n=3 per group). Two-way ANOVA. Data represented as mean ± SD. N represents number of mice throughout the figure.

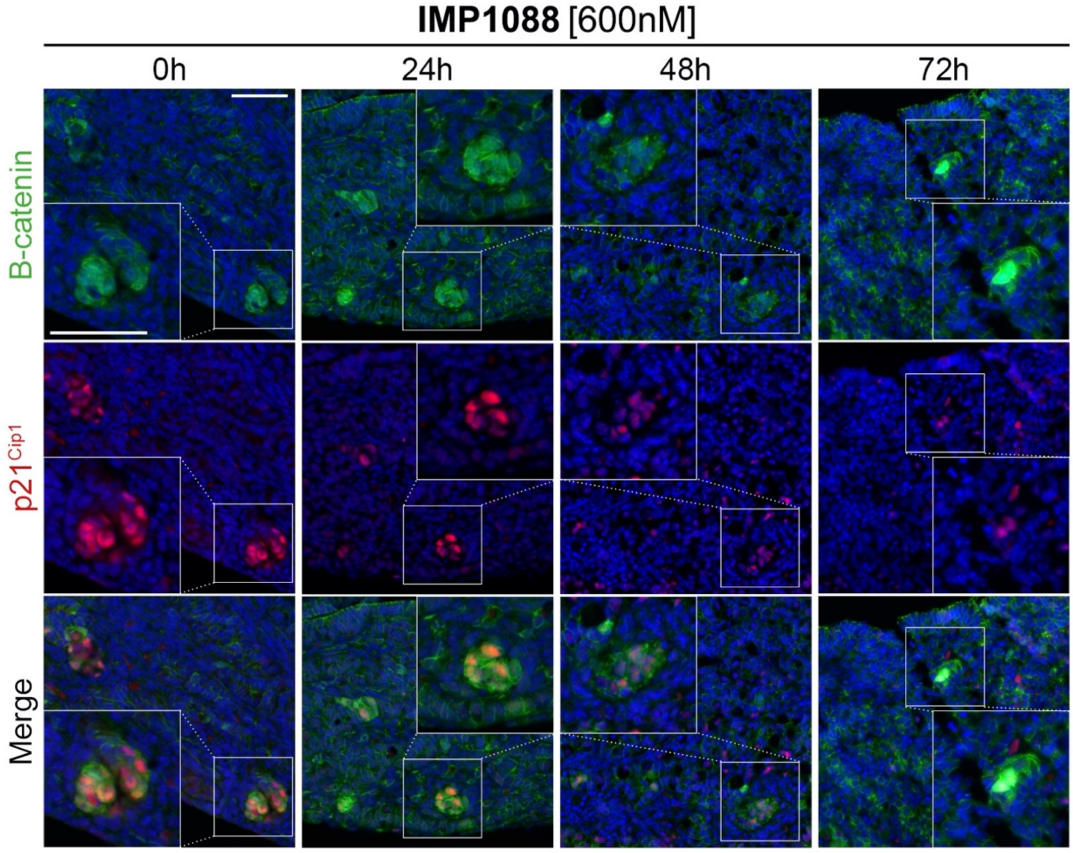

Supplemental Figure 17. Treatment with NMT inhibitors kills β-catenin-positive senescent clusters in a mouse model of adamantinomatous craniopharyngioma (ACP). Representative IF images of β-catenin (green) and p21^{Cip1} (red). Quantification and numbers of sections stained are available in Extended Data Figure 7f-g. Main scale bar, 50μm. Insert scale bar, 40μm.

Source gel images for Supplemental Figure 2a

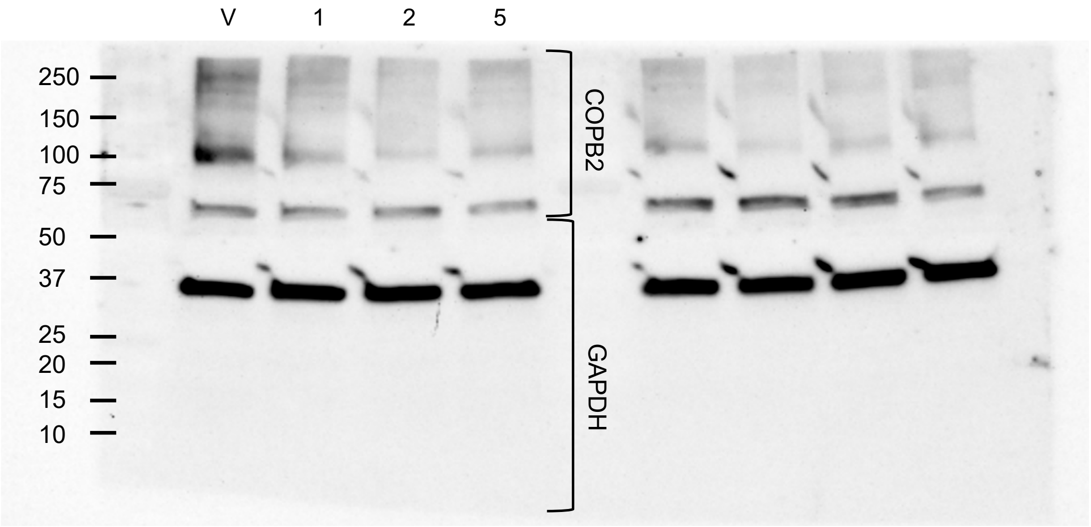

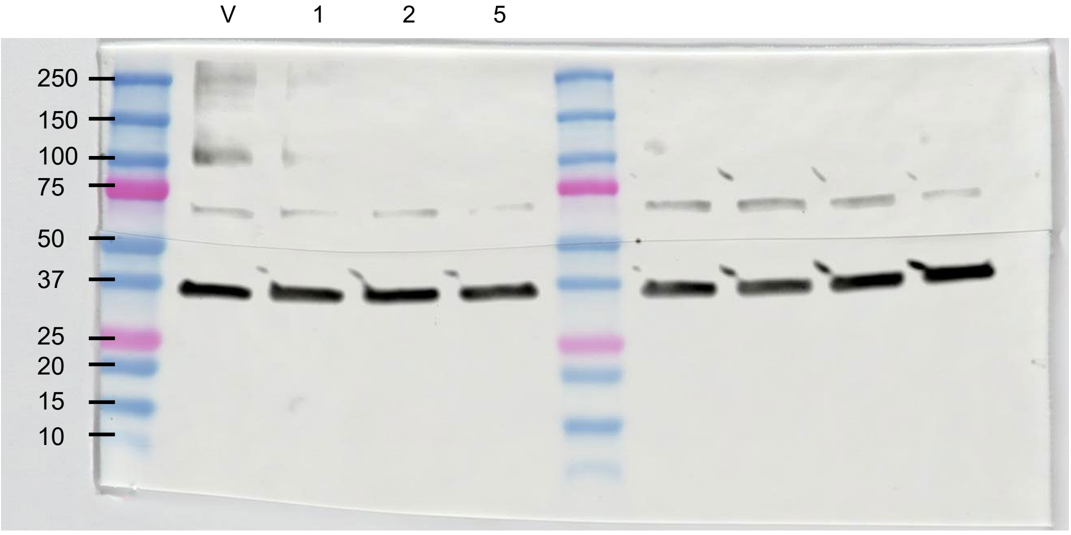

Uncropped images of western blots shown in Supplemental Figure 2a. The molecular weights (kDa) of size markers are indicated.

Source gel images for Supplemental Figure 14

Sup. Fig 14 a

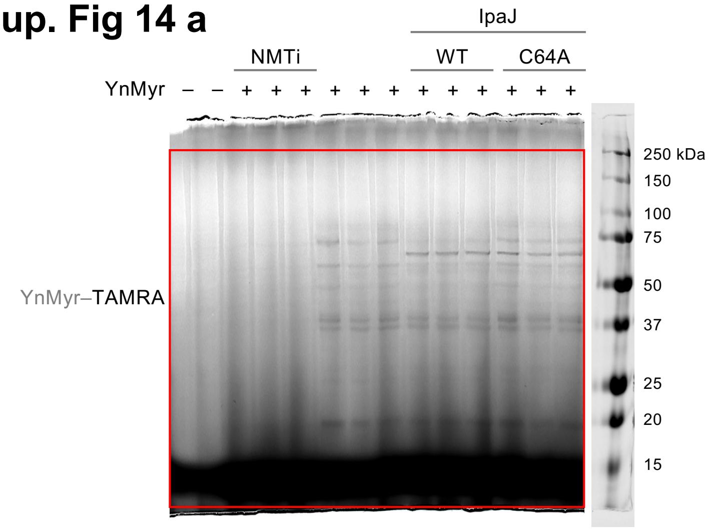

Sup. Fig 14 e

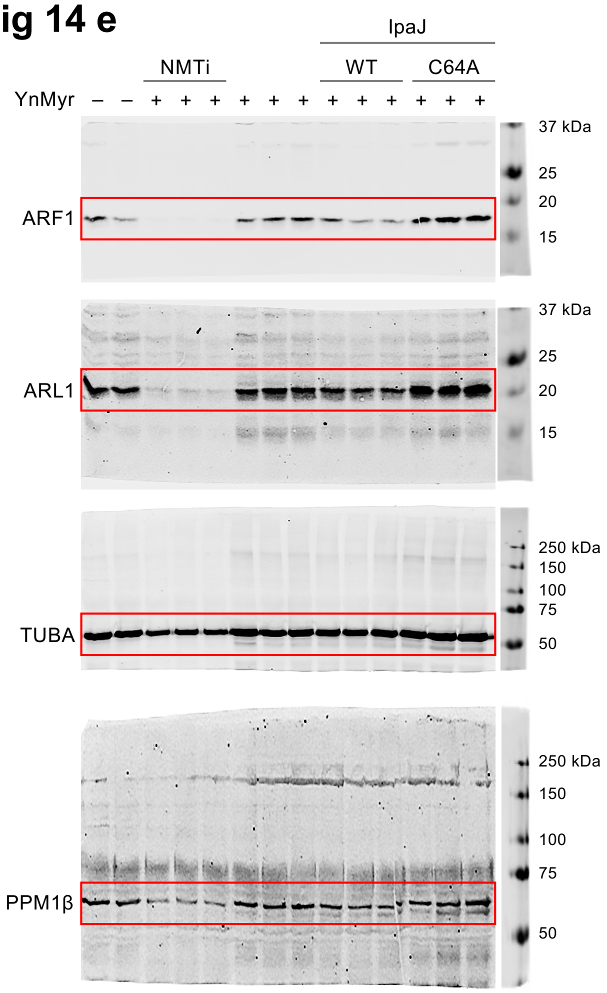

Uncropped images of gels and western blots shown in Supplemental Figure 14. The molecular weights (kDa) of size markers are indicated. Red boxes mark the cropped images shown in the Figure.

Source Data for Supplementary Figure 1

Supplementary Figure 1a

Dharmafect Conc. (uL/well)	DMSO.1	DMSO.2	DMSO.3	4OHT.1	4OHT.2	4OHT.3	Adjusted P Value	
0	0.631	0.122	0.124	6.884	1.332	3.226	0.338103	Unpaired t test
0.05	23.499	21.006	19.697	40.315	24.512	35.44	0.291391	
0.1	47.464	49.754	58.933	50	46.301	49.685	0.653636	
0.2	77.234	80.518	80.482	74.52	80.12	74.155	0.54118	
0.4	81.656	84.17	82.79	87.077	87.158	77.937	0.725427	

Supplementary Figure 1b

	DMSO.1	DMSO.2	4OHT.1	4OHT.2
siNT	1.110781	1.094302	1.093728	1.124638
siGFP.1	0.493993	0.504118	0.417788	0.391483
siGFP.2	0.566085	0.776096	0.464326	0.644206

Supplementary Figure 1c

	DMSO.1	DMSO.2	DMSO.3	4OHT.1	4OHT.2	4OHT.3	Adjusted P value	
siBCL2L1.2	99.06614	100.386	118.4433	67.74496	62.88424	77.90324	0.043735	Unpaired t test
siBCL2L1.3	101.7135	101.5837	119.8564	80.46487	57.61936	72.1212	0.054807	
siBCL2L1.4	98.49212	103.5138	122.1337	62.36417	46.20667	56.45207	0.020888	
siBCL2L1 Pool	99.78975	104.4536	102.9255	54.03859	42.51618	53.38653	0.001537	
siTOX	10.89145	5.065156	4.986716	3.08618	1.294498	2.564366	0.158555	
ABT	86.79257	93.41707	94.80006	55.61398	45.63638	48.63794	0.002887	
siBCL2L1 + BCL2L2 Pool	95.38432	90.40302	79.30462	42.1875	62.86849	64.57789	0.061744	
siBCL2L2 Pool	104.8683	101.8018	92.46947	82.24432	97.0425	98.71987	0.334605	

Source Data for Supplementary Figure 2

Supplementary Figure 2a

	Rep.1	Rep.2	Rep.3	One-way ANOVA Dunnet's
shEV	1	1	1	Adjusted P Value
shCOPB2.1	0.311399	0.327204	0.254162	<0.0001
shCOPB2.2	0.349245	0.272553	0.302413	<0.0001
shCOPB2.5	0.319932	0.282459	0.2927	<0.0001

Supplementary Figure 2b

	Rep.1	Rep.2	Rep.3	One-way ANOVA Dunnet's
shEV	1	1	1	Adjusted P Value
shCOPG1.1	0.254483	0.292631	0.23936	<0.0001
shCOPG1.4	0.285158	0.301638	0.269622	<0.0001
shCOPG1.5	0.277991	0.27348	0.271942	<0.0001

Supplementary Figure 2c

	DMSO.1	DMSO.2	DMSO.3	DMSO.4	DOXO.1	DOXO.2	DOXO.3	DOXO.4	Unpaired t test Adjusted P Value
shEV	91.20011	103.4575	88.47653	91.31822	88.72207	101.087	79.6937	96.12903	0.713901
shBCL2L1	108.0724	108.9506	97.26963	101.7471	28.91391	19.06117	20.89977	25.53192	0.000001
shCOPG1.1	80.90282	73.5155	76.53563	78.98922	8.484848	12.50766	15.62842	12.81302	<0.000001
shCOPG1.4	81.83934	65.29251	78.55731	75.30604	15.63118	14.28571	12.73101	15.95952	0.000006

Source Data for Supplementary Figure 3

Supplementary Figure 3a

	RAS DMSO + DMSO.1	RAS DMSO + DMSO.2	RAS DMSO + DMSO.3	RAS DMSO + DMSO.4	RAS DMSO + DMSO.5
ABT-263 1µM	0.845677	0.940242	1.030874	1.036787	0.888152
GCA 2.5µM	0.564715	0.518661	0.632552	0.751005	0.712037
BFA 150nM	0.611786	0.549786	0.662609	0.824307	0.753935

	RAS DMSO + QVD.1	RAS DMSO + QVD.2	RAS DMSO + QVD.3	RAS DMSO + QVD.4	RAS DMSO + QVD.5
ABT-263 1µM	1.198458	1.086715	1.021203	0.998022	1.027086
GCA 2.5µM	0.760786	0.923665	0.822998	0.827853	0.72499
BFA 150nM	0.838847	0.891444	0.797674	0.76227	0.76842

	RAS 4OHT + DMSO.1	RAS 4OHT + DMSO.2	RAS 4OHT + DMSO.3	RAS 4OHT + DMSO.4	RAS 4OHT + DMSO.5
ABT-263 1µM	0.508211	0.446366	0.543008	0.492936	0.54753
GCA 2.5µM	0.170854	0.136315	0.279871	0.239358	0.278656
BFA 150nM	0.267212	0.193596	0.407281	0.365688	0.328143

	RAS 4OHT+ QVD.1	RAS 4OHT+ QVD.2	RAS 4OHT+ QVD.3	RAS 4OHT+ QVD.4	RAS 4OHT+ QVD.5
ABT-263 1µM	1.007823	0.982785	0.941395	0.957904	1.02285
GCA 2.5µM	0.897892	0.817776	0.877209	0.853097	0.882245
BFA 150nM	0.604258	0.689365	0.800465	0.696665	0.73129

TW-ANOVA Dunnet's

Comparison (ABT)	Adjusted P Value
IMR90 ER:RAS + DMSO (4OHT) vs. IMR90 ER:RAS + DMSO (DMSO)	<0.0001
IMR90 ER:RAS + DMSO (4OHT) vs. IMR90 ER:RAS + QVD (DMSO)	<0.0001
IMR90 ER:RAS + DMSO (4OHT) vs. IMR90 ER:RAS + QVD (4OHT)	<0.0001

Comparison (GCA)	Adjusted P Value
IMR90 ER:RAS + DMSO (4OHT) vs. IMR90 ER:RAS + DMSO (DMSO)	<0.0001
IMR90 ER:RAS + DMSO (4OHT) vs. IMR90 ER:RAS + QVD (DMSO)	<0.0001
IMR90 ER:RAS + DMSO (4OHT) vs. IMR90 ER:RAS + QVD (4OHT)	<0.0001

Comparison (BFA)	
IMR90 ER:RAS + DMSO (4OHT) vs. IMR90 ER:RAS + DMSO (DMSO)	<0.0001
IMR90 ER:RAS + DMSO (4OHT) vs. IMR90 ER:RAS + QVD (DMSO)	<0.0001
IMR90 ER:RAS + DMSO (4OHT) vs. IMR90 ER:RAS + QVD (4OHT)	<0.0001

Supplementary Figure 3b

	DMSO+DMSO.1	DMSO+DMSO.2	DMSO+DMSO.3
GCA	0.866911	0.931705	0.976331
BFA	1.00328	0.921576	0.992193

	4OHT + DMSO.1	4OHT + DMSO.2	4OHT + DMSO.3
GCA	0.271314	0.338672	0.351694
BFA	0.242445	0.230496	0.217613

	4OHT + YVAD.1	4OHT + YVAD.2	4OHT + YVAD.3
GCA	0.288193	0.325851	0.387651
BFA	0.236519	0.385689	0.215893

	4OHT + VX-765.1	4OHT + VX-765.2	4OHT + VX-765.3
GCA	0.335451	0.288459	0.327277
BFA	0.267185	0.26973	0.226744

	4OHT +Necrostat	4OHT +Necrostatin.2	4OHT +Necrostatin.3
GCA	0.274074	0.237747	0.265096
BFA	0.246273	0.248334	0.197744

	4OHT +Liproxstat	4OHT +Liproxstatin.2	4OHT +Liproxstatin.3
GCA	0.300285	0.298382	0.299317
BFA	0.269772	0.253718	0.193328

TW-ANOVA Dunnet's

Comparison (GCA)	justed P Value
4OHT +DMSO vs. DMSO + DMSO	<0.0001
4OHT +DMSO vs. 4OHT + YVAD	0.9945
4OHT +DMSO vs. 4OHT + VX-765	0.9999
4OHT +DMSO vs. 4OHT + Necrosta	0.2995
4OHT +DMSO vs. 4OHT + Liproxsta	0.9598

Comparison (BFA)	justed P Value
4OHT +DMSO vs. DMSO + DMSO	<0.0001
4OHT +DMSO vs. 4OHT + YVAD	0.5023
4OHT +DMSO vs. 4OHT + VX-765	0.9318
4OHT +DMSO vs. 4OHT + Necrosta	0.9999
4OHT +DMSO vs. 4OHT + Liproxsta	0.9992

Supplementary Figure 3c

Time (hr)	DMSO + siNT.1	DMSO + siNT.2	DMSO + siCOPB2_4.1	DMSO + siCOPB2_4.2	DMSO + siCOPB2_3.1	DMSO + siCOPB2_3.2	4OHT + siNT.1	4OHT + siNT.2	4OHT +siCOPB2_4.1	4OHT +siCOPB2_4.2	4OHT +siCOPB2_3.1	4OHT +siCOPB2_3.2
0	0	0.00325	0	0	0	0	0.00325	0	0.0065	0.00325	0.00325	0.01
2	0	0.00325	0.0065	0	0	0.00325	0.02675	0.00975	0	0.00675	0.0065	0.01
4	0.00325	0.00675	0.01	0.00325	0	0.0065	0.0065	0.0165	0.0065	0.013	0.02325	0.0065
6	0	0.00675	0.00325	0	0.0065	0	0.00975	0.047	0	0.0165	0.0065	0.0235
8	0	0.00675	0	0	0	0.0065	0.0165	0.04025	0.01325	0.02675	0.02	0.0335
10	0	0.00675	0.0065	0	0	0.00675	0.00675	0.0335	0.00975	0.02325	0.0435	0.037
12	0.00325	0.00325	0	0.00325	0.00325	0.0065	0.01	0.04375	0.03	0.02	0.05725	0.01675
14	0	0.00975	0	0.00325	0.0065	0.00325	0.01325	0.05025	0.03	0.04375	0.04025	0.01675
16	0.00325	0.01	0	0.00325	0.00975	0.0065	0.01325	0.05025	0.04025	0.037	0.04025	0.02
18	0.00325	0.00325	0.0065	0.00975	0.01	0.0065	0.02675	0.047	0.03025	0.0435	0.057	0.02325
20	0.00325	0	0.00325	0.00675	0.00325	0.00325	0.01325	0.0335	0.03675	0.04675	0.0435	0.03025
22	0	0	0.00325	0.0065	0.00325	0.01	0.0165	0.0605	0.04375	0.0575	0.037	0.02675
24	0	0	0	0	0.00325	0.00325	0.0235	0.047	0.08425	0.0335	0.0535	0.07075
26	0	0	0.00325	0.00325	0.00325	0	0.01675	0.06725	0.09075	0.05375	0.06375	0.098
28	0	0	0.00675	0.00675	0.0065	0.0065	0.02	0.0775	0.176	0.0975	0.07075	0.176
30	0	0	0	0.00325	0.00975	0	0.03	0.064	0.264	0.11475	0.12175	0.264
32	0.00325	0.00325	0	0.00325	0.00675	0	0.04375	0.07075	0.35225	0.1555	0.16225	0.3895
34	0.00325	0.00325	0.0065	0.00975	0.00325	0.0065	0.057	0.098	0.53175	0.21325	0.25375	0.57275
36	0	0.00325	0.01	0.00325	0.00675	0.00325	0.0775	0.091	0.6775	0.274	0.352	0.837
38	0	0	0.00975	0.00675	0.01325	0.0165	0.09425	0.08075	0.93175	0.403	0.552	1.1285
40	0	0	0.01325	0.01	0.01325	0.01325	0.1115	0.10475	1.1455	0.542	0.698	1.23725
42	0	0	0.00675	0.00675	0.01325	0	0.11475	0.1115	1.35925	0.742	0.783	1.45425
44	0	0	0.01	0.00675	0.02025	0.00325	0.142	0.108	1.55925	1.0235	1.054	1.59975
46	0	0	0.00975	0.0065	0.01975	0.027	0.14175	0.16225	1.82375	1.23025	1.22375	1.81
48	0	0.00325	0.01	0	0.0165	0.01325	0.15225	0.1995	1.9495	1.366	1.332	1.87825
50	0	0.00325	0.01325	0.0135	0.01325	0.01	0.1555	0.19625	2.13225	1.4915	1.56275	2.0305
52	0	0.00675	0.02325	0.02	0.00675	0.01	0.16225	0.23375	2.3765	1.6575	1.6475	2.16975
54	0.00325	0.00325	0.0505	0.01325	0.02325	0.0235	0.14875	0.22025	2.47125	1.8375	1.7625	2.21025
56	0.0065	0.00325	0.07075	0.01	0.01325	0.0335	0.159	0.21675	2.641	1.96275	1.824	2.2715
58	0.00325	0	0.08425	0.01675	0.0135	0.037	0.18925	0.26725	2.8375	1.9665	1.95575	2.44075
60	0.00325	0	0.1115	0.02325	0.0165	0.0605	0.213	0.31175	2.95625	1.99675	2.05775	2.519
62	0	0.00325	0.159	0.037	0.02325	0.06075	0.21325	0.31825	2.99375	2.163	2.13225	2.62075
64	0.00325	0	0.24025	0.03025	0.04025	0.1045	0.20675	0.3215	3.075	2.25425	2.2815	2.651
66	0.0065	0	0.37575	0.03675	0.02675	0.1285	0.19625	0.35225	3.24775	2.3155	2.22725	2.597
68	0	0.00325	0.52175	0.047	0.047	0.1995	0.19625	0.33175	3.22425	2.33575	2.2575	2.64425
70	0.00325	0.00325	0.637	0.064	0.047	0.264	0.20625	0.376	3.23425	2.29525	2.2815	2.519
72	0	0	0	0	0	0	0	0	0	0	0	0

Supplementary Figure 3d

	DMSO.1	DMSO.2	DMSO.3	4OHT.1	4OHT.2	4OHT.3	4OHT.4
siNT	0.275	0.093	0.592	1.738	1.01	1.845	2.683
siBCL2L1	0.867	1.109	3.771	13.765	5.021	7.774	5.392
siCOPB2.1	0.591	0.29	0.917	5.205	5.984	3.8	10.073
siCOPB2.2	0.602	0.491	1.375	7.663	8.875	4.005	11.4
siCOPB2.3	0.823	0.271	1.006	8.314	8.657	4.686	9.329
siCOPB2.4	0.607	0.426	0.802	4.661	5.219	5.028	4.841

Comparison (DMSO)	Adjusted P Value
siNT vs. siBCL2L1	0.789
siNT vs. siCOPB2.1	0.9999
siNT vs. siCOPB2.2	0.998
siNT vs. siCOPB2.3	0.9995
siNT vs. siCOPB2.4	0.9999

Comparison (4OHT)	Adjusted P Value
siNT vs. siBCL2L1	0.0006
siNT vs. siCOPB2.1	0.0159
siNT vs. siCOPB2.2	0.0007
siNT vs. siCOPB2.3	0.001
siNT vs. siCOPB2.4	0.1302

Source Data for Supplementary Figure 4

Supplementary Figure 4a

	DMSO.1	DMSO.2	DMSO.3	BLEO.1	BLEO.2	BLEO.3	IRRD.1	IRRD.2	IRRD.3
SA-bgal %	1.342282	2.985075	4	88.88889	89.65517	95.2381	82.6087	94.91525	89.39394
	DMSO.1	DMSO.2	DMSO.3	BLEO.1	BLEO.2	BLEO.3	IRRD.1	IRRD.2	IRRD.3
BrdU %	71.788	73.768	73.566	1.667	0.123	0.586	1.587	1.287	1.205

Ordinary OW-ANOVA

Dunnett's multiple comparisons test (SA-bgal)	Adjusted P Value
DMSO vs. Bleomycin	<0.0001
DMSO vs. 20Gy	<0.0001

Dunnett's multiple comparisons test (BrdU)	Adjusted P Value
DMSO vs. Bleomycin	<0.0001
DMSO vs. 20Gy	<0.0001

Supplementary Figure 4b

	DMSO.1	DMSO.2	DMSO.3	BLEO.1	BLEO.2	BLEO.3	P value	Unpaired t test
SA-bgal %	20	30	16.2	90	83	77.2	0.0004	
	DMSO.1	DMSO.2	DMSO.3	BLEO.1	BLEO.2	BLEO.3	P value	Unpaired t test
BrdU %	73	72	54.4378	0.1	0.1	2.434844	0.0004	

Supplementary Figure 4c

DATA NHLF BFA

Conc (uM)	DMSO.1	DMSO.2	DMSO.3	BLEO.1	BLEO.2	BLEO.3	Adjusted P Value	Unpaired t test
0.04	101.448	88.51988	95.27752	75.6689	70.48301	60.9607	0.020426	
0.15	84.00285	87.30281	94.12085	40.21655	48.65865	55.72052	0.006799	
1.25	64.1097	67.18421	72.54609	28.14699	55.78551	38.08612	0.032049	
10	57.92342	70.78242	71.01465	19.64048	37.01172	26.67877	0.012816	

DATA NHLF GCA

Drug/Conc (uM)	DMSO.1	DMSO.2	DMSO.3	BLEO.1	BLEO.2	BLEO.3	Adjusted P Value	Unpaired t test
ABT	95.48299	101.9354	92.04556	53.23166	58.95652	57.3784	0.00115	
1.25	95.32229	87.15817	83.69392	98.20172	96.33114	94.06699	0.109829	
5	94.47522	71.81509	87.61367	42.32425	64.97512	54.92424	0.061267	
10	77.51737	82.69025	79.6228	47.47004	59.96094	49.36479	0.007976	

Supplementary Figure 4d

	DMSO.1	DMSO.2	DMSO.3	BLEO.1	BLEO.2	BLEO.3			P value	
SA-bgal %	15	17	20	93	81	91			<0.0001	Unpaired t test
	DMSO.1	DMSO.2	DMSO.3	DMSO.4	BLEO.1	BLEO.2	BLEO.3	BLEO.4	P value	
BrdU %	89.53667	87.74267	86.84533	85.75867	14.66867	5.794333	25.5078	25.3968	<0.0001	Unpaired t test

Supplementary Figure 4e

DATA PBEC BFA

Conc (uM)	DMSO.1	DMSO.2	DMSO.3	BLEO.1	BLEO.2	BLEO.3	Adjusted P Value	Unpaired t test
0.73	72.29478	75.6548	75.50767	36.28763	39.4033	37.3632	0.000027	
20	76.21269	75.78073	78.74824	44.18896	39.85157	44.44548	0.000041	

DATA PBEC GCA

Drug/Conc	DMSO.1	DMSO.2	DMSO.3	BLEO.1	BLEO.2	BLEO.3	Adjusted P Value	Unpaired t test
6.6	94.98135	87.41752	83.58386	32.21934	25.76084	30.50672	0.000106	
20	85.30311	88.79263	85.84969	26.91434	24.26071	25.23268	0.000003	

Supplementary Figure 4f

	p16_low.1	p16_low.2	p16_low.3	p16_high.1	p16_high.2	p16_high.3	Unpaired t test Adjusted P Value
9nM	113.3454	116.3967	105.9306	83.63321	78.21012	83.42396	0.001088
80nM	131.8425	155.34	134.3634	52.85064	33.85214	48.74942	0.001073
0.73μM	84.77139	98.13229	88.3791	2.211142	2.918288	1.6778	0.000101
20μM	100.3622	111.4127	94.17307	4.335573	3.418566	4.334317	0.000125

Source Data for Supplementary Figure 5

Supplementary Figure 5a

	DMSO +DMSO.1	DMSO +DMSO.2	DMSO +DMSO.3	4OHT + DMSO.1	4OHT + DMSO.2	4OHT + DMSO.3
% BrdU	36.337	44.731	29.891	6.475	16.295	16.683

	4OHT + BEC.1	4OHT + BEC.2	4OHT + BEC.3	4OHT + TRI.1	4OHT + TRI.2	4OHT + TRI.3
% BrdU	7.825	18.378	24.584	4.427	5.748	18.907

Dunnett's multiple comparisons test	Adjusted P Value
RAS 4OHT vs. RAS DMSO	0.0116
RAS 4OHT vs. RAS 4OHT + BEC	0.87
RAS 4OHT vs. RAS 4OHT + TRI	0.8951

Supplementary Figure 5c

	DMSO +DMSO.1	DMSO +DMSO.2	DMSO +DMSO.3	4OHT + DMSO.1	4OHT + DMSO.2	4OHT + DMSO.3
% p16	9.027	8.698	22.809	69.973	71.208	70.311
	4OHT + BEC.1	4OHT + BEC.2	4OHT + BEC.3	4OHT + TRI.1	4OHT + TRI.2	4OHT + TRI.3
% p16	82.131	82.609	71.595	86.993	86.237	71.146

Dunnett's multiple comparisons test (p16)	Adjusted P Value
RAS 4OHT vs. RAS DMSO	<0.0001
RAS 4OHT vs. RAS 4OHT + BEC	0.3648
RAS 4OHT vs. RAS 4OHT + TRI	0.1882

% p21	DMSO +DMSO.1	DMSO +DMSO.2	DMSO +DMSO.3	4OHT + DMSO.1	4OHT + DMSO.2	4OHT + DMSO.3
	21.654	23.034	22.342	81.923	77.25	71.858
	4OHT + BEC.1	4OHT + BEC.2	4OHT + BEC.3	4OHT + TRI.1	4OHT + TRI.2	4OHT + TRI.3
% p21	72.63	70.606	65.684	66.756	76.123	56.135

Dunnett's multiple comparisons test (p21)	Adjusted P Value
RAS 4OHT vs. RAS DMSO	<0.0001
RAS 4OHT vs. RAS 4OHT + BEC	0.3475
RAS 4OHT vs. RAS 4OHT + TRI	0.1328

Supplementary Figure 5d

	DMSO +DMSO.1	DMSO +DMSO.2	DMSO +DMSO.3	4OHT + DMSO.1	4OHT + DMSO.2	4OHT + DMSO.3
IL6 %	0.896	1.022	0.727	36.943	31.026	36.155

	4OHT + BEC.1	4OHT + BEC.2	4OHT + BEC.3	4OHT + TRI.1	4OHT + TRI.2	4OHT + TRI.3
IL6 %	13.439	12.496	4.372	13.949	8.429	8.455

Dunnett's multiple comparisons test (IL6)	Adjusted P Value
RAS 4OHT vs. RAS DMSO	<0.0001
RAS 4OHT vs. RAS 4OHT + BEC	<0.0001
RAS 4OHT vs. RAS 4OHT + TRI	<0.0001

	DMSO +DMSO.1	DMSO +DMSO.2	DMSO +DMSO.3	4OHT + DMSO.1	4OHT + DMSO.2	4OHT + DMSO.3
IL8 %	0.404	0.482	1.383	42.084	48.411	50.209

	4OHT + BEC.1	4OHT + BEC.2	4OHT + BEC.3	4OHT + TRI.1	4OHT + TRI.2	4OHT + TRI.3
	3.227	3.313	16.825	3.415	2.207	18.536

Dunnett's multiple comparisons test (IL8)	Adjusted P Value
RAS 4OHT vs. RAS DMSO	<0.0001
RAS 4OHT vs. RAS 4OHT + BEC	0.0002
RAS 4OHT vs. RAS 4OHT + TRI	0.0002

Supplementary Figure 5e

	Rep.1	Rep.2
Vector	1	1
53	0.15745	0.20758
86	0.107768	0.117013

Supplementary Figure 5f

	DMSO + Vector.1	DMSO + Vector.2	DMSO + Vector.3	DMSO + Vector.4	4OHT + Vector.1	4OHT + Vector.2	4OHT + Vector.3	4OHT + Vector.4
% BrdU	40.366	35.237	34.309	43.374	7.9835	4.9785	6.45	7.054

	4OHT + shPTBP1_53.1	4OHT + shPTBP1_53.2	4OHT + shPTBP1_53.3	4OHT + shPTBP1_53.4	4OHT + shPTBP1_86.1	4OHT + shPTBP1_86.2	4OHT + shPTBP1_86.3	4OHT + shPTBP1_86.4
% BrdU	10.0605	5.887	2.0435	12.9835	5.2335	3.8555	2.0435	5.0415

Dunnett's multiple comparisons test (BrdU)	Adjusted P Value
4OHT vs. DMSO	<0.0001
4OHT vs. 53	0.9332
4OHT vs. 86	0.5837

	DMSO + Vector.1	DMSO + Vector.2	DMSO + Vector.3	DMSO + Vector.4	4OHT + Vector.1	4OHT + Vector.2	4OHT + Vector.3	4OHT + Vector.4
% p16	2.8815	20.759	17.2105	19.5475	79.776	75.8665	77.547	72.597

	4OHT + shPTBP1_53.1	4OHT + shPTBP1_53.2	4OHT + shPTBP1_53.3	4OHT + shPTBP1_53.4	4OHT + shPTBP1_86.1	4OHT + shPTBP1_86.2	4OHT + shPTBP1_86.3	4OHT + shPTBP1_86.4
% p16	88.309	88.401	80.5925	74.0625	85.188	86.1655	83.3735	78.258

Dunnett's multiple comparisons test (p16)	Adjusted P Value
4OHT vs. DMSO	<0.0001
4OHT vs. 53	0.3258
4OHT vs. 86	0.2828

	DMSO + Vector.1	DMSO + Vector.2	DMSO + Vector.3	DMSO + Vector.4	4OHT + Vector.1	4OHT + Vector.2	4OHT + Vector.3	4OHT + Vector.4
% p21	26.4455	16.2165	26.264	30.639	56.4245	59.6695	54.748	60.0985

	4OHT + shPTBP1_53.1	4OHT + shPTBP1_53.2	4OHT + shPTBP1_53.3	4OHT + shPTBP1_53.4	4OHT + shPTBP1_86.1	4OHT + shPTBP1_86.2	4OHT + shPTBP1_86.3	4OHT + shPTBP1_86.4
% p21	42.9235	56.5105	41.292	56.435	45.8195	53.7735	48.913	61.3855

Dunnett's multiple comparisons test (p21)	Adjusted P Value
4OHT vs. DMSO	<0.0001
4OHT vs. 53	0.1918
4OHT vs. 86	0.5237

Supplementary Figure 5h

	DMSO + Vector .1	DMSO + Vector .2	DMSO + Vector .3	DMSO + Vector .4	DMSO + shPTBP1_53.1	DMSO + shPTBP1_53.2	DMSO + shPTBP1_53.3	DMSO + shPTBP1_53.4	DMSO + shPTBP1_86.1	DMSO + shPTBP1_86.2	DMSO + shPTBP1_86.3	DMSO + shPTBP1_86.4
siNT	100.307	103.538	100.324	98.453	93.293	102.352	92.605	97.127	98.319	101.805	97.643	94.916
siBCL2L1	52.622	45.341	63.965	45.841	67.214	60.013	53.6	56.016	62.144	45.569	48.527	46.697
siCOPB2.1	94.033	96.861	92.12	92.553	89.712	93.975	87.453	85.768	91.429	95.457	89.598	92.082
siCOPB2.2	86.727	94.702	87.394	90.677	84.192	91.574	85.162	84.257	90.495	88.096	86.011	80.433
siCOPB2.3	90.235	90.633	84.065	80.464	84.873	94.442	81.017	82.447	86.992	91.036	81.194	75.787
siCOPB2.4	91.826	96.911	89.19	89.942	87.266	102.836	85.666	82.522	97.828	102.474	90.879	75.953

	4OHT + Vector .1	4OHT + Vector .2	4OHT + Vector .3	4OHT + Vector .4	4OHT + shPTBP1_53.1	4OHT + shPTBP1_53.2	4OHT + shPTBP1_53.3	4OHT + shPTBP1_53.4	4OHT + shPTBP1_86.1	4OHT + shPTBP1_86.2	4OHT + shPTBP1_86.3	4OHT + shPTBP1_86.4
siNT	110.204	92.78	94.768	97.896	92.816	103.77	79.683	87.761	94.574	101.827	100	91.998
siBCL2L1	19.754	22.942	22.879	29.994	25.975	27.91	20.616	33.688	19.668	14.12	14.374	21.797
siCOPB2.1	44.705	55.43	48.529	56.818	76.023	89.153	74.679	79.042	62.622	79.568	72.329	66.667
siCOPB2.2	46.746	43.735	46.521	43.462	61.323	55.556	63.858	64.061	72.796	52.326	58.955	53.608
siCOPB2.3	42.742	45.346	50.99	43.603	58.183	60.714	65.355	66.68	50.867	61.794	56.418	61.267
siCOPB2.4	49.288	52.506	45.786	47.278	65.985	90.41	63.644	85.796	77.995	94.767	65.949	66.765

TW-ANOVA Dunnet's

Dunnett's multiple comparisons test (siNT)	Adjusted P Value
RAS 4OHT (shEV) vs. RAS DMSO (shEV)	0.995
RAS 4OHT (shEV) vs. RAS DMSO (shPTBP1_53)	0.9725
RAS 4OHT (shEV) vs. RAS DMSO (shPTBP1_86)	>0.9999
RAS 4OHT (shEV) vs. RAS 4OHT (shPTBP1_53)	0.2955
RAS 4OHT (shEV) vs. RAS 4OHT (shPTBP1_86)	0.994

Dunnett's multiple comparisons test (siBCL2L1)	
RAS 4OHT (shEV) vs. RAS DMSO (shEV)	<0.0001
RAS 4OHT (shEV) vs. RAS DMSO (shPTBP1_53)	<0.0001
RAS 4OHT (shEV) vs. RAS DMSO (shPTBP1_86)	<0.0001
RAS 4OHT (shEV) vs. RAS 4OHT (shPTBP1_53)	0.937
RAS 4OHT (shEV) vs. RAS 4OHT (shPTBP1_86)	0.4948

Dunnett's multiple comparisons test (siCOPB2.1)	
RAS 4OHT (shEV) vs. RAS DMSO (shEV)	<0.0001
RAS 4OHT (shEV) vs. RAS DMSO (shPTBP1_53)	<0.0001
RAS 4OHT (shEV) vs. RAS DMSO (shPTBP1_86)	<0.0001
RAS 4OHT (shEV) vs. RAS 4OHT (shPTBP1_53)	<0.0001
RAS 4OHT (shEV) vs. RAS 4OHT (shPTBP1_86)	0.0004

Dunnett's multiple comparisons test (siCOPB2.2)	
RAS 4OHT (shEV) vs. RAS DMSO (shEV)	<0.0001
RAS 4OHT (shEV) vs. RAS DMSO (shPTBP1_53)	<0.0001
RAS 4OHT (shEV) vs. RAS DMSO (shPTBP1_86)	<0.0001
RAS 4OHT (shEV) vs. RAS 4OHT (shPTBP1_53)	0.0031
RAS 4OHT (shEV) vs. RAS 4OHT (shPTBP1_86)	0.0104

Dunnett's multiple comparisons test (siCOPB2.3)	
RAS 4OHT (shEV) vs. RAS DMSO (shEV)	<0.0001
RAS 4OHT (shEV) vs. RAS DMSO (shPTBP1_53)	<0.0001
RAS 4OHT (shEV) vs. RAS DMSO (shPTBP1_86)	<0.0001
RAS 4OHT (shEV) vs. RAS 4OHT (shPTBP1_53)	0.0015
RAS 4OHT (shEV) vs. RAS 4OHT (shPTBP1_86)	0.0445

Dunnett's multiple comparisons test (siCOPB2.4)	
RAS 4OHT (shEV) vs. RAS DMSO (shEV)	<0.0001
RAS 4OHT (shEV) vs. RAS DMSO (shPTBP1_53)	<0.0001
RAS 4OHT (shEV) vs. RAS DMSO (shPTBP1_86)	<0.0001
RAS 4OHT (shEV) vs. RAS 4OHT (shPTBP1_53)	<0.0001
RAS 4OHT (shEV) vs. RAS 4OHT (shPTBP1_86)	<0.0001

Source Data for Supplementary Figure 7

Supplementary Figure 7b

Immunofluorescence (24h)

	DMSO.1	DMSO.2	DMSO.3	4OHT.1	4OHT.2	4OHT.3	4OHT + GCA.1	4OHT + GCA.2	4OHT + GCA.3	4OHT + BFA.1	4OHT + BFA.2	4OHT + BFA.3
GM-CSF	0.111	0.11	0.111	0.128	0.125	0.125	0.146	0.144	0.147	0.138	0.139	0.141
CXCL1	0.116	0.121	0.129	0.155	0.158	0.153	0.223	0.214	0.213	0.211	0.187	0.186
BMP2/4	0.128	0.134	0.131	0.154	0.152	0.157	0.192	0.19	0.187	0.179	0.177	0.176

Dunnett's multiple comparisons test (24H)	Adjusted P Value
GM-CSF	
4OHT vs. DMSO	0.0032
4OHT vs. 4OHT+GCA	0.0003
4OHT vs. 4OHT+BFA	0.0104
CXCL1	Adjusted P Value
4OHT vs. DMSO	<0.0001
4OHT vs. 4OHT+GCA	<0.0001
4OHT vs. 4OHT+BFA	<0.0001
BMP2/4	Adjusted P Value
4OHT vs. DMSO	<0.0001
4OHT vs. 4OHT+GCA	<0.0001
4OHT vs. 4OHT+BFA	<0.0001

Immunofluorescence (48h)

	DMSO.1	DMSO.2	DMSO.3	4OHT.1	4OHT.2	4OHT.3	4OHT + GCA.1	4OHT + GCA.2	4OHT + GCA.3	4OHT + BFA.1	4OHT + BFA.2	4OHT + BFA.3
VEGF	0.259	0.254	0.261	0.316	0.33	0.342	0.506	0.518	0.558	0.442	0.464	0.485
GM-CSF	0.108	0.108	0.108	0.117	0.12	0.125	0.158	0.147	0.16	0.141	0.138	0.165
CXCL1	0.129	0.125	0.112	0.157	0.139	0.126	0.35	0.271	0.177	0.331	0.288	0.178
BMP2/4	0.137	0.136	0.137	0.155	0.153	0.179	0.25	0.246	0.211	0.229	0.214	0.211

Dunnett's multiple comparisons test (48H)	Adjusted P Value
VEGF	
4OHT vs. DMSO	0.0277
4OHT vs. 4OHT+GCA	<0.0001
4OHT vs. 4OHT+BFA	<0.0001
GM-CSF	
4OHT vs. DMSO	0.9309
4OHT vs. 4OHT+GCA	0.4269
4OHT vs. 4OHT+BFA	0.6003
CXCL1	
4OHT vs. DMSO	0.8179
4OHT vs. 4OHT+GCA	0.0001
4OHT vs. 4OHT+BFA	0.0001
BMP2/4	
4OHT vs. DMSO	0.6438
4OHT vs. 4OHT+GCA	0.0231
4OHT vs. 4OHT+BFA	0.1036

Supplementary Figure 7c

	DMSO.1	DMSO.2	DMSO.3	4OHT.1	4OHT.2	4OHT.3	4OHT+GCA.1	4OHT+GCA.2	4OHT+GCA.3	4OHT+BFA.1	4OHT+BFA.2	4OHT+BFA.3
IL-6	0.016845	0.034483	0.011984	1	1	1	0.06742619	0.00256479	0.05090407	0.01202528	0.00646676	0.0102069
IL-8	0.001173	0.000816	0.000536	1	1	1	0.11565341	0.120535	0.10948839	0.0433674	0.0496508	0.0426916
VEGF	0.279391	0.199205	0.219511	1	1	1	0.15645325	0.11412064	0.14186125	0.14952816	0.13866773	0.1043014
CXCL1	0.012896	0.010609	0.006122	1	1	1	0.0310824	0.04436487	0.03738563	0.01534418	0.0199838	0.0179759
IL-1β	0.000216	0.00017	0.000171	1	1	1	0.09054321	0.08551571	0.10781265	0.03430371	0.03614923	0.042212
CCL2	0.181719	0.694777	0.40005	1	1	1	0.01162324	0.01093481	0.01666009	0.00765936	0.005808	0.0055796
CCL20	9.18E-05	0.000305	0.000178	1	1	1	0.33637578	0.20720336	0.22846443	0.10135051	0.06868326	0.0423005
GM-CSF	9.87E-05	0.000128	7.93E-05	1	1	1	0.02843385	0.03404474	0.04195923	0.01605288	0.02206691	0.0218684
G-CSF	0.000147	9.92E-05	0.000174	1	1	1	0.07984031	0.05092391	0.05964541	0.01362553	0.01441372	0.0097202

Dunnett's multiple comparisons test	Adjusted P Value
IL-6	
4OHT vs. DMSO	<0.0001
4OHT vs. 4OHT+GCA	<0.0001
4OHT vs. 4OHT+BFA	<0.0001
IL-8	
4OHT vs. DMSO	<0.0001
4OHT vs. 4OHT+GCA	<0.0001
4OHT vs. 4OHT+BFA	<0.0001
VEGF	
4OHT vs. DMSO	<0.0001
4OHT vs. 4OHT+GCA	<0.0001
4OHT vs. 4OHT+BFA	<0.0001
CXCL1	
4OHT vs. DMSO	<0.0001
4OHT vs. 4OHT+GCA	<0.0001
4OHT vs. 4OHT+BFA	<0.0001
IL-1β	
4OHT vs. DMSO	<0.0001
4OHT vs. 4OHT+GCA	<0.0001
4OHT vs. 4OHT+BFA	<0.0001
CCL2	
4OHT vs. DMSO	<0.0001
4OHT vs. 4OHT+GCA	<0.0001
4OHT vs. 4OHT+BFA	<0.0001
CCL20	
4OHT vs. DMSO	<0.0001
4OHT vs. 4OHT+GCA	<0.0001
4OHT vs. 4OHT+BFA	<0.0001
GM-CSF	
4OHT vs. DMSO	<0.0001
4OHT vs. 4OHT+GCA	<0.0001
4OHT vs. 4OHT+BFA	<0.0001
G-CSF	
4OHT vs. DMSO	<0.0001
4OHT vs. 4OHT+GCA	<0.0001
4OHT vs. 4OHT+BFA	<0.0001

Supplementary Figure 7d

	DMSO.1	DMSO.2	DMSO.3	4OHT.1	4OHT.2	4OHT.3	4OHT+GCA.1	4OHT+GCA.2	4OHT+GCA.3	4OHT+BFA.1	4OHT+BFA.2	4OHT+BFA.3
IL-6	0.01195607	0.013716	0.007811	1	1	1	0.2599549	0.2359388	0.2151575	0	0	0
IL-8	0	0	0	1	1	1	0.63434391	0.6080976	0.6988626	0.6138193	0.66561841	0.5822848
VEGF	0.28588885	0.023057	0.075044	1	1	1	0.36993697	0.46816831	0.5611927	0.3424627	0.5076792	0.330483
CXCL1	0	0	0	1	1	1	0.32885953	0.36681472	0.2440033	0.2597423	0.26293337	0.2321429
IL-1β	0.01406139	0.004564	0.006737	1	1	1	1.07397958	0.46367405	0.6064849	1.1764839	0.50151925	0.8150238
G-CSF	0.00586132	0.013199	0.032855	1	1	1	0.32937127	0.14888796	0.2903051	0.284289	0.28008277	0.0430635
GM-CSF	0.00797106	0.041589	0.028108	1	1	1	0.60562086	0.35012887	0.4443236	0.5033507	0.428952	0.3616146
CCL2	0.532545767	0.43052	0.577268	1	1	1	0.237276287	0.33329958	0.0825204	0.3188568	0.25164216	0.0671579
CCL20	0.08094156	0.134961	0.112533	1	1	1	0.31081809	0.21324782	0.4376235	0.3435628	0.2054306	0.2052188
LIF	0.025931133	0.042354	0.036872	1	1	1	0.377271751	0.40202006	0.3919561	0.3213229	0.48374881	0.3027105

Dunnett's multiple comparisons test	Adjusted P Value
IL-6	
4OHT vs. DMSO	<0.0001
4OHT vs. 4OHT+GCA	<0.0001
4OHT vs. 4OHT+BFA	<0.0001
IL-8	
4OHT vs. DMSO	<0.0001
4OHT vs. 4OHT+GCA	<0.0001
4OHT vs. 4OHT+BFA	<0.0001
VEGF	
4OHT vs. DMSO	<0.0001
4OHT vs. 4OHT+GCA	<0.0001
4OHT vs. 4OHT+BFA	<0.0001
CXCL1	
4OHT vs. DMSO	<0.0001
4OHT vs. 4OHT+GCA	<0.0001
4OHT vs. 4OHT+BFA	<0.0001
IL-1β	
4OHT vs. DMSO	<0.0001
4OHT vs. 4OHT+GCA	0.0017
4OHT vs. 4OHT+BFA	0.0947
G-CSF	
4OHT vs. DMSO	<0.0001
4OHT vs. 4OHT+GCA	<0.0001
4OHT vs. 4OHT+BFA	<0.0001
GM-CSF	
4OHT vs. DMSO	<0.0001
4OHT vs. 4OHT+GCA	<0.0001
4OHT vs. 4OHT+BFA	<0.0001
CCL2	
4OHT vs. DMSO	<0.0001
4OHT vs. 4OHT+GCA	<0.0001
4OHT vs. 4OHT+BFA	<0.0001
CCL20	
4OHT vs. DMSO	<0.0001
4OHT vs. 4OHT+GCA	<0.0001
4OHT vs. 4OHT+BFA	<0.0001
LIF	
4OHT vs. DMSO	<0.0001
4OHT vs. 4OHT+GCA	<0.0001
4OHT vs. 4OHT+BFA	<0.0001

Source Data for Supplementary Figure 8

Supplementary Figure 8b

	DMSO.1	DMSO.2	DMSO.3	BLEO.1	BLEO.2	BLEO.3	Unpaired t test<br>Adjusted P Value
DMSO	0.045	0.079	1.066	0.66	0.414	0.95	0.742984
ABT-263 1µM	1.614	0.136		0.68	0		0.742984
GCA 2.5µM	2.757	10.214	4.954	24.157	44.879	38.726	0.030114
BFA 150nM	2.552	7.74	2.674	43.898	46.07	35.051	0.002349

Supplementary Figure 8c

	DMSO.1	DMSO.2	DMSO.3	BLEO.1	BLEO.2	BLEO.3
DMSO	0.664	2.005	3.778	23.684	12.824	29.924
GCA 2.5µM	2.981	8.494	13.189	52.233	59.712	37.109
BFA 150nM	2.657	6.635	18.042	57.143	53.716	43.314

Dunnett's multiple comparisons test	
IMR90 DMSO	Adjusted P Value
DMSO vs. GCA 2.5µM	0.5388
DMSO vs. BFA 150nM	0.4537
IMR90 BLEO	Adjusted P Value
DMSO vs. GCA 2.5µM	0.0016
DMSO vs. BFA 150nM	0.001

Supplementary Figure 8d

	DMSO.1	DMSO.2	DMSO.3	BLEO.1	BLEO.2	BLEO.3
DMSO	0.5	1.164	2.28	8.578	6.224	8.176
GCA 2.5µM	0.718	2.036	1.763	30.548	18.601	19.318
BFA 150nM	0.549	1.054	1.437	40.336	20.455	24.307

Dunnett's multiple comparisons test	Adjusted P Value
IMR90 DMSO	
DMSO vs. GCA 2.5µM	0.9985
DMSO vs. BFA 150nM	0.9962
IMR90 BLEO	
DMSO vs. GCA 2.5µM	0.0067
DMSO vs. BFA 150nM	0.0007

Source Data for Supplementary Figure 9

Supplementary Figure 9b

	Washout at 24h						Unpaired t test
	DMSO.1	DMSO.2	DMSO.3	BLEO.1	BLEO.2	BLEO.3	Adjusted P Value
ABT	95.51295	103.7593	99.45152	8.350033	11.64196	9.787736	0.000012
GCA	77.40672	104.8945	101.1444	79.89312	84.87095	83.49057	0.375583
BFA	85.2539	106.5037	104.6994	84.50234	95.00275	81.28931	0.375583

	Washout at 48h										Unpaired t test
	DMSO.1	DMSO.2	DMSO.3	DMSO.4	DMSO.5	BLEO.1	BLEO.2	BLEO.3	BLEO.4	BLEO.5	Adjusted P Value
ABT	100.1331	101.0005	98.30049	95.23302	106.852	15.94447	5.236305	6.949525	2.902903	6.332795	<0.000001
GCA	60.16138	84.32162	83.99015	91.36925	81.73699	34.07657	25.48335	27.3226	24.74975	11.69628	0.000024
BFA	74.45304	91.58126	90.49877	102.5027	83.0034	27.93437	18.52846	17.48354	29.90491	4.297254	0.000011

Supplementary Figure 9c

											Unpaired t test
	DMSO.1	DMSO.2	DMSO.3	DMSO.4	DMSO.5	4OHT.1	4OHT.2	4OHT.3	4OHT.4	4OHT.5	Adjusted P Value
ABT	80.99137	83.78378	69.54165	89.87313	99.74779	45.60949	37.3502	45.08816	48.29264	56.09901	0.000175
GCA	83.34129	80.78961	94.30175	88.56441	91.18742	51.84686	47.21704	59.92163	49.24981	49.84739	0.000009
BFA	79.70626	87.21957	96.90982	89.54669	96.33062	43.58908	49.25433	51.42271	45.40252	39.94236	0.000008

Source Data for Supplementary Figure 10

Supplementary Figure 10a

	DMSO+DMSO.1	DMSO+DMSO.2	DMSO+DMSO.3	4OHT+DMSO.1	4OHT+DMSO.2	4OHT+DMSO.3
% Brdu	41.508	50.275	53.288	7.229	17.27	11.12

	4OHT+GSK-157.1	4OHT+GSK-157.2	4OHT+GSK-157.3	4OHT+GSK-414.1	4OHT+GSK-414.2	4OHT+GSK-414.3
% Brdu	9.168	11.87	11.797	5.601	7.844	8.749

	Ordinary One-Way ANOVA
Dunnett's multiple comparisons test	Adjusted P Value
4OHT vs. DMSO	<0.0001
4OHT vs. GSK157	0.9848
4OHT vs. GSK414	0.4492

Supplementary Figure 10b

	DMSO+DMSO.1	DMSO+DMSO.2	DMSO+DMSO.3	4OHT+DMSO.1	4OHT+DMSO.2	4OHT+DMSO.3
% p16	27.216	27.556	18.863	83.524	73.306	80.524

	4OHT+GSK-157.1	4OHT+GSK-157.2	4OHT+GSK-157.3	4OHT+GSK-414.1	4OHT+GSK-414.2	4OHT+GSK-414.3
% p16	80.01	72.529	79.14	81.879	77.856	82.441

	Adjusted P Value
Dunnett's multiple comparisons test	Ordinary One-Way ANOVA
4OHT vs. DMSO	<0.0001
4OHT vs. GSK157	0.9081
4OHT vs. GSK414	0.9395

Supplementary Figure 10c

	DMSO+DMSO.1	DMSO+DMSO.2	DMSO+DMSO.3	4OHT+DMSO.1	4OHT+DMSO.2	4OHT+DMSO.3
% p21	27.469	31.274	32.6275	79.218	81.5635	81.883

	4OHT+GSK-157.1	4OHT+GSK-157.2	4OHT+GSK-157.3	4OHT+GSK-414.1	4OHT+GSK-414.2	4OHT+GSK-414.3
% p21	80.9595	82.36	83.726	76.4975	79.4455	79.6105

	Adjusted P Value
Dunnett's multiple comparisons test	Ordinary One-Way ANOVA
4OHT vs. DMSO	<0.0001
4OHT vs. GSK157	0.6767
4OHT vs. GSK414	0.3455

Source Data for Supplementary Figure 11

Supplementary Figure 11b

% BRDU	DMSO	ETO		Unpaired t test	
	37.48344	1.916156		P value	0.0003
	31.11888	2.685038			
	39.17397	5.704635			

% sabgal	DMSO	ETO		Unpaired t test	
	0	81.81818		P value	<0.0001
	3.571429	84.21053			
	8.910891	79.54545			

%P21	DMSO	ETO		Unpaired t test	
	7.795792	92.70503		P value	<0.0001
	9.926082	94.01636			
	9.549261	94.02844			

Supplementary Figure 11d

% BRDU	DMSO	ETO		Unpaired t test	
	22.86996	2.285714		P value	0.0007
	16.74208	2.287166			
	18.65672	1.550388			

% sabgal	DMSO	ETO		Unpaired t test	
	1.801802	83.13253		P value	<0.0001
	1.639344	83.5443			
	1.190476	86.56716			

%P21	DMSO	ETO		Unpaired t test	
	15.68627	71.84116		P value	<0.0001
	12.8655	71.74603			
	17.30449	71.63121			

Source Data for Supplementary Figure 12

Supplementary Figure 12a

	(-).1	(-).2	(-).3	IRRD.1	IRRD.2	IRRD.3		Unpaired t test	
% BRDU	35.344	37.482	23.824	1.916	0.153	1.384		P value	0.0019

	(-).1	(-).2	(-).3	IRRD.1	IRRD.2	IRRD.3		Unpaired t test	
% SABGAL	5.940594	7.017544	10.52632	83.56164	94.23077	84.09091		P value	<0.0001

Supplementary Figure 12b

Vector.1	Vector.2	Vector.3	shCOPB2.1	shCOPB2.2	shCOPB2.3	shCOPb2.2	shCOPb2.3	shCOPb2.4
1	1	1	0.1208246	0.07463506	0.102807	0.2642832	0.0842723	0.118211

Dunnett's multiple comparisons test	Adjusted P Value
Vector vs. 1	<0.0001
Vector vs. 2	<0.0001

Supplementary Figure 12c

vector	shCOPA.1	shCOPA.2
1	0.202128	0.124888
1	0.306232	0.168773
1	0.206693	0.195395

Dunnett's multiple comparisons test	Adjusted P Value
vector vs. 1	<0.0001
vector vs. 2	<0.0001

Supplementary Figure 12d

	DMSO.1	DMSO.2	DMSO.3	DOXO.1	DOXO.2	DOXO.3	Unpaired t test Adjusted P Value
Vector	1.012567	1.035457	1.012211	1.007385	1.064859	0.976952	0.897792
shCOPA.1	0.903707	0.946939	0.934907	0.572962	0.627887	0.195556	0.054181
shCOPA.2	0.883287	0.913415	0.978013	0.452726	0.552444	0.175573	0.030368

Supplementary Figure 12e

	LXSN + 4OHT.1	LXSN + 4OHT.2	LXSN + 4OHT.3	RAS+DMSO.1	RAS+DMSO.2	RAS+DMSO.3	RAS + 4OHT.1	RAS + 4OHT.2	RAS + 4OHT.3
Vector	1.096514	1.013306	0.969181	0.965216	1.058318	1.086894	0.917077	0.832346	0.939014
shCOPA.1	1.046848	0.958876	1.004369	0.794742	0.863451	0.818706	0.144738	0.191651	0.13384
shCOPA.2	1.038264	0.983275	1.00967	0.855613	0.740532	0.765547	0.14511	0.200192	0.124077

Adjusted P Value	Unpaired t test
0.058216	LXSN 4OHT vs. RAS 4OHT
0.000021	
0.00002	

Supplementary Figure 12f

	EV							EV+IR						
	Mouse.1	Mouse.2	Mouse.3	Mouse.4	Mouse.5	Mouse.6	Mouse.7	Mouse.1	Mouse.2	Mouse.3	Mouse.4	Mouse.5	Mouse.6	Mouse.7
5	41.65624	22.46357	0	38.81705	75.38215	120.3907	44.02476	47.74327	45.70414	61.07569	94.24677	113.1692	60.14277	33.53163
8	52.00171	32.41262	43.2752	35.193	71.35605	95.00007	64.7087	55.09354	80.22985	55.97361	43.12632	104.6232	76.2486	26.21725
11	60.32859	43.42443	116.5984	39.6548	95.5045	148.5836	82.95956	152.3464	140.917	192.3152	54.74411	191.5106	154.0776	146.8938
14	114.0201	91.02847	178.5558	67.67654	163.2916	137.3455	143.8847	225.4839	255.8741	239.6584	99.34471	189.908	238.7264	194.3368
18	150.9709	157.2267	282.0638	92.50458	209.738	260.7789	192.3152	339.0962	387.0615	200.4862	353.4056	550.2966	463.5412	390.2816
20	204.6571	116.5984			208.46	333.2499	268.7563	357.0448	453.475	257.8286	112.0413	600.4714	390.2816	421.4281
22	208.8854	127.3			248.155	392.2222	162.9306	148.9231	654.5183	506.0631	429.6375	400.7059	407.3145	250.07
26	107.604562	114.3047			306.1213	463.5412	159.7055	133.8349	716.5452	444.2593	475.9634	489.356	574.1834	297.956

	COPB2#1							COPB2#2						
	Mouse.1	Mouse.2	Mouse.3	Mouse.4	Mouse.5	Mouse.6	Mouse.7	Mouse.1	Mouse.2	Mouse.3	Mouse.4	Mouse.5	Mouse.6	Mouse.7
5	99.86439	55.26881	47.74327	65.49146	33.53163	14.14615	47.42569	0	56.15074	47.74327	8.687507	46.16945	77.34097	123.0637
8	82.50038	34.41956	74.30834	48.06227	61.07569	27.29898	69.70467	116.0221	61.07569	29.32034	54.56995	41.07768	99.86439	99.86439
11	195.9641	50.99741	164.7409	234.5627	136.7027	99.34471	124.5657	217.0789	37.71852	48.06227	44.6306	67.07586	157.5793	82.27142
14	342.6367	99.08555	195.5564	564.2171	171.7424	300.6614	219.2701	285.1959	101.6975	121.8709	114.5898	114.3047	239.6584	97.54005
18	538.2055	205.4981	56.863	381.9462	215.7712	405.3243	280.5064	466.4444	117.4664	85.28106	105.7009	162.9306	323.466	135.1045
20	583.4217	99.08555	370.602	655.4303	230.9025	637.3505	263.7517	501.4695	150.9709	131.0075	136.3821	179.3237	277.4088	80.22985
22	593.61223	265.746	444.9637	669.2122	234.5627	633.7749	282.5842	682.2485	149.9446	129.7638	225.9322	162.5701	370.602	162.9306
26	644.541929	185.9407	444.9637	510.6847	381.9462	762.0165	221.919	565.8701	185.1539	132.2592	210.5929	115.4477	420.0701	159.7055

Ordinary Two-way ANOVA

Dunnett's multiple comparisons test (day 22)	Adjusted P Value
Vector + Irrd vs. Vector	0.1222
Vector + Irrd vs. shCOPB2.1 + Irrd	0.9274
Vector + Irrd vs. shCOPB2.2 + Irrd	0.4385

Source Data for Supplementary Figure 13

Supplementary Figure 13d

Time (hr)	DMSO.1	DMSO.2	4OHT.1	4OHT.2	Time (hr)	DMSO.1	DMSO.2	4OHT.1	4OHT.2
0	0	0	0.02	0.05375	82	0	0.00325	1.45425	1.349
2	0	0	0.08425	0.3655	84	0.00325	0.0065	1.739	1.40675
4	0.01	0	0.1895	0.5115	86	0.00325	0.00325	2.02375	1.59
6	0.0065	0	0.264	0.70125	88	0.02	0.00325	2.3595	1.824
8	0.00325	0.00325	0.2875	0.66725	90	0.01	0.00325	2.658	2
10	0.0065	0.0065	0.2705	0.60625	92	0.00675	0.0065	3.092	2.2715
12	0.0065	0.01	0.3315	0.62675	94	0.01	0.0065	3.343	2.624
14	0.0065	0.02	0.33525	0.6575	96	0.00675	0.0065	3.7465	2.96625
16	0.00325	0.0235	0.34175	0.73175	98	0.01675	0.00975	3.977	3.43475
18	0.01	0.0065	0.386	0.6675	100	0.02325	0.0065	4.5565	3.89875
20	0.00975	0.013	0.3725	0.68425	102	0.01675	0.01325	4.96375	4.204
22	0.0335	0.01	0.41325	0.6505	104	0.03025	0.01325	5.347	4.7535
24	0.0435	0.01325	0.393	0.742	106	0.037	0.01325	5.652	5.15325
26	0.03	0.02	0.41	0.69125	108	0.0335	0.01325	5.94725	5.6045
28	0.02325	0.00325	0.42675	0.76225	110	0.0335	0.01325	6.39425	6.01475
30	0.02325	0.01	0.3895	0.68075	112	0.037	0.02	6.774	6.43875
32	0.037	0.02025	0.41675	0.732	114	0.05375	0.02	6.96775	6.81175
34	0.02675	0.02	0.43	0.749	116	0.06725	0.0265	7.2015	7.0285
36	0.01325	0.01325	0.42325	0.698	118	0.0535	0.03	7.415	7.44225
38	0.01	0.013	0.5625	0.72525	120	0.08075	0.0505	7.64575	7.8355
40	0.03	0.01325	0.596	0.73525	122	0.08425	0.047	7.76075	8.1745
42	0.027	0.01675	0.6575	0.725	124	0.108	0.047	7.72375	8.23225
44	0.03025	0.0065	0.64725	0.664	126	0.12825	0.06375	7.9205	8.49325
46	0.0165	0.0065	0.66075	0.8065	128	0.18275	0.06375	7.87275	8.39875
48	0.01	0.0065	0.70125	0.90825	130	0.15525	0.054	7.795	8.4425
50	0.01	0.01675	0.78925	0.85725	132	0.18275	0.0705	7.78475	8.48325
52	0.00675	0.00675	0.79625	0.84725	134	0.2435	0.088	7.754	8.5545
54	0.00975	0.00675	0.871	0.98275	136	0.29775	0.11125	7.744	8.456
56	0.02325	0.013	1.0065	0.96225	138	0.3625	0.12825	7.59475	8.30025
58	0.01	0.0065	0.98625	1.03725	140	0.4435	0.1115	7.5	8.31025
60	0.02325	0.01	1.03025	0.9895	142	0.471	0.14875	7.3505	8.395
62	0.01975	0.00325	1.07125	1.064	144	0.59275	0.169	7.1845	7.8425
64	0.04375	0.00975	1.23725	1.14925	146	0.64375	0.196	7.17775	7.77125
66	0.02	0.0065	1.227	1.13525	148	0.76575	0.2095	7.06575	7.53725
68	0.02675	0.0235	1.25075	1.24725	150	0.88475	0.20975	6.93025	7.34375
70	0	0.02	0.69125	1.237	152	1.0745	0.2335	6.92375	7.1845
72	0	0	0.793	0.691	154	1.322	0.247	6.92	7.26275
74	0	0.00325	0.81675	0.8235	156	1.63725	0.291	6.92025	7.03175
76	0.00325	0	0.96575	0.79275	158	1.86775	0.3725	6.67925	6.98775
78	0	0	1.149	1.00325	160	2.1595	0.464	6.69975	6.9505
80	0	0	1.2335	1.18625	162	2.4445	0.498	6.6315	6.79475

Source Data for Supplementary Figure 14

Supplementary Figure 14b

Ctrl	YnMyr	NMTi	IpaJ WT	IpaJ C64A
1.032562	0.717362	0.041109	0.598318	0.922593
0.57685	1.077486	0.04239	0.360318	1.196234
	1.205152	0.053121	0.221985	1.221752

One-way ANOVA	
Dunnett's multiple comparisons test	Adjusted P Value
YnMyr vs. Ctrl	0.6817
YnMyr vs. NMTi	0.0008
YnMyr vs. IpaJ WT	0.0156
YnMyr vs. IpaJ C64A	0.8938

Supplementary Figure 14c

Ctrl	YnMyr	NMTi	IpaJ WT	IpaJ C64A
0.962421	0.792493	0.168512	1.079808	0.916886
0.881265	1.142771	0.14699	0.978155	0.990542
	1.064736	0.151492	0.738425	1.072212

One-way ANOVA	
Dunnett's multiple comparisons test	Adjusted P Value
YnMyr vs. Ctrl	0.9029
YnMyr vs. NMTi	<0.0001
YnMyr vs. IpaJ WT	0.9111
YnMyr vs. IpaJ C64A	>0.9999

Supplementary Figure 14d

Ctrl	YnMyr	NMTi	IpaJ WT	IpaJ C64A
1.178331	0.896253	0.693729	0.975053	0.750044
0.98943	1.048785	0.629025	0.93232	0.869833
	1.054962	0.582466	0.631752	1.091986

One-way ANOVA	
Dunnett's multiple comparisons test	Adjusted P Value
YnMyr vs. Ctrl	0.9061
YnMyr vs. NMTi	0.0319
YnMyr vs. IpaJ WT	0.4991
YnMyr vs. IpaJ C64A	0.8126

Source Data for Supplementary Figure 15

Supplementary Figure 15b

% Brdu	DMSO	ETO		Unpaired t test	
	90.82744	0.173883		P value	<0.0001
	84.05722	0.519228			
	89.363	0.13463			

% Bgal	DMSO	ETO		Unpaired t test	
	0	88.23529		P value	<0.0001
	0.662252	95.4023			
	2.597403	92.30769			

% p21	DMSO	ETO		Unpaired t test	
	9.043688	92.18168		P value	<0.0001
	8.424475	95.54531			
	8.690546	94.07159			

Supplementary Figure 15d

% BrdU	DMSO	DOXO		Unpaired t test	
	85.81138	0.825333		P value	<0.0001
	78.16305	0.765306			
	74.47872	1.049394			

% Bgal	DMSO	DOXO		Unpaired t test	
	3.846154	94.07895		P value	<0.0001
	1.315789	95.65217			
	3.846154	98.2906			

% p21	DMSO	DOXO		Unpaired t test	
	9.861895	92.59166		P value	<0.0001
	10.61965	93.89068			
	9.117808	92.74887			

Supplementary Figure 15f

% BrdU	DMSO	ETO		Unpaired t test	
	51.35135	2.609514		P value	0.0002
	59.17603	4.275922			
	49.7561	8.819837			

% SABGAL	DMSO	ETO		Unpaired t test	
	8.641975	87.5		P value	<0.0001
	4.166667	91.30435			
	6.25	88.13559			

% p21	DMSO	ETO		Unpaired t test	
	12.06409	91.60369		P value	<0.0001
	22.78708	94.33237			
	13.4606	94.40789			

Supplementary Figure 15h

% Brdu	DMSO	ETO		Unpaired t test	
	48.64865	7.167507		P value	<0.0001
	47.23618	3.842549			
	41.91617	4.828021			

% Bgal	DMSO	DOXO		Unpaired t test	
	5.607477	97.14286		P value	<0.0001
	7.619048	89.21569			
	6.086957	85.85859			

% p21	DMSO	DOXO		Unpaired t test	
	14.29971	92.53112		P value	<0.0001
	14.89486	92.44952			
	15.00519	92.97955			

Source Data for Supplementary Figure 16

Supplementary Figure 16a

Amylase					
Vehicle	912	783	871	869	899
DDD86481	867	744	847	767	831
IMP1320	543	782	859	950	

One way ANOVA

Dunnett's multiple comparisons test	Adjusted P Value
Vehicle vs. DDD8641	0.6087
Vehicle vs. IMP1320	0.3971

Albumin					
Vehicle	44	45	44	43	47
DDD86481	45	43	47	48	47
IMP1320	32	44	47	45	

One way ANOVA

Dunnett's multiple comparisons test	Adjusted P Value
Vehicle vs. DDD8641	0.7961
Vehicle vs. IMP1320	0.5226

ALP					
Vehicle	114	142	145	130	140
DDD86481	132	141	132	172	165
IMP1320	92	162	131	136	

One way ANOVA

Dunnett's multiple comparisons test	Adjusted P Value
Vehicle vs. DDD8641	0.4658
Vehicle vs. IMP1320	0.9422

ALT					
Vehicle	31	32	33	42	46
DDD86481	35	22	29	30	28
IMP1320	16	29	34	31	

One way ANOVA

Dunnett's multiple comparisons test	Adjusted P Value
Vehicle vs. DDD8641	0.1329
Vehicle vs. IMP1320	0.0974

Supplementary Figure 16b

	Glucose				
Vehicle	12.9	12.5	12.5	12.9	13.6
DDD86481	13	10.6	11.4	12.5	12.4
IMP1320	7.6	11.9	13	14.5	

One way ANOVA

Dunnett's multiple comparisons test	Adjusted P Value
Vehicle vs. DDD8641	0.6213
Vehicle vs. IMP1320	0.5233

Supplementary Figure 16c

Vehicle	1.578584	0.613459	1.619455	2.453328	0.938519	
DDD86481	0.367128	1.045083	1.442241	0.657347	2.44842	1.364444

Unpaired t test

P value 0.6262

Supplementary Figure 16d

WBC	24h					1 week		
Vehicle	10.96	5.2	8.22	10.52	8.98	6.64	10.36	9.76
DDD86481	8.26	7.02	8.1	8.42	9.74	7.3	8.38	9.32
IMP1320	6.04	6.2	3.48	7.52	8.24	12.08	10.4	10.4

One way ANOVA

Dunnett's multiple comparisons test	Adjusted P Value
24h	
Vehicle vs. DDD86481	0.8678
Vehicle vs. IMP1320	0.0545
1-week	
Vehicle vs. DDD86481	0.8747
Vehicle vs. IMP1320	0.2543

Lymphocytes	24h					1 week		
Vehicle	9.46	3.94	7.16	9.18	8.02	5.48	9.28	8.68
DDD86481	6.92	6.1	6.74	7.4	8.56	6.82	7.7	8.38
IMP1320	4.92	5.28	2.38	6.58	7.18	11.08	9.5	9.56

One way ANOVA

Dunnett's multiple comparisons test	Adjusted P Value
24h	
Vehicle vs. DDD86481	0.8937
Vehicle vs. IMP1320	0.0725
1-week	
Vehicle vs. DDD86481	0.9867
Vehicle vs. IMP1320	0.1918

Platelets	24h					1 week		
Vehicle	996	1100	1158	1082	1130	344	1020	978
DDD86481	1062	910	1104	1056	1068	1014	824	848
IMP1320	1010	1160	746	1092	1162	1268	1218	1156

One way ANOVA

Dunnett's multiple comparisons test	Adjusted P Value
24h	
Vehicle vs. DDD86481	0.825
Vehicle vs. IMP1320	0.7894
1-week	
Vehicle vs. DDD86481	0.6001
Vehicle vs. IMP1320	0.0079
