# Supplementary material for: COPI vesicle formation and N-myristoylation are targetable vulnerabilities of senescent cells
Source: Nat Cell Biol. 2023 Nov 27;25(12):1804–20. doi: 10.1038/s41556-023-01287-6 (PMC10709147; doi:10.1038/s41556-023-01287-6)
Supplement: Supplementary file 2 — Reporting Summary [file 41556_2023_1287_MOESM2_ESM.pdf]

nature research

Corresponding author(s): JESUS GIL

Last updated by author(s): Jun 29, 2023

Reporting Summary

Nature Research wishes to improve the reproducibility of the work that we publish. This form provides structure for consistency and transparency in reporting. For further information on Nature Research policies, see our Editorial Policies and the Editorial Policy Checklist.

Statistics

For all statistical analyses, confirm that the following items are present in the figure legend, table legend, main text, or Methods section.

n/a	Confirmed	
☐	☒	The exact sample size (*n*) for each experimental group/condition, given as a discrete number and unit of measurement
☐	☒	A statement on whether measurements were taken from distinct samples or whether the same sample was measured repeatedly
☐	☒	The statistical test(s) used AND whether they are one- or two-sided<br>*Only common tests should be described solely by name; describe more complex techniques in the Methods section.*
☒	☐	A description of all covariates tested
☐	☒	A description of any assumptions or corrections, such as tests of normality and adjustment for multiple comparisons
☐	☒	A full description of the statistical parameters including central tendency (e.g. means) or other basic estimates (e.g. regression coefficient) AND variation (e.g. standard deviation) or associated estimates of uncertainty (e.g. confidence intervals)
☐	☒	For null hypothesis testing, the test statistic (e.g. *F*, *t*, *r*) with confidence intervals, effect sizes, degrees of freedom and *P* value noted<br>*Give P values as exact values whenever suitable.*
☒	☐	For Bayesian analysis, information on the choice of priors and Markov chain Monte Carlo settings
☒	☐	For hierarchical and complex designs, identification of the appropriate level for tests and full reporting of outcomes
☒	☐	Estimates of effect sizes (e.g. Cohen's *d*, Pearson's *r*), indicating how they were calculated

Our web collection on statistics for biologists contains articles on many of the points above.

Software and code

Policy information about availability of computer code

Data collection	IN Cell Analyzer 2000 version 5.2-14311 (64-bit) GE Healthcare.<br>Microsoft Excel for Office 365 MSO (16.0.12527.21378) 64-bit<br>IncuCyte Zoom live-cell imager (6.1.7601.65536)
Data analysis	GraphPad Prism 9 (version 9.0.0) for Windows was used for statistical analysis.<br>IN Cell Investigator 1000 workstation 3.7.2, build 1860.<br>ImageJ 2.0.0-rc-54/1.53c.<br>cellHTS2 v2.54.0<br>Tophat v2.0.11.<br>RTA v2.11.3<br>CASAVA 2.17<br>HTSeq v0.5.3p9.<br>DESeq2 v1.30.0<br>GSEA v4.1.0 (Broad Institute)<br>R v4.0.3 (R Foundation for Statistical Computing, Vienna, Austria).<br>Incucyte Zoom Software v1.6

For manuscripts utilizing custom algorithms or software that are central to the research but not yet described in published literature, software must be made available to editors and reviewers. We strongly encourage code deposition in a community repository (e.g. GitHub). See the Nature Research guidelines for submitting code & software for further information.

Data

Policy information about availability of data

All manuscripts must include a data availability statement. This statement should provide the following information, where applicable:

- Accession codes, unique identifiers, or web links for publicly available datasets
- A list of figures that have associated raw data
- A description of any restrictions on data availability

RNAseq data have been deposited in the Gene Expression Omnibus (GEO) under accession codes GSE224070, GSE224071 and GSE224069. Source data have been provided in Source Data. All other data supporting the findings of this study are available from the corresponding author on reasonable request.

Field-specific reporting

Please select the one below that is the best fit for your research. If you are not sure, read the appropriate sections before making your selection.

☒ Life sciences ☐ Behavioural & social sciences ☐ Ecological, evolutionary & environmental sciences

For a reference copy of the document with all sections, see nature.com/documents/nr-reporting-summary-flat.pdf

Life sciences study design

All studies must disclose on these points even when the disclosure is negative.

Sample size	No statistical methods were used to pre-determine sample sizes, but our sample sizes are similar to those reported previously (e.g. Guerrero et al Nature Aging 2022; Guerrero et al Nature Metabolism 2019; Triana-Martinez et al Nature Comm 2019; Georgilis et al Cancer Cell 2018)
Data exclusions	For the bleomycin-induced fibrosis experiment, two mice in the vehicle-treated group died before the end of the experiment and were not included in any analysis. For the WD experiment, a mouse of the WD + vehicle group died before the end of the experiment and was not included in any analysis. RNA isolation from one mouse liver of the WD +NMTi group failed and it could not be included in subsequent RT-qPCR analysis. Three sections from Figure 8h were excluded due to poor section processing. No further data were excluded from analyses.
Replication	All experiments were reproducible. Every figure states how many times each experiment was performed with similar results.
Randomization	For in vivo studies mice were randomized to treatment groups.<br>Cell culture experiments did not require normalization because the tests were compared to controls. Plates needed to be marked to ensure the treatments are delivered to the appropriate plates (and not the control) and randomisation would not be practical or feasible.
Blinding	Histologic analysis Masson's trichrome and hematoxilin & eosin staining on mouse fibrosis experiment was examined first in a blinded fashion and in a second round in an unblinded fashion. Tumour measurements were taken blindly and independently by the researcher or the animal technicians in the mouse facility.For the HDTVI and WD experiments, staining and analysis was performed in a blinded fashion. Investigators were not blinded during the other experiments.

Reporting for specific materials, systems and methods

We require information from authors about some types of materials, experimental systems and methods used in many studies. Here, indicate whether each material, system or method listed is relevant to your study. If you are not sure if a list item applies to your research, read the appropriate section before selecting a response.

Materials & experimental systems

n/a	Involved in the study	
☐	☒	Antibodies
☐	☒	Eukaryotic cell lines
☒	☐	Palaeontology and archaeology
☐	☒	Animals and other organisms
☒	☐	Human research participants
☒	☐	Clinical data
☒	☐	Dual use research of concern

Methods

n/a	Involved in the study	
☒	☐	ChIP-seq
☒	☐	Flow cytometry
☒	☐	MRI-based neuroimaging

Antibodies

Antibodies used	The following primary antibodies were used in this study: mouse monoclonal anti-BrdU (Clone 3D4, BD Biosciences, cat.555627) 1:2000, mouse monoclonal anti-p16INK4a (Clone.JC8, Gifted from CRUK) 1:1000, rabbit polyclonal anti-GAPDH (Abcam, cat.ab22555) 1:2000, mouse monoclonal anti-IL8 (clone.6217, R&D systems, cat.MAB208) 1:100, goat polyclonal anti-IL6 (R&D Systems, cat.AF-206-NA) 1:40-1:200, mouse monoclonal anti-ARF1/3/5/6 (clone.1D9, Invitrogen, MA3-060) 1:500, rabbit monoclonal anti-

COPB2 (clone.899, Not commercially available, Gifted from F. Weiland) 1:10000, mouse monoclonal anti-EEA1 (clone.14, BD Biosciences, cat.610457) 1:200, rabbit polyclonal anti-XBP1 (Abcam, cat.ab37152) 1:200, rabbit polyclonal anti-ATF6 (Abcam, cat.ab37149) 1:500, sheep polyclonal anti-TGN46 (BioRad, cat.AHP500G) 1:400, mouse monoclonal anti-GM130 (clone.35, BD Biosciences, cat.610822) 1:500, mouse monoclonal anti-CHOP (clone.L63F7, Cell Signaling Technology, cat.2895S) 1:1000, rabbit monoclonal anti-p21CIP1 (clone.12D1, CST, cat.2947S) 1:2000, rabbit monoclonal anti-p21CIP1 (clone.EPR18021, Abcam, cat.ab188224) 1:700, mouse monoclonal anti-N-Ras (clone.F155, Santa Cruz, cat.sc-31) 1:100, mouse monoclonal anti-β-Catenin (clone.6F9, Sigma-Aldrich, cat.C7082) 1:500, rabbit polyclonal anti-β-Catenin (ThermoFisher, cat.RB-9035-P1) 1:500, mouse monoclonal anti-Synaptophysin (clone.27G12, Leica, cat.SYNAP-299-L) 1:200, rabbit polyclonal anti-Cleaved-Caspase-3 (Cell Signaling Technology, cat.9661S) 1:1000, goat polyclonal anti-CXCL1 (R&D Systems, cat.AF-275) 1:100, mouse monoclonal anti-BMP2/4 (clone.100230, R&D Systems, cat.MAB3552), mouse monoclonal anti-VEGF (clone.23410, R&D Systems, cat.MAB2931) 1:100, Mouse monoclonal anti-GM-CSF (clone.3209, R&D Systems, cat.MAB215) 1:100, rabbit polyclonal anti-CD68 (Abcam, cat.ab125212) 1:100. Rabbit polyclonal anti-ARF1 (cat.10790-1-AP, Proteintech) 1:1000, rabbit polyclonal anti-ARL1 (cat.16012-1-AP, Proteintech) 1:1000, rabbit polyclonal anti-PPM1B (cat.HPA-016745, Cambridge Bioscience) 1:1000 and rabbit polyclonal, mouse monoclonal anti-TUBA (cat.ab1729, Abcam), 1:1000.

We used the following secondary antibodies: goat anti-mouse IgG-HRP (Santa Cruz, cat.sc-2005) 1:2000, goat anti-rabbit IgG-HRP (Santa Cruz, cat.sc-2004) 1:2000, goat anti-mouse IgG (H+L) AlexaFluor488 conjugated (Invitrogen, cat.A-11029) 1:2000, goat anti-mouse IgG (H+L) AlexaFluor594 conjugated (Invitrogen, cat.A-11032) 1:2000, goat anti-rabbit IgG (H+L) AlexaFluor594 conjugated (Invitrogen, cat.A-11037) 1:2000, donkey anti-sheep IgG (H+L) AlexaFluor594 conjugated (Invitrogen, cat.A-11016) 1:2000, donkey anti-sheep IgG (H+L) AlexaFluor488 conjugated (Invitrogen, cat.A-11015) 1:2000. For the IpaJ western blot experiments, we used the following secondary antibodies: IRDye 800CW goat anti-rabbit IgG (H+L) (cat.926-32211, Li-Cor) 1:10000 and IRDye 800CV goat anti-mouse IgG (H+L) (cat.926-32210, Li-Cor) 1:10000.

Validation

BrdU
https://www.abcam.com/brdu-antibody-bu175-icr1-proliferation-marker-ab6326.html
GAPDH
https://www.abcam.com/gapdh-antibody-loading-control-ab22555.html
IL8
https://www.rndsystems.com/products/human-il-8-cxcl8-antibody-6217_mab208 / Georgilis et al. 2018
Il6
https://www.rndsystems.com/products/human-il-6-antibody_af-206-na / Georgilis et al. 2018
ARF1/3/5/6
https://www.thermofisher.com/antibody/product/ARF1-ARF3-ARF5-ARF6-Antibody-clone-1D9-Monoclonal/MA3-060
P16-Ink4a (JC-8)
https://www.scbt.com/p/p16-antibody-jc8
COPB2
Validation from immunoblot of lysates from IMR90 ER:RAS cells transduced with COPB2 shRNA (Supplemental FIgure 2A)
EEA1
https://www.bdbiosciences.com/us/reagents/research/antibodies-buffers/cell-biology-reagents/cell-biology-antibodies/purified-mouse-anti-eea1-14eea1/p/610457
XBP1
https://www.abcam.com/xbp1-antibody-ab37152.html
ATF6
https://www.abcam.com/atf6-antibody-ab37149.html
TGN46
https://www.bio-rad-antibodies.com/polyclonal/human-tgn46-antibody-ahp500.html?f=purified
GM130
https://www.bdbiosciences.com/us/reagents/research/antibodies-buffers/cell-biology-reagents/cell-biology-antibodies/purified-mouse-anti-gm130-35gm130/p/610822
CHOP
https://www.cellsignal.co.uk/products/primary-antibodies/chop-l63f7-mouse-mab/2895
p21 (12D1)
https://www.cellsignal.co.uk/products/primary-antibodies/p21-waf1-cip1-12d1-rabbit-mab/2947
p21 (EPR18021)
https://www.abcam.com/p21-antibody-epr18021-ab188224.html
N-RAS
https://www.scbt.com/p/n-ras-antibody-f155 / Amor et al. 2020
β-Catenin (6F9)
https://www.thermofisher.com/antibody/product/beta-Catenin-Antibody-clone-6F9-Monoclonal/MA1-300
β-Catenin (RB-9035-P1)
https://www.thermofisher.com/order/catalog/product/RB-9035-P?uk&en#/RB-9035-P?uk&en
Synaptophysin
https://shop.leicabiosystems.com/us/ihc-ish/ihc-primary-antibodies/pid-synaptophysin
Cleaved-Caspase-3
https://www.cellsignal.co.uk/products/primary-antibodies/cleaved-caspase-3-asp175-antibody/9661
CXCL1
https://www.rndsystems.com/products/human-cxcl1-groalpha-kc-cinc-1-antibody_af275
BMP2/4
https://www.rndsystems.com/products/human-bmp-2-bmp-4-antibody-100230_mab3552
VEGF
https://www.rndsystems.com/products/human-vegf-antibody-23410_mab2931
GM-CSF
https://www.rndsystems.com/products/human-gm-csf-antibody-3209_mab215
CD68
https://www.abcam.com/products/primary-antibodies/cd68-antibody-ab125212.html

ARF1
https://www.ptglab.com/products/ARF1-Antibody-10790-1-AP.htm
ARL1
https://www.ptglab.com/products/ARL1-Antibody-16012-1-AP.htm
PPM1B
https://www.sigmaaldrich.com/US/en/product/sigma/hpa016745
alpha-Tubulin
https://www.abcam.com/products/primary-antibodies/alpha-tubulin-antibody-dm1a-loading-control-ab7291.html

Eukaryotic cell lines

Policy information about cell lines

Cell line source(s)

IMR-90, SK-HEP-1, A549, HCT-116, MCF-7, PBEC cells were obtained from ATCC. NHLF cells were obtained from Lonza. HFFF2 cells were obtained from ECACC. 5PT cells were a gift from Professor IC Mackenzie (Queen Mary University of London).

Authentication

IMR-90, SK-HEP-1 and A549 cells were authenticated in 2019 using DNA short tandem repeat profiling (Eurofins).

Mycoplasma contamination

All cell lines were routinely tested for mycoplasma contamination and were negative.

Commonly misidentified lines
(See ICLAC register)

None of the cell lines used in this study is present in the database of commonly misidentified cell lines.

Animals and other organisms

Policy information about studies involving animals; ARRIVE guidelines recommended for reporting animal research

Laboratory animals

All mice were kept under specific pathogen-free barrier conditions within individually ventilated cages on a 12-hour light/dark cycle at a temperature of 21 +/- 2oC and humidity 45-65%. Mice were given ad libitum access to food and water.
HDTVI experiments were performed on female C57BL/6J mice (5-6 weeks old).
Cancer xenograft experiments performed on male NOD.Cg-Prkdcscid Il2rgtm1Wjl/SzJ (3-5 months old).
Ex vivo neoplastic pituitaries were isolated from Hesx1Cre/+;Ctnnb1lox(ex3)/+ embryos on a C57 background (18.5 dpc).
Lung fibrosis experiments described in Fig 5 were performed on 6-8 weeks old, athymic (nu/nu) male mice.
Bleomycin-induced lung fibrosis experiments were performed on 6-8 weeks old, male C57BL/6J mice.
Western diet experiments were performed on male C57BL/6J (8-weeks old).

Wild animals

No wild animals were used in this study.

Field-collected samples

This study did not involve samples collected in the field.

Ethics oversight

Our research complies with all relevant ethical regulations and guidelines. The lung fibrosis experiments were performed in compliance with guidelines established by the Barcelona Science Park's Committee on Ethics for Animal Experimentation (CEEA) and under approved protocol number 10884. All other mouse procedures were performed under licence, according to UK Home Office Animals (Scientific Procedures) Act 1986, ARRIVE, and local institutional guidelines. The mouse pituitary experiments were approved by the UCL ethical review committee (PPL P5FB9D417). Liver cancer initiation and the Western diet experiments were approved by the animal welfare and ethical review board at Imperial College London (PPL 70/09080 and PPL PE02064666 respectively). Cancer xenograft experiments were performed by national and international guidelines and were approved by the institutional review board at Southampton University (PPL P81E129B7).

Note that full information on the approval of the study protocol must also be provided in the manuscript.
