# Supplementary material for: COPI vesicle formation and N-myristoylation are targetable vulnerabilities of senescent cells
Source: Nat Cell Biol. 2023 Nov 27;25(12):1804–20. doi: 10.1038/s41556-023-01287-6 (PMC10709147; doi:10.1038/s41556-023-01287-6)
Supplement: Supplementary file 10 — Unprocessed western blots. [file 41556_2023_1287_MOESM10_ESM.pdf]

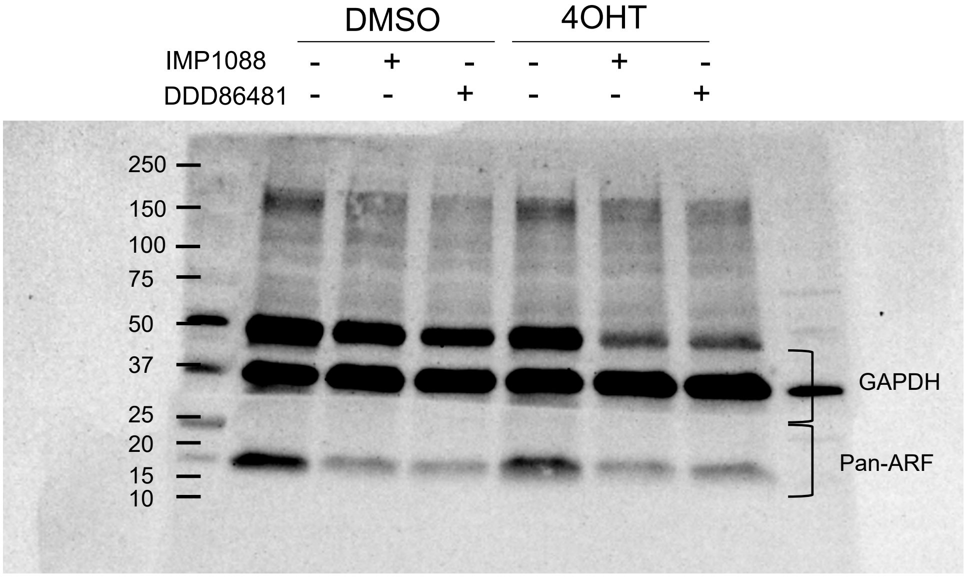

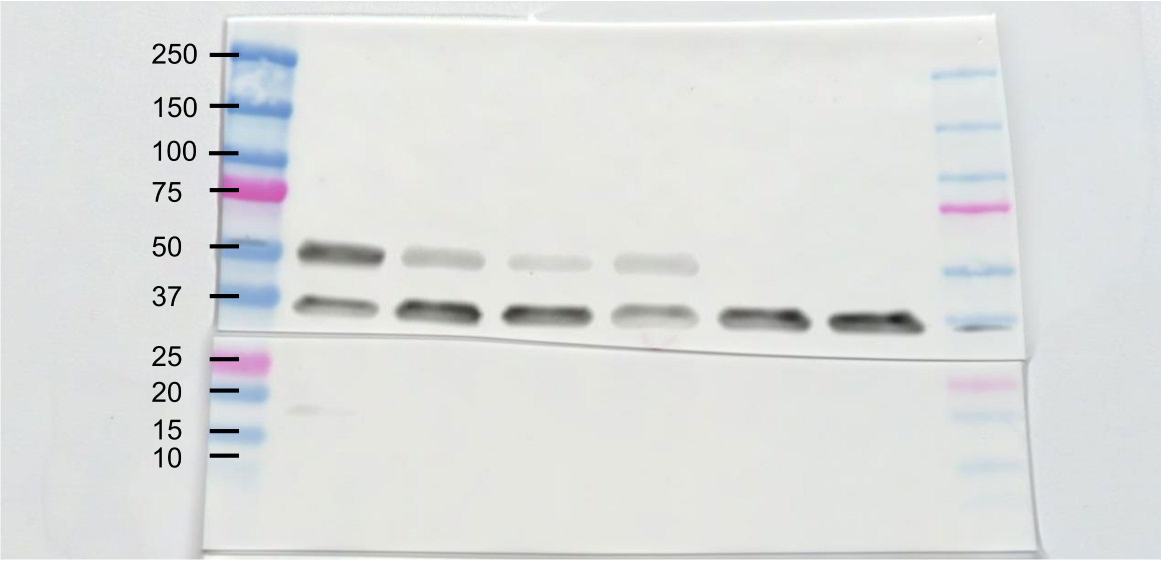

Uncropped images of western blots shown in Figure 6a
The molecular weights (kDa) of size markers are indicated.
